# Enantioselective hydrogenation of annulated arenes: controlled formation of multiple stereocenters in adjacent rings†

**DOI:** 10.1039/d0sc07099h

**Published:** 2021-03-04

**Authors:** Mario P. Wiesenfeldt, Daniel Moock, Daniel Paul, Frank Glorius

**Affiliations:** Westfälische Wilhelms-Universität Münster, Organisch-Chemisches Institut Corrensstraße 40 48149 Münster Germany glorius@uni-muenster.de

## Abstract

We report a method for the enantioselective hydrogenation of annulated arenes using 4*H*-pyrido[1,2-*a*]pyrimidinones as substrates. The method selectively generates multiple stereocenters in adjacent rings leading to architecturally complex motifs, which resemble bioactive molecules. The mechanistic study of the stereochemical outcome revealed that the catalyst is able to overcome substrate stereocontrol providing all-*cis*-substituted products predominantly. In a sequential protocol, a matching interaction between catalyst and substrate stereocontrol is achieved that facilitates diastereo- and enantioselective access to *trans*-products.

## Abstract 2

We report a method for the enantioselective hydrogenation of annulated arenes using 4*H*-pyrido[1,2-*a*]pyrimidinones as substrates.

## Graphical abstract

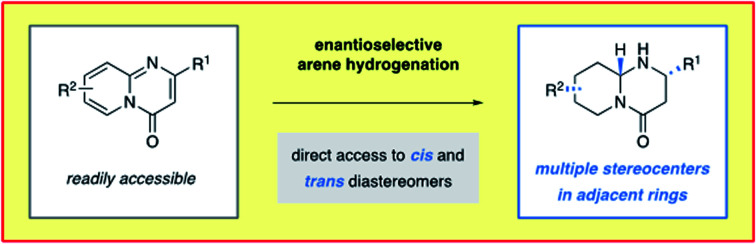

Chiral, saturated carbo- and heterocycles are important structural elements of secondary metabolites and active ingredients in drugs and agrochemicals.^1^ Compared to classical synthetic routes that utilize prefunctionalized precursors,^2^ enantioselective arene hydrogenation provides a more direct route to such motifs from readily accessible (hetero-) aromatic substrates.^3^ With current hydrogenation technologies, a variety of monocyclic arenes such as pyridines,^4^ furans,^5^ thiophenes,^6^ and annulated arenes such as quinolines and isoquinolines,^7^ indoles,^8^ and naphthalenes^9^ can be hydrogenated with high enantioselectivity. However, in all previous reports on the enantioselective hydrogenation of annulated arenes (**1**) the resulting products (**2**) contain only a single saturated ring with at least one aromatic sextet being preserved in the product, and consequently, with stereocenter(s) only being formed in one ring (Fig. 1A).^10^ An enantioselective hydrogenation of multiple annulated aromatic rings would enable the generation of multiple stereocenters at adjacent rings in a single step, leading to desirable, architecturally complex and natural product-like motifs. Herein, we report the first such example using N-bridged 4*H*-pyrido[1,2-*a*]pyrimidinone (pyrido-pyrimidinone) substrates (**3**, Fig. 1B).^10*a*–*d*^ Pyrido-pyrimidinones, and their saturated analogs, are featured in bioactive molecules (Fig. 1C). For instance, Roche's Risdiplam (**5**), an inhibitor against spinal muscular atrophy, includes a pyrido-pyrimidinone unit^11^ and a semi-reduced tetrahydropyrido-pyrimidinone is a part of risperidone (**6**), a blockbuster drug against schizophrenia, while octahydropyrido-pyrimidinones (**4**) are structurally closely related to quinolizidine alkaloids such as lupanine (**7**). To date, only a small number of octahydropyrido-pyrimidinones (**4**) have been accessed with the majority resulting from the hetero-geneous, racemic hydrogenation of tetrahydropyrido-pyrimidinones (**8**). To the best of our knowledge, enantioenriched octahydropyrido-pyrimidinones have never been accessed by enantioselective catalysis.^12^

**Fig. 1:**
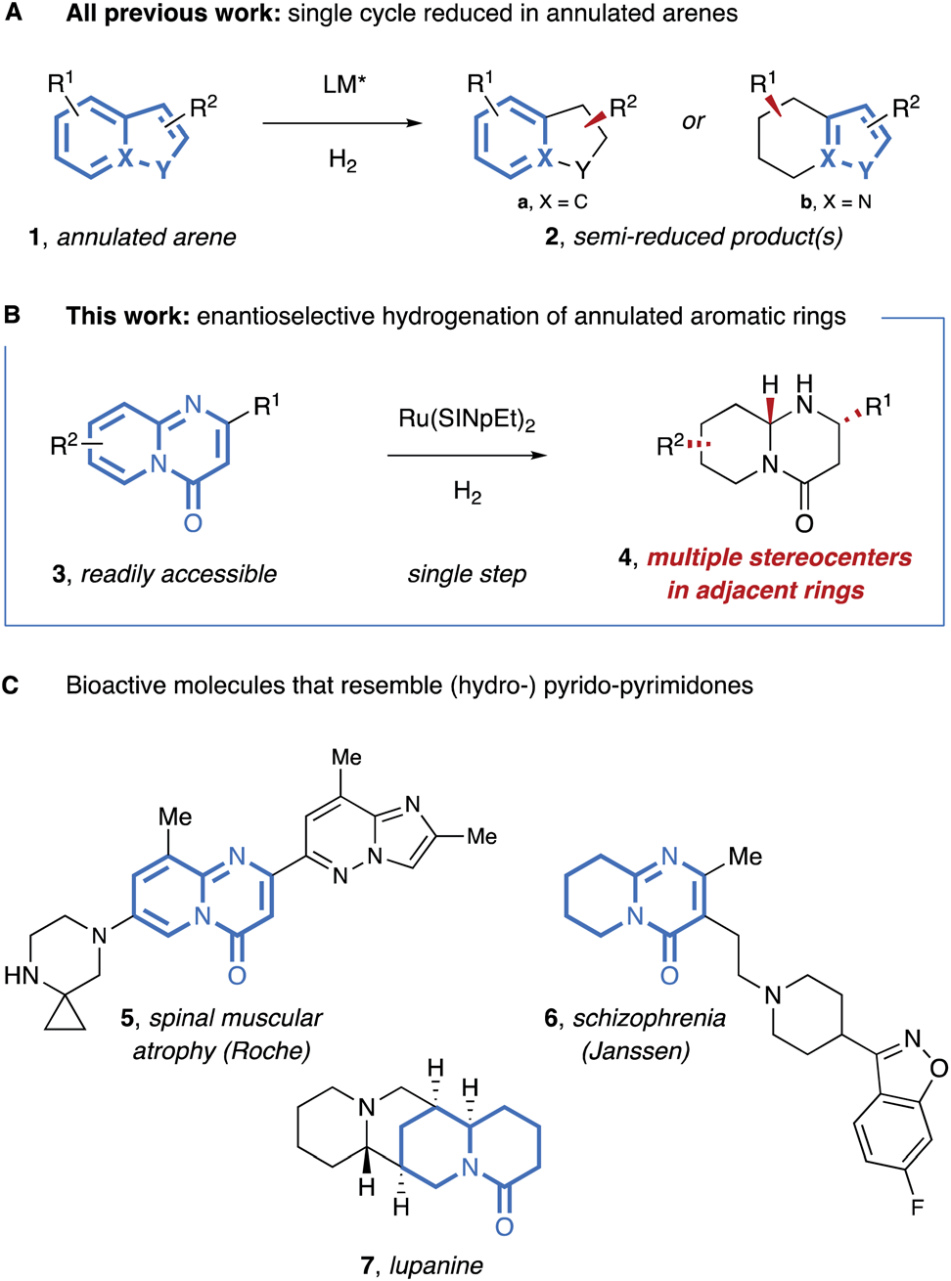
(A) Previously: only one ring of annulated arenes reduced by enantioselective hydrogenation. (B) This work: enantioselective hydrogenation of both rings of bicyclic aromatic pyrido-pyrimidinones (**3**). (C) Biologically active examples/analogues of pyrido-pyrimidinones and their hydrogenated products.

We realized that an enantioselective hydrogenation of the readily accessible pyrido-pyrimidinones would in principle offer the most direct approach towards these valuable product motifs, provided that the chemoselectivity could be controlled and potential side products, such as tetrahydro- (**8**), hexahydropyrido-pyrimidinones **9** and **10**, and the presumably unstable hemiaminal **11** resulting from amide reduction, could be avoided (Fig. 2).^13^ Furthermore, we rationalized that up to four different diastereomers could result from the creation of three independent stereocenters, making the simultaneous control of chemo-, diastereo-, and enantioselectivity a daunting challenge. Nevertheless, we were ideally positioned to overcome this challenge given the high reactivity, enantioselectivity, and broad tolerance of functional groups shown by the chiral ruthenium-bisNHC catalyst system developed by our group (**12**).^14,15^ Indeed, we observed that catalytic hydrogenation of model substrate 2,7-dimethylpyrido-pyrimidinone (**3a**) proceeded with near-complete chemoselectivity under a variety of reaction conditions (Table S1†). Under the optimized conditions, the desired octahydropyrido-pyrimidinone was obtained in 92% yield as a mixture of just two from the possible four diastereomers (63 : 37 diastereomeric ratio (dr)) and with excellent 96% enantiomeric excess (ee) for the major diastereomer (Table S1,† entry 4). The evaluation of other substitution patterns of the pyrido- pyrimidinone using the respective dimethyl-substituted substrates revealed that the 2,8-disubstituted product can also be obtained with good chemo-, enhanced diastereo-, and moderate enantioselectivity. However, substrates with 2,6- and 2,9-substitution patterns were less reactive. Only the eastern pyridine ring was reduced in 48% and 82% yield, respectively (Table S2†). As such we decided to focus on substrates with a 2,7-substitution pattern when exploring the scope of this reaction (Fig. 3). Next, the scope was evaluated (Fig. 3). Substrates without a substituent in the C2-position (**3b**), and substrates containing sterically more hindering substituents such as a benzyl or an *iso*-propyl group (**3c,d**) in the C2-position all delivered the products in high yields as a mixture of just two diastereomers with moderate dr and excellent ee values for the major diastereomer. The dr values increase with the steric size of the C2-substituent. A phenyl substituent in the C2-position rendered the (eastern) pyrimidinone ring inactive, thus chemoselectively providing tetrahydropyrido-pyrimidinone **8e** with moderate ee under the standard reaction conditions including the reaction time of 48 h. Contrastingly, the introduction of aryl groups in the 7-position of the (western) pyridine ring was well-tolerated. Consequently, octahydropyrido-pyrimidinones carrying aryl groups such as phenyl (**4f**), sterically demanding *ortho*-tolyl (**4g**), and other aryl groups bearing various synthetically useful functional groups such as NHBoc, OCF_3_, or Cl in the *para*-position (**4h–j**) can all be accessed in high yields and ee values. Curiously, while 7-*para*-fluorophenyl-substituted product **4k** was obtained in high yield, the enantioselectivity was reduced in comparison to its chlorine-containing counterpart **4j**. Products without a substituent in the 7-position (**4l–n**) were isolated in good yields as single diastereomers with good to high ee.

**Fig. 2:**
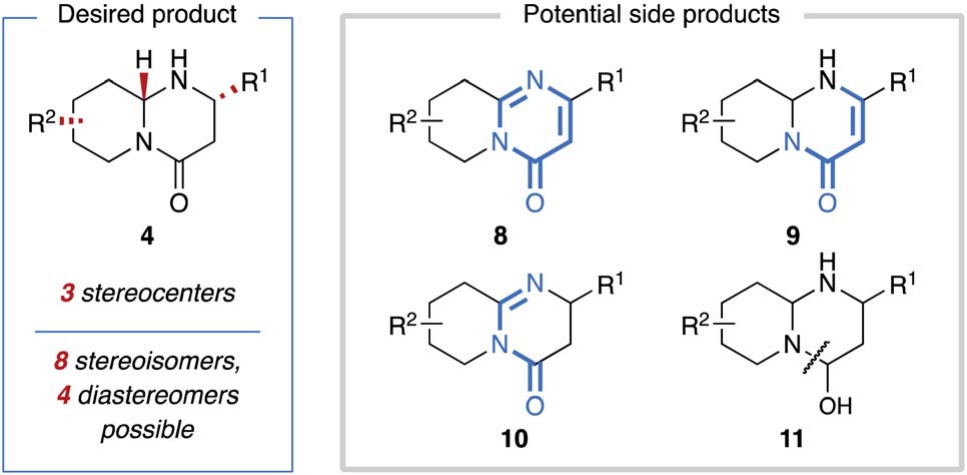
Chemo- and stereoselectivity challenges.

**Fig. 3:**
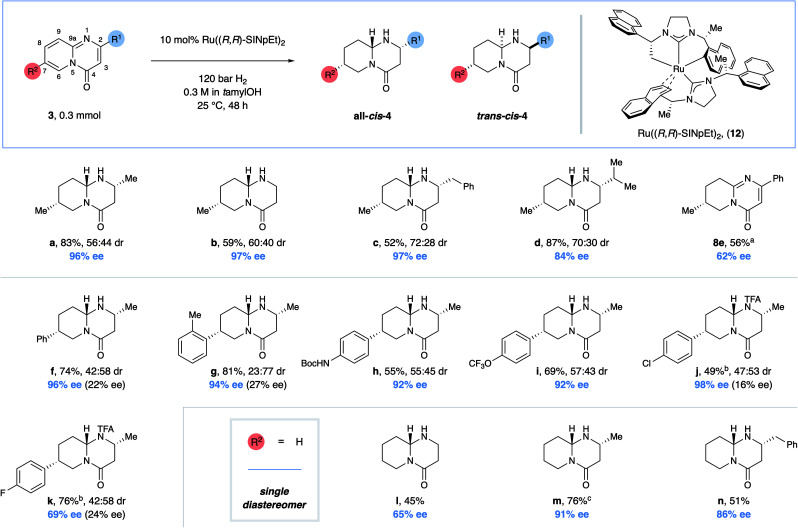
Scope. Combined isolated yields of both diastereomers are reported. The dr was determined by NMR-spectroscopy. The major diastereomer (and enantiomer) was assigned by X-ray crystallographic analysis of both diastereomers of **4a**. The assignment for the other products was conducted by analogy. The ee of the minor diastereomer was measured in all cases (see ESI†). ^a^Traces of an unknown impurity could not be separated from the product. ^b^Protection with trifluoroacetic anhydride. ^c^80 bar H_2_ used. SINpEt: 1,3-bis(1-(naphthalene-1-yl)ethyl)-4,5-dihydroimidazolylidene; TFA: trifluoroacetate.

Intrigued by those observations, we proceeded to study the stereochemical mechanism of the reaction. First, we determined the reaction progress of the hydrogenation of model substrate **3a** over time (Fig. 4A). After a reaction time of 1.5 h, tetrahydropyrido-pyrimidinone **8a** is almost the exclusive species before its relative abundance decreases with a coinciding increase of the proportion of octahydropyrido-pyrimidinone **4a**. Notably, 7-methyl- and 7-phenyl-substituted intermediates **8a** and **8f** were isolated in good yields and with moderate enantiomeric excess (**8a**: 84% yield, 63% ee; **8f**: 50% yield, 62% ee). The reaction is complete after 8 h, leaving octahydrogenated product 4a as the exclusive species. Hence **8a** must be an intermediate in the hydrogenation of **3a** to **4a**. No further signals, other than those allocated to the substrate **3a** and tetrahydrointermediate **8a**, were observed in the enone or α-ketone regions at any of the time points. Furthermore, the C2 and C9a C–H bonds are *cis*-aligned in both diastereomers of the 2,7-dimethylproduct **4a** (confirmed by X-ray crystallography, see ESI†). A *cis* alignment would be enforced by a non-interrupted coordination of the catalyst during the hydrogenation of the (eastern) pyrimidinone ring. The codependence of the configurations of C2 and C9a would result in the formation of only two diastereomers in the hydrogenation of 7-substituted substrates and only a single diastereomer in the hydrogenation of 7-unsubstituted substrates, which is in agreement with the observations made during the exploration of the scope (Fig. 4B).^16^ Both observations suggest that enone **9a** and imine **10a** are not intermediates in this reaction. Finally, we determined that two separate effects cause the (relatively low) diastereomeric ratios of 7-substituted octahydropyrido-pyrimidinones. Firstly, the enantiocontrol over the initially formed C7-stereocenter is only moderate (**8a**: 63% ee after 1.5 h, Fig. 4B). Secondly, the ee of the intermediates **8** increases with the reaction progress of further hydrogenation towards the octahydroproducts 4 (Fig. 4C). It follows that the minor enantiomer of chiral intermediate **8** is converted into octahydro-product **4** more rapidly than the major enantiomer, indicating a mismatched interaction between substrate and catalyst control. To further test this conclusion, isolated intermediate **8a** was submitted to the enantioselective hydrogenation using the opposite enantiomer of the chiral catalyst in the second hydrogenation step (Fig. 4D).^16^ In this case, hydrogenation proceeds with a matched interaction between catalyst and substrate control and provides the opposite diastereomer **trans–cis-4a** with excellent diastereo- and enantiocontrol. Notably even racemic arene hydrogenation rarely produces trans-products.^18^ The dr of **all–cis-4a** derived from the two-step hydrogenation (Fig. 4D, red equation) is higher than that of the standard one-pot protocol (75 : 25 *vs* 56 : 44 dr). This is a result of the increase of the ee of intermediate **8a** as a function of the reaction time (the minor enantiomer is consumed faster to form **4a**). The effective ee of **8a** in the two-step protocol (isolated after 2 h) was higher than that of **8a** in the standard protocol resulting in a higher dr of **4a**.

**Fig. 4:**
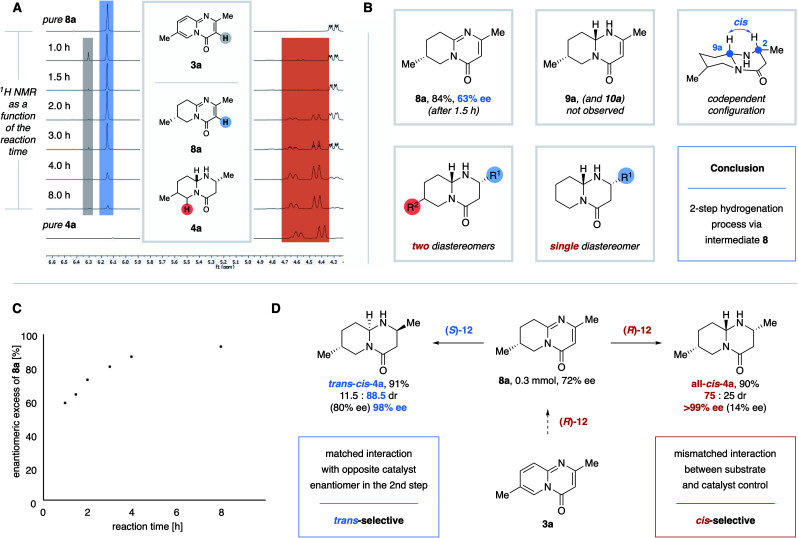
Mechanistic studies. (A) Excerpt of the ^1^H NMR spectrum of the reaction mixture as a function of the reaction time. (B) Key observations that suggest a two-step hydrogenation process. (C) ee of **8a** as a function of the reaction time. (D) Sequential enantioselective hydrogenation of **3a** using opposite enantiomers of the chiral catalyst.^17^

## Conclusions

In conclusion, we disclose the first method for the enantioselective hydrogenation of multiple annulated arenes forming stereocenters in adjacent rings. Using pyrido-pyrimidones as substrates, the method produces architecturally complex, natural product-like motifs and routinely forms three stereocenters at adjacent rings selectively in a single operation. Mechanistic investigation of the stereochemical outcome suggests a two-step hydrogenation pathway involving a catalyst dissociation/reassociation step. The catalyst is able to overcome the substrate stereocontrol in the second hydrogenation step leading predominantly to all-*cis*-substituted products. This mis-matched interaction can be converted into a matched interaction by switching the enantiomer of the chiral catalyst in the second step in a two-pot operation that provides the opposite, *trans*-diastereomer with excellent diastereo- and enantiocontrol.

## Author contributions

M. P. W., D. M., and D. P. performed the synthetic experiments and developed the project. F. G. supervised the research and M. P. W., D. M., D. P., and F. G. wrote the manuscript.

## Conflicts of interest

The authors declare no competing financial interests.

## Supplementary Material

SC-012-D0SC07099H-s001

SC-012-D0SC07099H-s002
