# Supplementary material for: Enantioselective hydrogenation of annulated arenes: controlled formation of multiple stereocenters in adjacent rings
Source: Chem Sci. 2021 Mar 4;12(15):5611–5. doi: 10.1039/d0sc07099h (PMC8179591; doi:10.1039/d0sc07099h)
Supplement: SC-012-D0SC07099H-s001 [file SC-012-D0SC07099H-s001.pdf]

Electronic Supplementary Material (ESI) for Chemical Science.
This journal is © The Royal Society of Chemistry 2021

Enantioselective Hydrogenation of Annulated Arenes: Controlled Formation of Multiple Stereocenters in Adjacent Rings

*Mario P. Wiesenfeldt,[‡] Daniel Moock,[‡] Daniel Paul,[‡] and Frank Glorius**

Organisch-Chemisches Institut, Westfälische Wilhelms-Universität Münster, Corrensstraße 36, 48149 Münster, Germany

E-mail: glorius@uni-muenster.de

Supporting Information

Contents

(A) Materials and methods **4**

(B) Synthesis of the catalyst **6**

Synthesis of the chiral SINpEt·HBF_4 precursors 6

Synthesis of the Ru(SINpEt)$_2$ catalysts **12** 7

(C) Synthesis of starting materials **8**

Synthesis of 2-unsubstituted substrates 8

Condensation of 2-aminopyridines with β-ketoesters 8

Suzuki coupling 10

(D) Optimization **13**

Discussion 14

(E) Determination of the optimal substitution pattern **15**

(F) Scope of the enantioselective hydrogenation of pyrido-pyrimidinones **17**

(G) Monitoring of the reaction progress **43**

Procedure 43

Results 44

Discussion 46

(H) Sequential hydrogenation **48**

Procedure 48

Results 48

Discussion 49

(I) NMR spectra of new substrates **50**

(J) NMR spectra of products **59**

(K) X-ray diffraction data **75**

Molecular structure of **all-*cis*-4a** • 2 HCl 75

Molecular structure of ***trans*-*cis*-4a** 76

(L) References **78**

(A) Materials and methods

Unless otherwise noted, all reactions were carried out under an argon atmosphere in oven- or heat-gun-dried glassware. Reaction temperatures are reported as the temperature of the medium surrounding the vessel unless otherwise stated. The employed solvents were dried by distillation over the drying agents indicated in parentheses and were transferred under argon: diethyl ether (Na-benzophenone), *n*-hexane (CaH_2), THF (Na-benzophenone), toluene (CaH_2). *tert*-Amyl alcohol (*t*amylOH, 4 Å), dioxane (4 Å), ethanol (3 Å), ethyl acetate (4 Å), and trifluoroethanol (4 Å) were purchased as dry solvents from commercial suppliers and stored over molecular sieves.

All hydrogenation reactions were carried out in Berghof High Pressure Reactors using hydrogen gas. Commercially available chemicals were obtained from ABCR, Acros Organics, Aldrich Chemical Co., Alfa Aesar, Chempur, Combi-Blocks, and TCI Europe and used as received unless otherwise stated. (*R*)- and (*S*)-1-(1-naphthyl)ethylamine were obtained from BASF (ChiPros®). Unless otherwise noted, no optimizations of the yields were performed for substrate syntheses. The analytical data are given for all previously unknown substrates.

Analytical thin layer chromatography was performed on Polygram SIL G/UV_{254} plates. Visualization was accomplished with short wave UV light and $KMnO_4$ staining solutions followed by heating. Flash chromatography was performed on Merck silica gel (40–63 mesh) by standard techniques eluting with solvents as indicated.

^{1}H and ^{13}C NMR spectra were recorded on a Bruker AV 300 or AV 400, Varian 500 MHz INOVA, or Varian Unity plus 600 in the indicated solvents. Chemical shifts (δ) are given in ppm relative to TMS. The residual solvent signals were used as references and the chemical shifts were converted to the TMS scale ($CDCl_3$: δ_H = 7.26 ppm, δ_C = 77.16 ppm; CD_2Cl_2: δ_H = 5.32 ppm, δ_C = 53.84 ppm). ESI mass spectra were recorded on a Bruker Daltonics MicroTof spectrometer. APCI mass spectra were recorded on a Thermo Fisher Scientific Orbitrap LTQ XL. No IR spectra for substrates or products were recorded. Products were obtained as mixtures of diastereomers and hence IR spectroscopic analysis was deemed to not give useful

information. Measurement of IR spectra was attempted for a variety of substrates. However, the obtained IR spectra had a very low intensity. It is likely that the fluffy nature of the solids precluded an effective measurement. X-Ray diffraction data sets for compound **all-*cis*-4a** were collected with a Bruker APEX II CCD diffractometer. Programs used: data collection: APEX3 V2016.1-0 (Bruker AXS Inc., **2016**);[1] cell refinement: SAINT V8.37A (Bruker AXS Inc., **2015**); data reduction: SAINT V8.37A (Bruker AXS Inc., **2015**);[2] absorption correction: SADABS V2014/7 (Bruker AXS Inc., **2014**);[3] structure solution SHELXT-2015 (Sheldrick, **2015**);[4] structure refinement SHELXL-2015 (Sheldrick, **2015**).[5] For compound ***trans*-*cis*-4a**, data sets were collected with a Nonius Kappa CCD diffractometer. Programs used: data collection: COLLECT (Hooft, Bruker AXS, **2008**);[6] data reduction: Denzo-SMN (Otwinoswski, Minor, **1997**);[7] absorption correction: Denzo (Otwinowski, Borek, Majewski, Minor, **2003**);[8] structure solution: SHELXT-2015 (Sheldrick, **2015**);[4] structure refinement: SHELXL-2015 (Sheldrick, **2015**),[5] and graphics XP (Bruker AXS Inc., **1998**).[9] *R*-values are given for observed reflections and wR^2 values are given for all reflections. Enantiomeric ratios of chiral chemicals were determined on an Agilent Technologies 6890N Gas Chromatograph with a Supelco β-Dex column (chir. GC) or on an Agilent Technologies 7890B Gas Chromatograph with an Astec Chiraldex G-TA column. The method indicated as 100_5_0.5_180_5_1_220_10 starts at 100 °C, which is maintained for 5 min before heating with a rate of 0.5 °C/min to 180 °C, which is maintained for 5 min before further heating with a rate of 1 °C/min to 220 °C, which is maintained for 10 min. Alternative methods are indicated in the same notation together with the characterization data of the enantioenriched products. Alternatively, enantiomeric ratios were determined using an Agilent Technologies 1200 Series HPLC with Daicel Chemical Industries LTD Chiralpak AD-H, AS-H or Chiralcel OD-H, OJ-H columns (0.46 cm x 25 cm). The signals were detected by UV-absorption spectroscopy (at 210 nm, 230 nm, or 254 nm).

(B) Synthesis of the catalyst

Synthesis of the chiral SINpEt·HBF_4 precursors

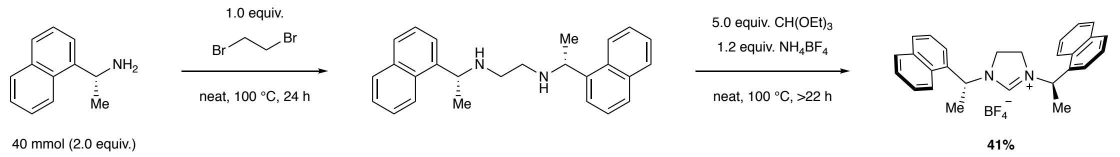

According to a modified literature procedure[10] (*R*)-1-(1-naphthyl)ethylamine (6.42 mL, 6.85 g, 40.0 mmol, 2.0 equiv.) and dibromoethane (1.73 mL, 3.76 g, 20.0 mmol, 1.0 equiv.) were mixed in a dry Schlenk tube, which was flushed with argon, sealed, and heated to 100 °C. The mixture forms a highly viscous oil and stops stirring after a few hours, yet it should be heated for roughly 24 h. The resulting viscous oil was taken up in CH_2Cl_2 (50 mL) and 1 M aqueous NaOH, supersonicated for 5–10 min to dissolve the viscous oil, basified to pH ≈ 13 using 2 M aqueous NaOH, and extracted with further CH_2Cl_2 (3 x 30 mL). The combined organic layers were dried ($MgSO_4$) and concentrated in vacuo to furnish the respective diamine, which was used in the next step without further purification.

A mixture of $CH(OEt)_3$ (16.6 mL, 14.8 g, 100 mmol, 5.0 equiv.), the chiral diamine, and NH_4BF_4 (2.52 g, 24.0 mmol, 1.2 equiv.) in a dry Schlenk tube was heated to 120 °C for (at least) 22 h. After cooling to room temperature, the crude product was filtered and washed with hexane (in total ca. 30 mL) and Et_2O (in total ca. 30 mL). Precipitation can be induced by addition of a few milliliters of Et_2O in case it does not occur directly during the reaction. Purification by recrystallization from MeOH (ca. 250 mL) provided the desired product as a white solid. Yield 41% (3.84 g, 8.23 mmol).

An analogous synthesis was conducted for the (*S*)-SINpEt·HBF_4 precursor starting from the corresponding (*S*)-1-(1-naphthyl)ethylamine.

Synthesis of the Ru(SINpEt)$_2$ catalysts **12**

According to a slightly modified literature procedure,[10] a suspension of the imidazolium precursor (280 mg, 600 μmol, 2.0 equiv.), [Ru(COD)(2-methylallyl)$_2$] (95.9 mg, 300 μmol, 1.0 equiv.), and KO*t*Bu (70.8 mg, 630 μmol, 2.1 equiv.) was stirred in hexane (10 mL) at 70 °C for 16–20 h under argon atmosphere to give a yellow-to-orange stock suspension that was used as catalyst without further purification. The catalyst is air-sensitive and decomposes slowly over time. While it performs best when prepared freshly, it can be used for a few weeks without significant loss of reactivity provided that contact with air is excluded by the use of standard Schlenk techniques.

The employed achiral Ru–ICy catalyst was prepared via an analogous procedure starting from the corresponding imidazolium chloride.

(C) Synthesis of starting materials

Synthesis of 2-unsubstituted substrates

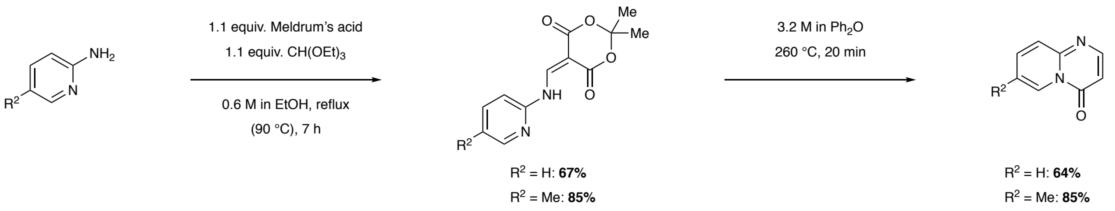

According to a modified literature procedure,[11] a mixture of the amino pyridine (1 equiv.), meldrum's acid (1.1 equiv.), and $HC(OEt)_3$ (1.1 equiv.) was refluxed in EtOH (0.6 M) for 7 h in a dry Schlenk tube under argon atmosphere. After cooling down to room temperature, the mixture was filtered. The residue was washed with ethanol and dried to give the intermediate as red crystals.

According to a modified literature procedure,[11] the intermediate (1 equiv.) was distributed to three 9 mL screw-cap vials, dissolved in Ph_2O (3.2 M), and stirred at 260 °C for 20 min in a preheated metal-heating block. After cooling down to room temperature, the mixtures were combined and purified by column chromatography (EtOAc, later EtOAc/MeOH = 9:1).

The obtained analytical data were in good agreement with the literature.[11]

Condensation of 2-aminopyridines with β-ketoesters

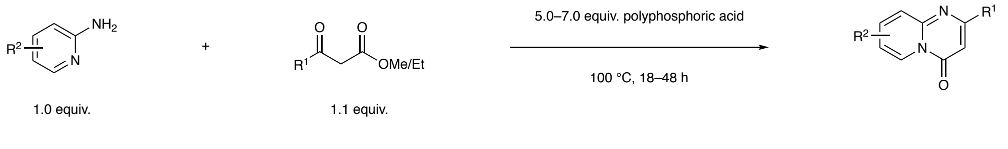

According to a slightly modified literature procedure,[12] a mixture of the 2-aminopyridine (1.0 equiv.), the β-ketoester (1.1 equiv.), and polyphosphoric acid (ca. 5.0–7.0 equiv.) was stirred at 100 °C for 18–48 h (18 h are generally sufficient). After cooling down to room temperature, CH_2Cl_2 and ice were added, and the emulsion was carefully basified with conc. aqueous NaOH with continuous addition of further ice and CH_2Cl_2. The basified mixture was transferred to a separating funnel with CH_2Cl_2 and water, the phases were separated, and the

aqueous layer was extracted with additional CH_2Cl_2 (3x). The combined organic extracts were dried over $MgSO_4$, concentrated, and purified by column chromatography and/or recrystallization.

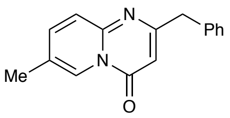

3c, orange solid, 3.55 mmol scale. 42% yield. Purification by column chromatography (pentane/EtOAc = 1:4, gradient to pure EtOAc). ^{1}H NMR (300 MHz, $CDCl_3$) δ 8.77 (q, *J* = 1.4 Hz, 1H), 7.70–7.44 (m, 2H), 7.37–7.14 (m, 5H), 6.18 (s, 1H), 3.96 (s, 2H), 2.36 (d, *J* = 1.2 Hz, 3H); ^{13}C NMR (75 MHz, $CDCl_3$) δ 167.18, 158.24, 149.92, 139.23, 137.77, 129.47, 128.79, 126.92, 125.73, 125.51, 124.77, 103.22, 44.67, 18.42. ESI-MS: calculated $[C_{16}H_{14}N_2O+H]^+$: 251.1179, found: 251.1184. Melting point: 115 °C.

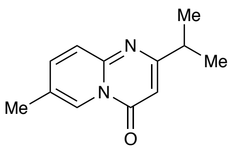

3d, off-white solid, 5.00 mmol scale. 74% yield. Purification by column chromatography (pentane/EtOAc = 1:4, gradient to pure EtOAc). ^{1}H NMR (400 MHz, $CDCl_3$) δ 8.85–8.77 (m, 1H), 7.60–7.47 (m, 2H), 6.37–6.29 (m, 1H), 2.95–2.82 (m, 1H), 2.43–2.31 (m, 3H), 1.34–1.25 (m, 6H); ^{13}C NMR (101 MHz, $CDCl_3$) δ 173.68, 158.65, 149.93, 138.85, 125.77, 125.16, 124.68, 100.50, 36.47, 21.81, 18.40; ESI-MS: calculated $[C_{12}H_{14}N_2O+Na]^+$: 225.0998, found: 225.0993. Melting point: 71 °C.

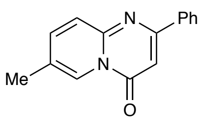

3e, orange solid, 9.25 mmol scale. 49% yield. Purification by recrystallization from MTBE. ^{1}H NMR (400 MHz, $CDCl_3$) δ 8.87 (dq, *J* = 2.2, 1.1 Hz, 1H), 8.10–8.01 (m, 2H), 7.66 (d, *J* = 9.0 Hz, 1H), 7.59 (dd, *J* = 9.0, 2.1 Hz, 1H), 7.54–7.40 (m, 3H), 6.89 (s, 1H), 2.42 (d, *J* = 1.2 Hz, 3H); ^{13}C NMR (101 MHz, $CDCl_3$) δ 161.75, 158.65, 150.07, 139.23, 137.49, 130.60, 128.89, 127.45, 126.31, 125.63, 124.81, 99.96, 18.46. ESI-MS: calculated $[C_{15}H_{12}N_2O+Na]^+$: 259.0842, found: 259.0848. Melting point: 169 °C.

Suzuki coupling

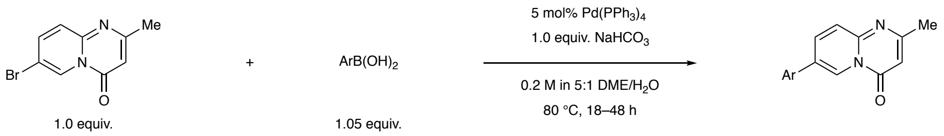

According to a slightly modified literature procedure,[13] a mixture of the bromo-4*H*-pyrido[1,2-*a*]pyrimidin-4-one (1.0 equiv.), arylboronic acid (1.05 equiv.), 1 M aqueous $NaHCO_3$ (1.0 equiv.), and $Pd(PPh_3)_4$ (5 mol%) in DME (0.2 M) was stirred at 80 °C until complete conversion of the starting material was observed (18–48 h, TLC). After cooling to room temperature, the mixture was extracted with CH_2Cl_2 (3x), dried over $MgSO_4$, concentrated, and purified by column chromatography. In several cases, subsequent recrystallization was performed. Measurement of IR spectra was attempted for a variety of substrates. However, the obtained IR was of very low intensity. It is likely that the fluffy nature of the solids precluded an effective measurement.

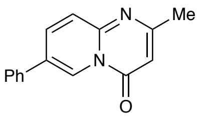

3f, white solid, 4.12 mmol scale. 91% yield. Purification by column chromatography (pentane/EtOAc = 1:4, gradient to pure EtOAc). ^{1}H NMR (400 MHz, $CDCl_3$) δ 9.23 (d, *J* = 2.3 Hz, 1H), 8.00 (dd, *J* = 9.2, 2.2 Hz, 1H), 7.81–7.59 (m, 3H), 7.58–7.37 (m, 3H), 6.36 (s, 1H), 2.48 (s, 3H); ^{13}C NMR (101 MHz, $CDCl_3$) δ 165.16, 158.03, 149.91, 136.34, 135.59, 129.47, 129.13, 128.95, 126.99, 126.07, 124.34, 103.50, 24.83. ESI-MS: calculated $[C_{15}H_{12}N_2O+H]^+$: 237.1022, found: 237.1036. Melting point: 155 °C.

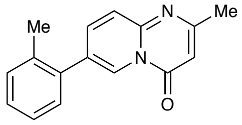

3g, yellow solid, 2.00 mmol scale. 58% yield. Purification by column chromatography (pentane/EtOAc = 1:4, gradient to pure EtOAc). ^{1}H NMR (500 MHz, $CDCl_3$) δ 9.00 (m, 1H), 7.74 (dd, *J* = 9.1, 2.1 Hz, 1H), 7.63 (dd, *J* = 9.1, 0.8 Hz, 1H), 7.38–7.26 (m, 4H), 6.37 (s, 1H), 2.49 (s, 3H), 2.32 (s, 3H); ^{13}C

NMR (126 MHz, $CDCl_3$) δ 165.21, 158.00, 149.82, 138.65, 135.93, 135.82, 130.95, 129.95, 129.74, 128.97, 126.50, 126.08, 125.16, 103.50, 24.81, 20.44. ESI-MS: calculated $[C_{16}H_{14}N_2O+Na]^+$: 273.0998, found: 273.0997. Melting point: 127 °C.

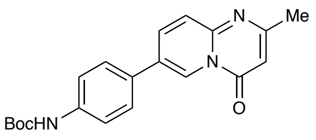

3h, white solid, 4.00 mmol scale. 21% yield. 1.60 equiv. of the boronic acid, 30 mol% catalyst, and an extended reaction time of 96 h were used. Purification by column chromatography (CH_2Cl_2/EtOAc = 1:1 gradient to pure EtOAc). ^{1}H NMR (400 MHz, CD_2Cl_2) δ 9.17 (d, *J* = 2.2 Hz, 1H), 8.00 (dd, *J* = 9.2, 2.2 Hz, 1H), 7.63–7.60 (m, 3H), 7.52 (d, *J* = 8.7 Hz, 2H), 6.74 (s, 1H), 6.29 (s, 1H), 2.43 (s, 3H), 1.52 (s, 9H); ^{13}C NMR (101 MHz, CD_2Cl_2) δ 165.06, 158.08, 152.67, 149.78, 139.41, 136.21, 130.57, 129.88, 128.74, 127.59, 125.99, 123.64, 119.14, 103.41, 81.09, 28.46, 24.82. ESI-MS: calculated $[C_{20}H_{21}N_3O_3+H]^+$: 352.1656, found: 352.1659. Melting point: 187 °C.

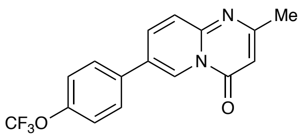

3i, white solid, 4.00 mmol scale. 27% yield. 1.60 equiv. of the boronic acid and an extended reaction time of 96 h were used. Purification by precipitation from the reaction media and subsequent recrystallization from EtOAc. ^{1}H NMR (300 MHz, CD_2Cl_2) δ 9.19 (d, *J* = 2.3 Hz, 1H), 7.96 (dd, *J* = 9.2, 2.2 Hz, 1H), 7.79–7.68 (m, 2H), 7.64 (dd, *J* = 9.3, 0.8 Hz, 1H), 7.44–7.34 (m, 2H), 6.31 (s, 1H), 2.44 (s, 3H); ^{13}C {^{19}F} NMR (126 MHz, CD_2Cl_2) δ 165.69, 158.02, 150.28, 149.93, 136.06, 135.02, 128.90, 127.81, 126.64, 124.76 (m), 122.07, 120.91, 103.49, 24.84; ^{19}F NMR (282 MHz, CD_2Cl_2) δ −58.21. ESI-MS: calculated $[C_{16}H_{11}F_3N_2O_2+H]^+$: 321.0845, found: 321.0849. Melting point: 165 °C.

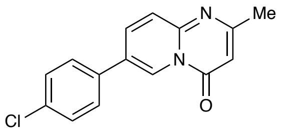

3j, white solid, 2.09 mmol scale. 51% yield. Purification by column chromatography (pentane/EtOAc = 1:4, gradient to pure EtOAc) and subsequent recrystallization from EtOAc. ^{1}H NMR (400 MHz, $CDCl_3$) δ 9.22 (d, *J* = 2.2 Hz, 1H), 7.95 (dd, *J* = 9.2, 2.2 Hz, 1H), 7.67 (dd, *J* = 9.2, 0.8 Hz, 1H),

7.64–7.54 (m, 2H), 7.56–7.41 (m, 2H), 6.38 (s, 1H), 2.49 (s, 3H); ^{13}C NMR (126 MHz, $CDCl_3$) δ 165.21, 157.90, 149.82, 135.99, 135.30, 134.05, 129.74, 128.25, 128.03, 126.23, 124.38, 103.67, 24.79. ESI-MS: calculated $[C_{15}H_{11}ClN_2O+H]^+$: 271.0633, found: 271.0640. Melting point: 208 °C.

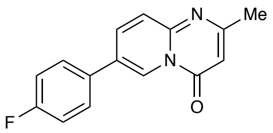

3k, white solid, 2.09 mmol scale. 76% yield. Purification by column chromatography (EtOAc) and subsequent recrystallization from EtOAc. ^{1}H NMR (300 MHz, $CDCl_3$) δ 9.19 (d, *J* = 2.2 Hz, 1H), 7.95 (dd, *J* = 9.2, 2.2 Hz, 1H), 7.89–7.51 (m, 3H), 7.26–7.09 (m, 2H), 6.37 (s, 1H), 2.48 (s, 3H); ^{13}C {^{19}F} NMR (126 MHz, $CDCl_3$) δ 165.17, 163.33, 157.92, 149.76, 136.14, 131.71, 128.78, 128.21, 126.12, 124.15, 116.53, 103.56, 24.76; ^{19}F NMR (376 MHz, $CDCl_3$) δ −112.60 (tt, *J* = 8.4, 5.1 Hz). ESI-MS: calculated $[C_{15}H_{11}FN_2O+H]^+$: 255.0928, found: 255.0932. Melting point: 186 °C.

(D) Optimization

The preformed Ru((*R*,*R*)-SINpEt)$_2$ catalyst (**12**) was added as stock suspension (1.0 mL, 10 mol%) in hexane to an oven-dried 9 mL screw-cap vial equipped with a stirring bar and the substrate (0.3 mmol) under argon atmosphere. The solvent was carefully evaporated under reduced pressure before the reaction solvent (1 mL) was added. The vial was stirred vigorously until a uniform suspension of substrate and catalyst in the solvent was observed. The glass vial was placed in a 150 mL stainless steel autoclave under argon atmosphere. The autoclave was pressurized and depressurized with hydrogen gas three times before the pressure was set. The reaction mixture was stirred at 25 °C for 48 h. After the autoclave was carefully depressurized, the solvent was removed under reduced pressure. The residue was taken up in CH_2Cl_2 (10 mL) and extracted with 1 M aqueous HCl (4 x 7 mL). The combined aqueous phases were washed with CH_2Cl_2 (2 x 5 mL), basified with 3 M aqueous NaOH, and extracted with CH_2Cl_2 (5 x 15 mL). The combined organic extracts were washed with water (10 mL), dried over $MgSO_4$, and concentrated. 0.3 mL of CD_2Cl_2 and 0.33 equiv. of mesitylene (12.0 mg, 13.9 μL) were added and the mixture was stirred. 0.2 mL of that mixture were transferred into an NMR tube before additional 0.5 mL of CD_2Cl_2 were added and the yield and dr were measured by ^{1}H NMR spectroscopy against the internal standard mesitylene. The remaining product solution was dissolved in additional CH_2Cl_2 (1 mL). Trifluoroacetic anhydride (42.0 μL, 63.0 mg, 0.3 mmol, 3.0 equiv.) was added to the solution. The mixture was stirred for 15 min at room temperature and concentrated. The mixture was filtered over a silica plug (eluent: pentane/EtOAc = 2:1, 10 mL) and concentrated before the ee was measured. The racemate was prepared in an analogous fashion by replacing Ru((*R*,*R*)-SINpEt)$_2$ **12** with Ru(ICy)$_2$ on a 0.1 mmol scale at 60 °C.

Table S1. Reaction optimization.

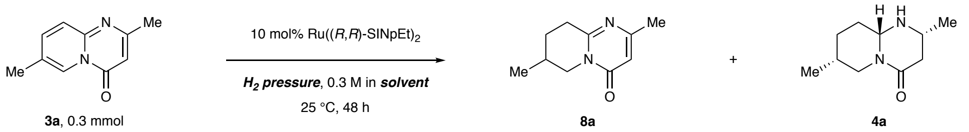

Entry	Solvent	H_2 pressure [bar]	Conversion [%]	Yield **8a** [%]	Yield of **4a** [%] (dr, ee **major**/ee minor in [%])
1	hexane	120	100	0	87 (57:43 dr, **94**/50)
2	$PhCF_3$	120	99	1	64 (67:33 dr, **96**/56)
3	toluene	120	100	0	78 (58:42 dr, **95**/46)
4	*t*amylOH	120	100	0	92 (63:37 dr, **96**/29)
5	*t*amylOH	80	100	0	89 (65:35 dr, **96**/34)
6	*t*amylOH	50	100	0	90 (60:40 dr, **97**/36)

The reactions were conducted at a 0.3 mmol scale using 10 mol% Ru((*R*)-SINpEt)$_2$ under the indicated pressure 0.3 M in the indicated solvent at 25 °C for 48 h.

Discussion

The catalytic hydrogenation reaction proceeded with near-complete chemoselectivity, moderate diastereoselectivity and excellent enantioselectivity for the major diastereomer in a variety of solvents at hydrogen pressures ranging from 50–120 bar. Ultimately, *tert*-Amyl under 120 bar hydrogen pressure with an extended reaction time of 48 h proved superior for less reactive substrates thus prompting our decision to use these conditions as standard reaction conditions for the exploration of the scope.

(E) Determination of the optimal substitution pattern

The preformed Ru((*R*,*R*)-SINpEt)$_2$ catalyst (**12**) was added as stock suspension (1.0 mL, 10 mol%) in hexane to an oven-dried 9 mL screw-cap vial equipped with a stirring bar and the substrate (0.3 mmol) under argon atmosphere. The solvent was carefully evaporated under reduced pressure before *t*amylOH (1 mL) was added. The vial was stirred vigorously until a uniform suspension of substrate and catalyst in the solvent was observed. The glass vial was placed in a 150 mL stainless steel autoclave under argon atmosphere. The autoclave was pressurized and depressurized with hydrogen gas three times before the pressure was set to 120 bar. The reaction mixture was stirred at 25 °C for 24 h. After the autoclave was carefully depressurized, *t*amylOH was removed under reduced pressure. The residue was taken up in CH_2Cl_2 (10 mL) and extracted with 1 M aqueous HCl (4 x 7 mL). The combined aqueous phases were washed with CH_2Cl_2 (2 x 5 mL), basified with 3 M aqueous NaOH, and extracted with CH_2Cl_2 (5 x 15 mL). The combined organic extracts were washed with water (10 mL), dried over $MgSO_4$, and concentrated. C7- and C8-substituted crude products were dissolved in CH_2Cl_2 and purified by column chromatography (CH_2Cl_2/EtOAc/MeOH = 50:45:5, gradient to 45:45:10). After measuring the amount, the dr and product ratios were determined by NMR spectroscopy and the product was dissolved in CH_2Cl_2 (3 mL). For entries 2 and 3: trifluoroacetic anhydride (100 μL, 151 mg, 0.72 mmol, 7.2 equiv.) was added to a sample (1 mL) of the solution. The mixture was stirred for 15 min at room temperature and concentrated. The mixture was filtered over a silica plug (eluent: pentane/EtOAc = 1:1, 10 mL) and concentrated before the ee was measured. Racemates were prepared in an analogous fashion by replacing Ru((*R*,*R*)-SINpEt)$_2$ **12** with Ru(ICy)$_2$ on a 0.1 mmol scale at 60 °C.

Table S2. Determination of the optimal substitution pattern.

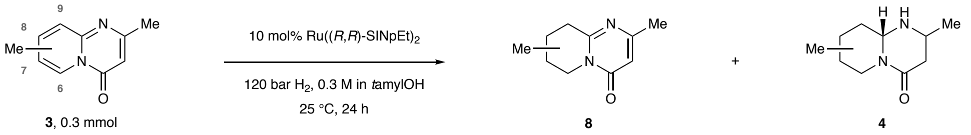

Entry	Substituted carbon	Yield **3** in [%]	Yield **8** in [%]	Yield **4** in [%] (dr, ee **major**/ee minor in [%])
1	C6	24	48	0
2[a]	C7	0	13	60 (63:37, **96**/2)
3[a]	C8	0	7	54 (70:30, **54**/96)
4	C9	12	82	0

The reactions were conducted at a 0.3 mmol scale using 10 mol% Ru((*R*)-SINpEt)$_2$ under 120 bar H_2 pressure 0.3 M in *t*amylOH at 25 °C for 24 h. [a]The product mixture was purified by column chromatography as indicated in the procedure.

(F) Scope of the enantioselective hydrogenation of pyrido-pyrimidinones

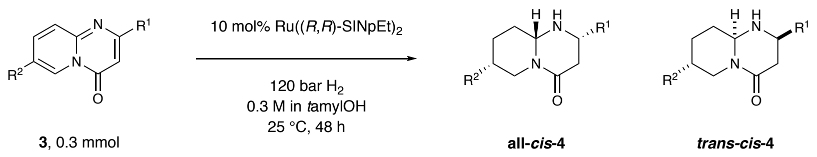

General procedure 1:

The preformed Ru((*R*,*R*)-SINpEt)$_2$ catalyst (**12**) was added as stock suspension (1.0 mL, 10 mol%) in hexane to an oven-dried 9 mL screw-cap vial equipped with a stirring bar and the substrate (0.3 mmol) under argon atmosphere. The solvent was carefully evaporated under reduced pressure before *t*amylOH (1 mL) was added. The vial was stirred vigorously until a uniform suspension of substrate and catalyst in the solvent was observed. The glass vial was placed in a 150 mL stainless steel autoclave under argon atmosphere. The autoclave was pressurized and depressurized with hydrogen gas three times before the pressure was set to 120 bar. The reaction mixture was stirred at 25 °C for 48 h. After the autoclave was carefully depressurized, *t*amylOH was removed under reduced pressure. The residue was taken up in CH_2Cl_2 (10 mL) and extracted with 1 M aqueous HCl (4 x 7 mL). The combined aqueous phases were washed with CH_2Cl_2 (2 x 5 mL), basified with 3 M aqueous NaOH, and extracted with CH_2Cl_2 (5 x 15 mL). The combined organic extracts were washed with water (10 mL), dried over $MgSO_4$, and concentrated. Some products were purified by column chromatography (see characterization data of individual products for details). After measuring the yield, the product was dissolved in CH_2Cl_2 (3 mL). Trifluoroacetic anhydride (100 μL, 151 mg, 0.72 mmol, 7.2 equiv.) was added to a sample (1 mL) of the solution. The mixture was stirred for 15 min at room temperature and concentrated. The mixture was filtered over a silica plug (eluent: pentane/EtOAc = 1:1, 10 mL) and concentrated before (the dr and) the ee was measured. Racemates were prepared in an analogous fashion by replacing Ru((*R*,*R*)-SINpEt)$_2$ **12** with Ru(ICy)$_2$ on a 0.1 mmol scale at 60 °C.

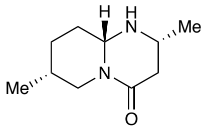

4a, white "oily solid". 83% yield, 56:44 dr, 96% ee, 2% ee. The product was isolated by column chromatography using Alumina N as stationary phase (pure EtOAc). Slow product decomposition took place in solution at room temperature over a period of weeks. The dr was determined from an isolated mixture of both diastereomers after column chromatography. The major diastereomer was isolated by preparative HPLC (XDB-C18 prepHT, 5 μm, 21.1 x 150 mm, MeOH/H_2O = 20:80, 15 mL/min) and characterized: ^{1}H NMR (300 MHz, CD_2Cl_2) δ 4.37 (dt, J = 13.3, 2.5 Hz, 1H), 4.03 (dd, J = 10.7, 2.8 Hz, 1H), 3.05–2.91 (m, 1H), 2.58 (dd, J = 13.4, 3.3 Hz, 1H), 2.37 (dd, J = 17.0, 3.5 Hz, 1H), 2.06–1.89 (m, 2H), 1.84–1.65 (m, 2H), 1.64–1.51 (m, 1H), 1.51–1.35 (m, 1H), 1.24–1.02 (m, 4H), 0.94 (d, J = 7.1 Hz, 3H); ^{13}C NMR (101 MHz, CD_2Cl_2) δ 168.67, 71.33, 47.03, 45.22, 42.08, 29.59, 28.90, 27.64, 21.94, 16.08. ESI-MS: calculated $[C_{10}H_{18}N_2O+H]^+$: 183.1492, found: 183.1495. Chir. GC (100_5_0.5_180_5_1_220_10): t_1 = 136.6 min (minor diastereomer, major enantiomer), t_2 = 140.2 min (major diastereomer, minor enantiomer), t_3 = 149.6 min (minor diastereomer, minor enantiomer), t_4 = 162.6 min (major diastereomer, major enantiomer).

Racemate:

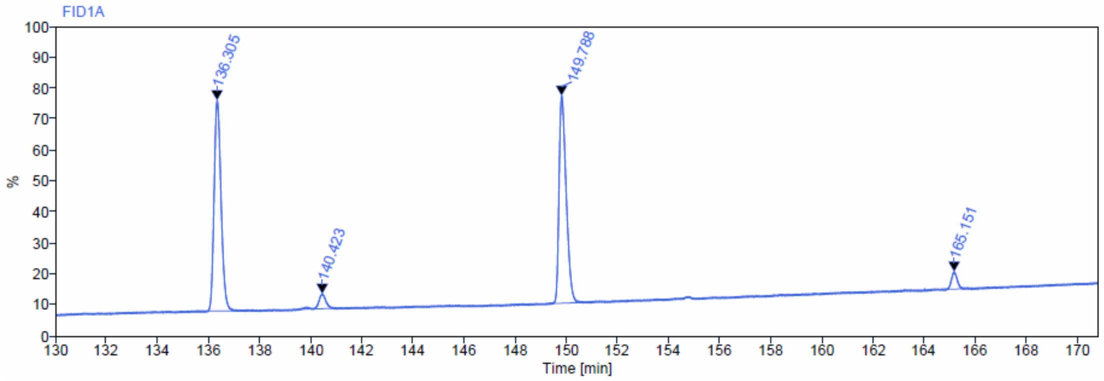

Signal:	FID1A	
RT [min]	Area	Area%
136.305	125.06	46.47
140.423	8.88	3.30
149.788	126.96	47.18
165.151	8.21	3.05
Sum	269.11	

Enantioenriched sample:

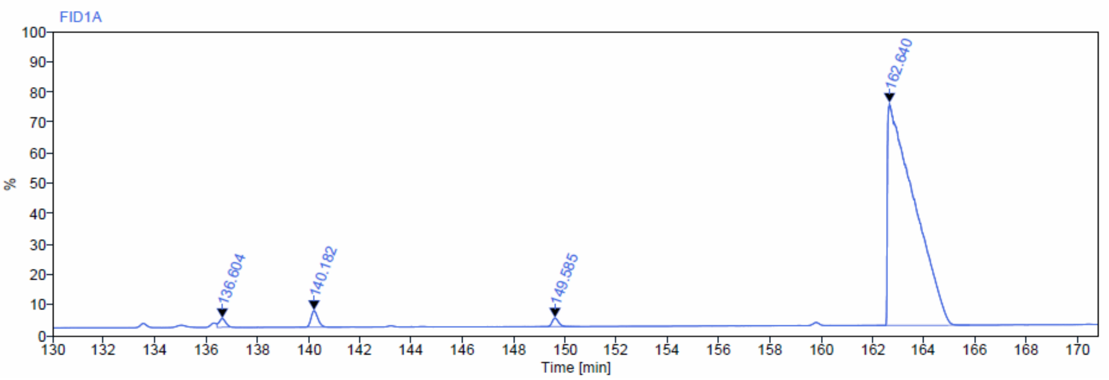

Signal:	FID1A	
RT [min]	Area	Area%
136.604	43.44	0.92
140.182	86.79	1.83
149.585	42.12	0.89
162.640	4573.15	96.37
Sum	4745.51	

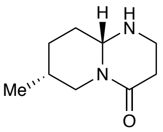

4b, colorless oil. 59% yield, 60:40 dr, 97% ee, 22% ee. The product was isolated by the acid-base workup, followed by column chromatography (CH_2Cl_2/MeOH = 95:5) and reverse phase preparative HPLC (H_2O/MeCN = 95:5 gradient to 5:95). The dr was determined by ^1H NMR spectroscopy. The signals of both diastereomers are listed: ^1H NMR (400 MHz, CD_2Cl_2) 4.59 (ddd, J = 13.2, 4.1, 2.5 Hz, 0.6H), 4.41 (dt, J = 13.3, 2.3 Hz, 0.4H), 4.09–3.93 (m, 1H), 3.18–3.07 (m, 1H), 2.97–2.81 (m, 1H),

2.59 (dd, J = 13.3, 3.4 Hz, 0.4H), 2.36–2.22 (m, 1.6H), 2.06–1.90 (m, 1.4H), 1.85–1.68 (m, 1.6H), 1.65–1.42 (m, 2H), 1.36–1.06 (m, 2H), 0.94 (d, J = 7.0 Hz, 1.2 Hz), 0.94 (d, J = 6.6 Hz, 1.8 Hz); ^{13}C NMR (101 MHz, CD_2Cl_2) δ 168.50, 167.66, 71.72, 71.32, 47.23, 45.48, 40.68, 40.63, 34.57, 34.42, 34.07, 32.78, 31.30, 29.77, 28.71, 27.71, 19.06, 16.07. ESI-MS: calculated $[C_9H_{16}N_2O+H]^+$: 169.1335, found: 169.1334. Chir. GC (100_5_0.5_180_5_1_220_10): t_1 = 127.3 min (minor diastereomer, major enantiomer), t_2 = 130.2 min (major diastereomer, minor enantiomer), t_3 = 136.0 min (minor diastereomer, minor enantiomer), t_4 = 139.4 min (major diastereomer, major enantiomer).

Racemate:

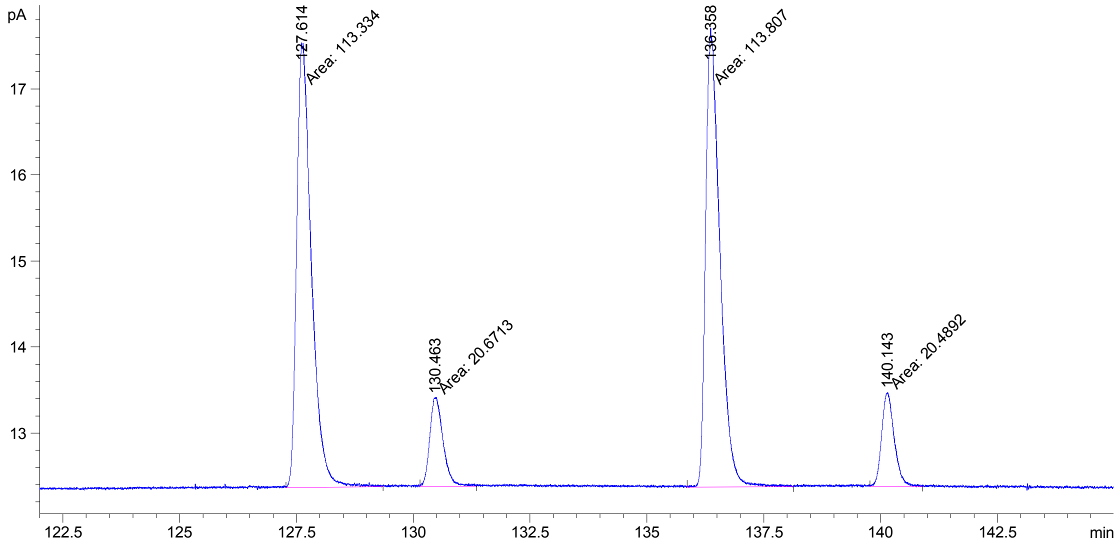

Peak #	RetTime [min]	Type	Width [min]	Area [pA*s]	Height [pA]	Area %
1	127.614	MM	0.3659	113.33426	5.16258	1.267e2
2	130.463	MM	0.3320	20.67130	1.03775	23.11349
3	136.358	MM	0.3557	113.80692	5.33258	1.273e2
4	140.143	MM	0.3128	20.48918	1.09170	22.90986

Enantioenriched sample:

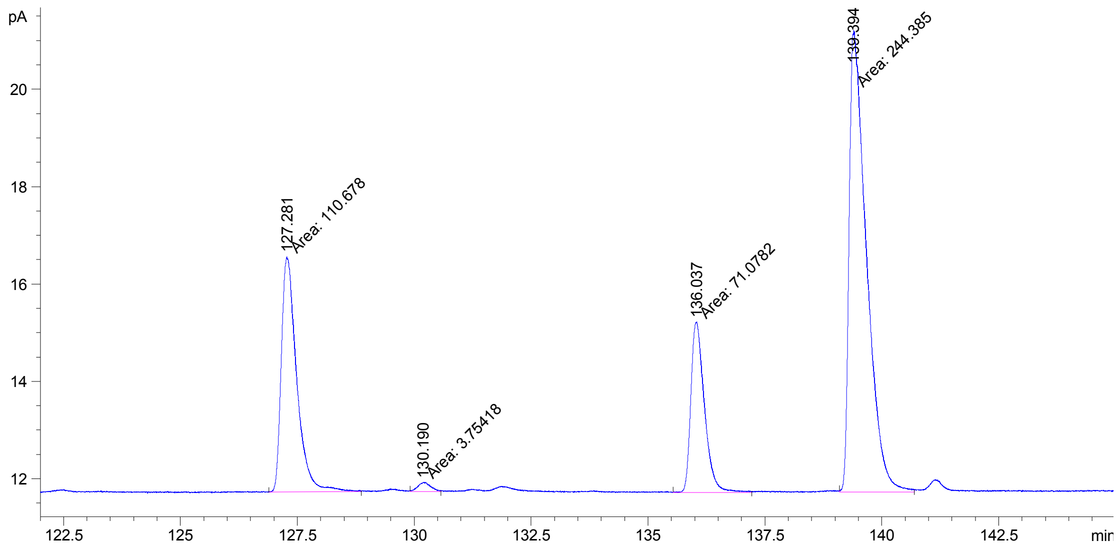

Peak #	RetTime [min]	Type	Width [min]	Area [pA*s]	Height [pA]	Area %
1	127.281	FM	0.3811	110.67764	4.84011	77.23574
2	130.190	MF	0.3208	3.75418	1.95056e-1	2.61983
3	136.037	MF	0.3371	71.07816	3.51382	49.60148
4	139.394	FM	0.4296	244.38542	9.48219	1.705e2

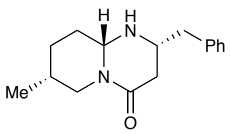

4c, white solid. 52% isolated yield (77% NMR yield vs mesitylene), 72:28 dr, 97% ee, 36% ee. The product was isolated by an acid-base workup followed by column chromatography (EtOAc). The dr was determined by ^{1}H NMR spectroscopy. The signals of both diastereomers are listed: ^{1}H NMR (300 MHz, CD_2Cl_2) δ 7.35–7.15 (m, 5H), 4.55 (ddd, *J* = 13.3, 4.1, 2.4 Hz, 0.3H), 4.36 (dt, *J* = 13.3, 2.4 Hz, 0.7H), 4.05–3.94 (m, 1H), 3.20–3.10 (m, 1H), 2.78–2.65 (m, 2H), 2.57 (d, *J* = 13.3, 3.3 Hz, 0.7H), 2.37–2.28 (m, 1H), 2.12–1.92 (m, 2.3H), 1.83–1.64 (m, 1.7H), 1.60–1.53 (m, 0.7 H), 1.51–1.34 (m, 1H), 1.29–1.08 (m, 1.6H), 0.96–0.88 (m, 3H); ^{13}C NMR (101 MHz, CD_2Cl_2) δ 168.51, 167.64, 138.39, 138.37, 129.74, 128.75, 126.79, 71.35, 70.90, 52.48, 52.42, 47.00, 45.29, 42.57, 39.97, 39.82, 34.27, 32.54, 31.29, 29.52, 28.84, 27.60, 19.07, 16.09; 4 carbon signals are missing due to peak overlap. ESI-MS: calculated $[C_{16}H_{22}N_2O+H]^+$: 259.1805, found: 259.1808. Chir. HPLC (AD-H, eluent: hexane/*i*PrOH = 97:3, flowrate: 1 mL/min,

detection: 210 nm): t_1 = 13.2 min (minor diastereomer, minor enantiomer), t_2 = 15.1 min (major diastereomer, minor enantiomer), t_3 = 17.4 min (major diastereomer, major enantiomer), t_4 = 20.7 min (minor diastereomer, major enantiomer).

Racemate:

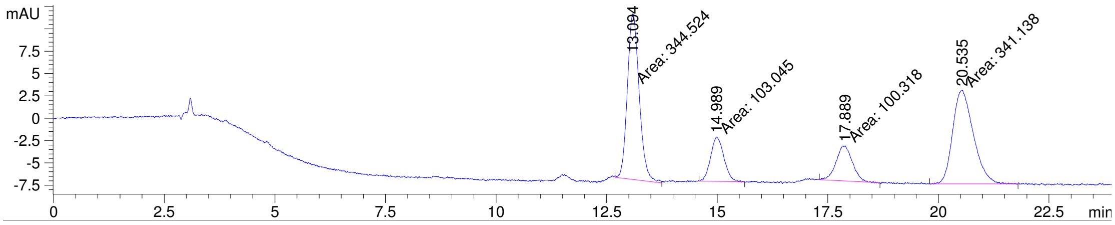

Peak #	RetTime [min]	Type	Width [min]	Area [mAU*s]	Height [mAU]	Area %
1	13.094	MM	0.3095	344.52368	18.55423	38.7529
2	14.989	MM	0.3468	103.04548	4.95247	11.5908
3	17.889	MM	0.4261	100.31837	3.92353	11.2841
4	20.535	MM	0.5441	341.13840	10.44968	38.3722

Enantioenriched sample:

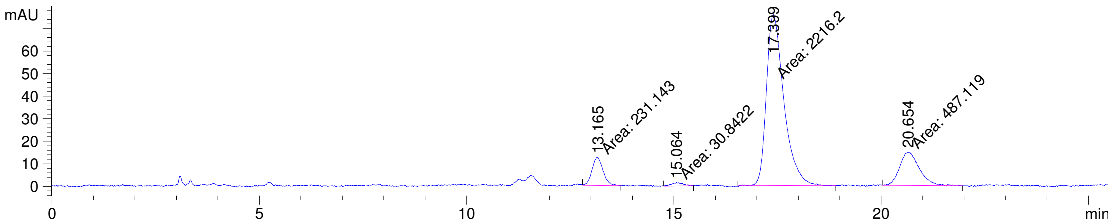

Peak #	RetTime [min]	Type	Width [min]	Area [mAU*s]	Height [mAU]	Area %
1	13.165	MM	0.3111	231.14291	12.38345	7.7949
2	15.064	MM	0.3266	30.84225	1.57393	1.0401
3	17.399	MM	0.4873	2216.20288	75.80170	74.7377
4	20.654	MM	0.5497	487.11862	14.76970	16.4273

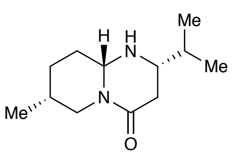

4d, off-white “oily solid”. 87% yield, 70:30 dr, 84% ee, 1% ee. The product was isolated by the acid-base workup. The dr was determined by ^{1}H NMR spectroscopy. The signals of both diastereomers are listed: ^{1}H NMR (300 MHz, CD_2Cl_2) δ 4.57 (ddd, J = 13.4, 4.2, 2.4 Hz, 0.3H), 4.37 (dt, J = 13.3, 2.4 Hz, 0.7H), 4.06–3.89 (m, 1H), 2.66–2.49 (m, 1.7H), 2.33–2.30 (m, 1H), 2.10–1.88 (m, 2.3H), 1.86–1.64 (m, 2H), 1.64–1.05 (m, 4H), 1.01–0.81 (m, 9H); only the signals of the major diastereomer are listed: ^{13}C NMR (75 MHz, CD_2Cl_2) δ 169.11, 71.51, 56.67, 45.25, 37.57, 33.12, 29.64, 28.89, 27.64, 18.97, 18.45, 16.09. Two signals are visible for the *iso*-propyl-derived methyl groups in the ^{13}C NMR spectrum. We assume that these methyl groups are not equivalent in the ^{13}C NMR spectrum due to a hindered rotation around the C_2–$CHMe_2$ bond. ESI-MS: calculated $[C_{12}H_{22}N_2O+H]^+$: 211.1805, found: 211.1808. Chir. HPLC (OD-H, eluent: hexane/*i*PrOH = 95:5, flowrate: 1 mL/min, detection: 210 nm): t_1 = 8.9 min (minor diastereomer, major enantiomer), t_2 = 10.5 min (minor diastereomer, minor enantiomer), t_3 = 12.7 min (major diastereomer, minor enantiomer), t_4 = 20.7 min (major diastereomer, major enantiomer).

Racemate:

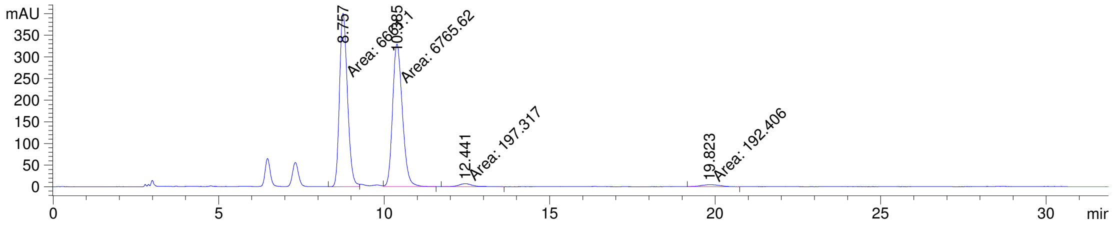

Peak #	RetTime [min]	Type	Width [min]	Area [mAU*s]	Height [mAU]	Area %
1	8.758	MF	0.2757	1475.08203	89.17328	48.0615
2	10.384	FM	0.3426	1508.33875	73.38745	49.1450
3	12.475	MF	0.4575	43.10317	1.57030	1.4044
4	19.798	MM	0.7303	42.63360	9.72911e-1	1.3891

Enantioenriched sample:

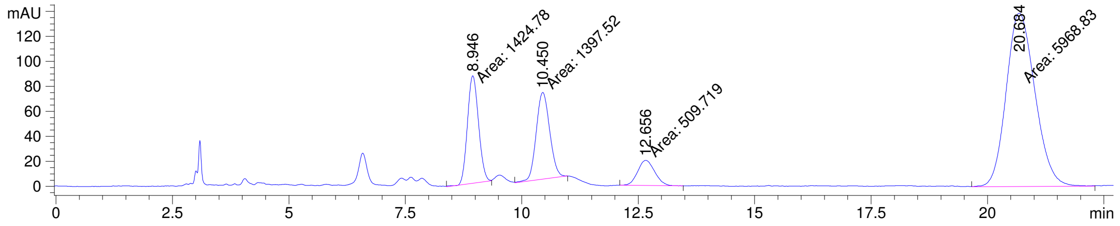

Peak #	RetTime [min]	Type	Width [min]	Area [mAU*s]	Height [mAU]	Area %
1	8.946	MM	0.2761	1424.78223	86.00065	15.3188
2	10.450	MM	0.3353	1397.52002	69.46313	15.0257
3	12.656	MM	0.4235	509.71881	20.06030	5.4803
4	20.684	MM	0.7173	5968.82959	138.69594	64.1751

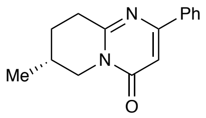

8e, off-white solid. 56% yield, 62% ee. The product was isolated by two sequential column chromatographies (pentane/EtOAc = 1:1 gradient to pure EtOAc). Traces of a small impurity could not be separated from the product. A mixture of a hexahydropyrido-pyrimidinone and 4 diastereomers of an octahydropyrido-pyrimidinone was obtained as byproduct. ^{1}H NMR (400 MHz, CD_2Cl_2) δ 8.00–7.94 (m, 2H), 7.48–7.38 (m, 3H), 6.69 (s, 1H), 4.32 (ddd, *J* = 14.4, 5.0, 1.9 Hz, 1H), 3.16 (dd, *J* = 14.4, 10.5 Hz, 1H), 3.08 (ddd, *J* = 18.2, 5.9, 3.4 Hz, 1H), 2.99–2.90 (m, 1H), 2.08–1.96 (m, 2H), 1.57–1.44 (m, 1H), 1.14 (d, *J* = 6.5 Hz, 3H); ^{13}C NMR (101 MHz, CD_2Cl_2) δ 163.24, 159.92, 159.68, 137.08, 130.57, 128.96, 127.20, 106.33, 49.22, 32.10, 28.57, 27.94, 19.08. ESI-MS: calculated $[C_{15}H_{16}N_2O+Na]^+$: 263.1155, found: 263.1165. Melting point: 105 °C. Chir. GC (100_5_0.5_180_5_1_220_10): t_1 = 141.0 min (minor enantiomer), t_2 = 143.0 min (major enantiomer).

Racemate:

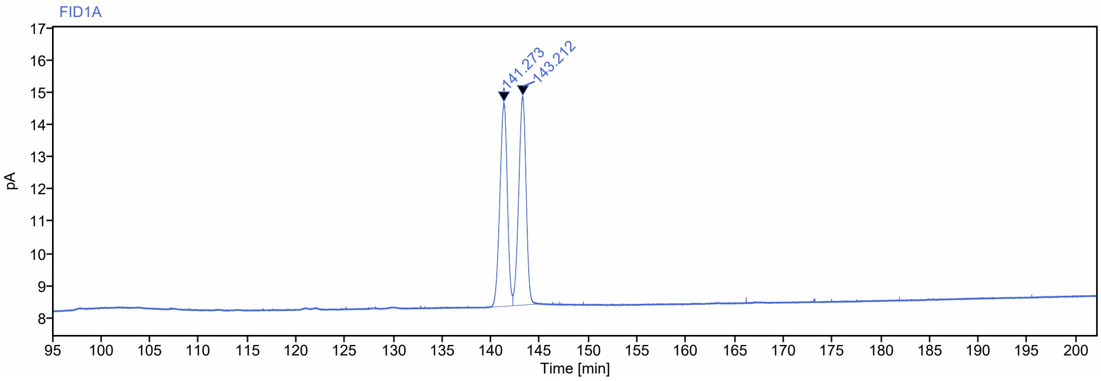

Signal:	FID1A				
RT [min]	Type	Width [min]	Area	Height	Area%
141.273	MM m	0.62	335.86	6.34	49.62
143.212	MM m	0.62	340.97	6.50	50.38
		Sum	676.83		

Enantioenriched sample:

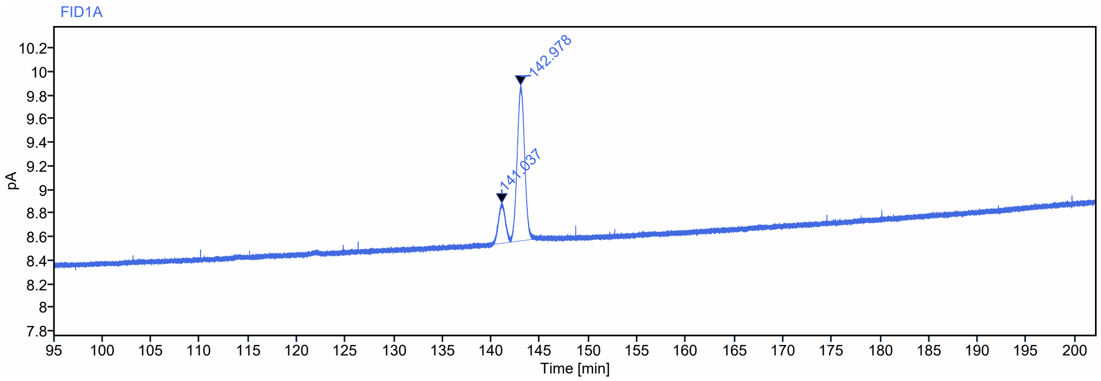

Signal:	FID1A				
RT [min]	Type	Width [min]	Area	Height	Area%
141.037	MM m	0.58	16.63	0.34	19.16
142.978	MM m	0.63	70.20	1.31	80.84
		Sum	86.83		

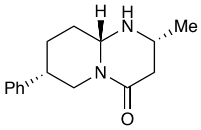

4f, off-white "oily solid". 74% yield, 42:58 dr, 96% ee, 22% ee. The crude product was isolated by the acid-base workup. A clean product was obtained by column chromatography using Alumina N as stationary phase (pentane/EtOAc = 1:1, gradient to pure EtOAc) after which the yield and dr were determined.

No separation of diastereomers was noted during chromatography. Measurement of the dr prior to chromatography was precluded by peak overlap with an unknown impurity. The signals of both diastereomers are listed: ^{1}H NMR (400 MHz, CD_2Cl_2) δ 7.36–7.13 (m, 5H), 5.09 (d, *J* = 14.1 Hz, 0.6H), 4.78 (dt, *J* = 13.2, 3.0 Hz, 0.4H), 4.20–4.12 (m, 1H), 3.14 (s, 0.6H), 3.09–2.97 (m, 1H), 2.88 (dd, *J* = 14.2, 4.0 Hz, 0.6H), 2.70–2.58 (m, 0.4 H), 2.53–2.35 (m, 1.4H), 2.20–2.11 (m, 1H), 2.10–1.90 (m, 2H), 1.82–1.69 (m, 1H), 1.46–1.16 (m, 2H), 1.15–1.07 (m, 3H); ^{13}C NMR (101 MHz, CD_2Cl_2) δ 168.33, 168.01, 143.44, 142.98, 128.83, 128.62, 127.88, 127.45, 127.03, 126.24, 71.53, 70.94, 47.18, 47.15, 46.33, 42.58, 42.37, 42.04, 37.03, 34.33, 31.16, 30.22, 28.98, 21.95; 2 carbon signals missing. ESI-MS: calculated $[C_{15}H_{20}N_2O+H]^+$: 245.1648, found: 245.1662. Chir. GC (100_5_0.5_180_5_1_220_30): t_1 = 218.3 min (major diastereomer, major enantiomer), t_2 = 219.8 min (major diastereomer, minor enantiomer), t_3 = 226.3 min (minor diastereomer, minor enantiomer), t_4 = 227.0 min (minor diastereomer, major enantiomer).

Racemate:

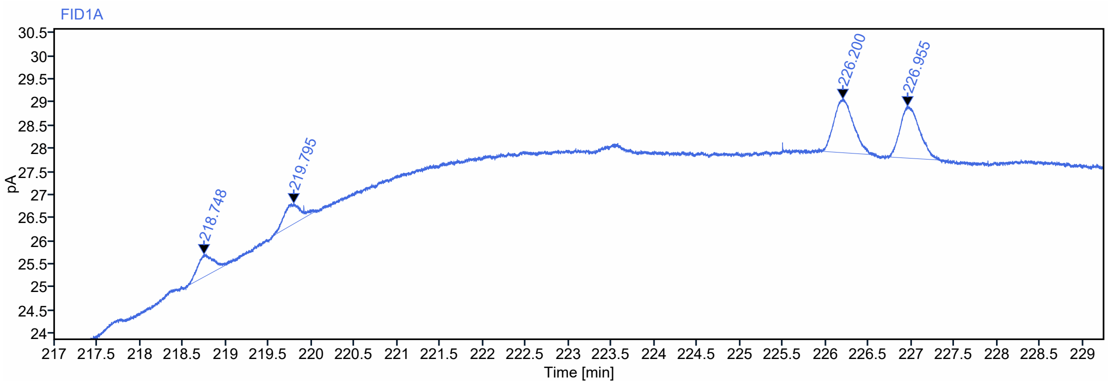

**Signal:**	FID1A	
**RT [min]**	**Area**	**Area%**
218.748	6.05	12.59
219.795	6.09	12.67
226.200	18.30	38.08
226.955	17.62	36.66
Sum	48.06	

Enantioenriched sample:

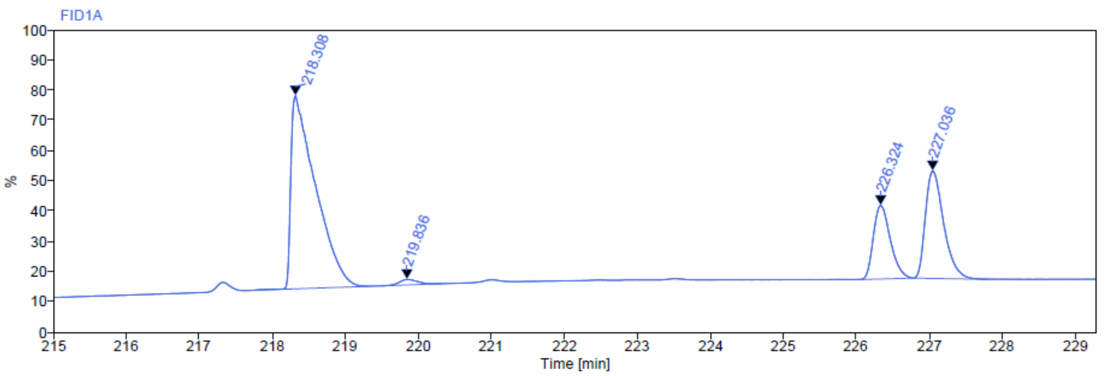

**Signal:**	FID1A	
**RT [min]**	**Area**	**Area%**
218.308	1667.82	58.97
219.836	34.54	1.22
226.324	437.54	15.47
227.036	688.41	24.34
Sum	2828.31	

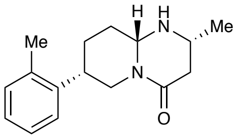

4g, white "oily solid". 81% yield, 23:77 dr, 94% ee, 27% ee. The crude product was isolated by the acid-base workup. A clean product was obtained by column chromatography (pure CH_2Cl_2 gradient to CH_2Cl_2/MeOH = 95:5). An additional set of signals is visible (e.g. signals at 4.31 and 3.34 ppm), which presumably results from a hindered rotation around the C7–*ortho*-tolyl bond. The signals of both diastereomers and the rotamer are listed: ^{1}H NMR (400 MHz, $CDCl_3$) δ 7.28–7.07 (m, 4H), 4.86 (dt, *J* = 14.2, 2.2 Hz, 0.2H), 4.79–4.66 (m, 0.8H), 4.35–4.11 (m, 1H), 3.38–3.29 (m, 0.15H), 3.26 (tt, *J* = 5.2, 2.5 Hz, 0.2H), 3.12–2.95 (m, 1.05H), 2.90–2.75 (m, 0.8H), 2.52–2.38 (m, 1.8H), 2.38–2.30 (m, 3H), 2.21–1.87 (m, 3H), 1.86–1.72 (m, 1H), 1.55 (bs, 1H), 1.47–1.28 (m, 1H), 1.18–1.12 (m, 3H); ^{13}C NMR (101 MHz, CD_2Cl_2) δ 168.45, 167.97, 166.91, 142.48, 141.39, 141.28, 136.52, 136.35, 136.07, 130.83, 127.40, 126.71, 126.68, 126.48, 126.44, 126.42, 126.16, 125.58, 125.44, 71.34, 71.02, 70.01, 47.32, 47.19, 47.12, 45.60, 44.43, 43.62, 42.17, 42.03, 41.26, 38.41, 38.36, 34.55, 33.84, 33.67, 31.52, 30.60, 28.90, 28.23, 22.00,

21.94, 20.99, 19.55, 19.52; 3 carbon signals missing. ESI-MS: calculated $[C_{16}H_{22}N_2O+H]^+$: 259.1805, found: 259.1808. Chir. GC (100_5_0.5_180_5_1_220_10): t_1 = 229.9 min (minor diastereomer, minor enantiomer), t_2 = 231.6 min (minor diastereomer, major enantiomer), t_3 = 233.1 min (major diastereomer, major enantiomer), t_4 = 234.9 min (major diastereomer, minor enantiomer).

Racemate:

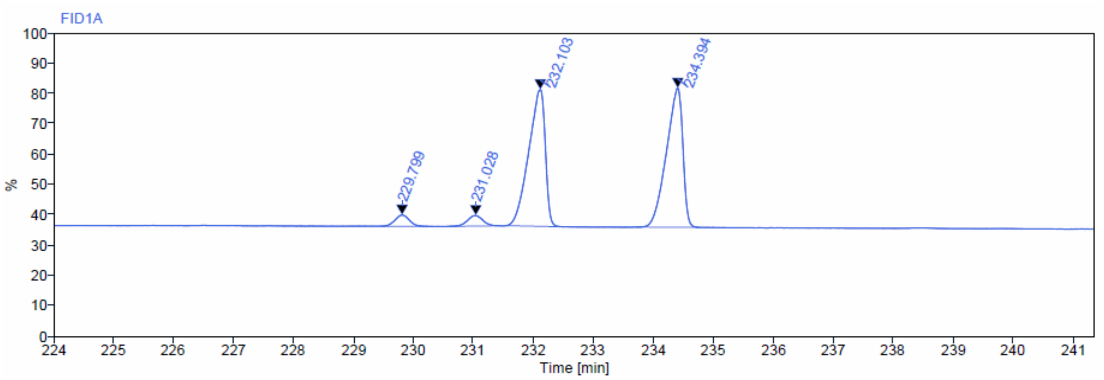

Signal: FID1A

RT [min]	Area	Area%
229.799	32.99	3.30
231.028	32.99	3.30
232.103	464.60	46.52
234.394	468.13	46.87
Sum	998.72	

Enantioenriched sample:

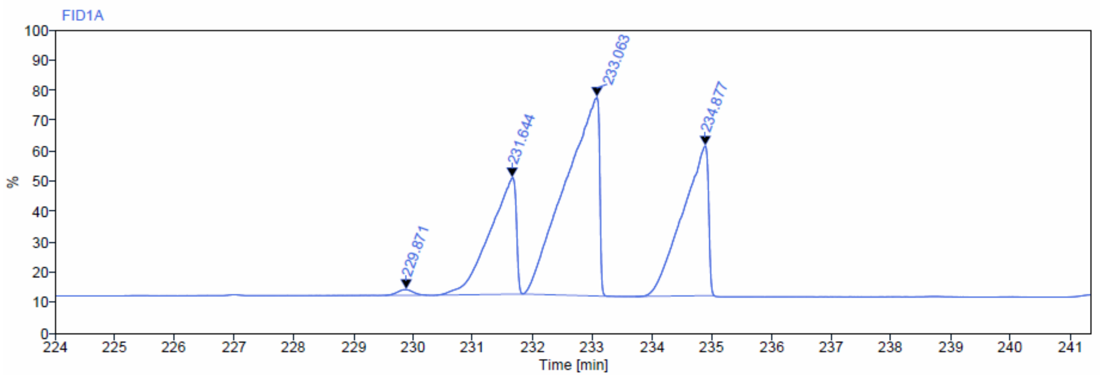

Signal: FID1A

RT [min]	Area	Area%
229.871	50.41	0.63
231.644	1772.82	22.29
233.063	3888.81	48.90
234.877	2240.57	28.17
Sum	7952.61	

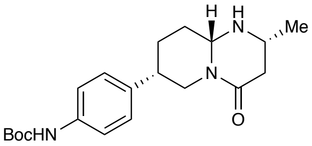

4h, white solid. 55% yield, 55:45 dr 92% ee, 24% ee. No acid-base workup was conducted. Instead, the crude product was purified by column chromatography (CH_2Cl_2/EtOAc = 1:1, gradient to CH_2Cl_2/EtOAc/MeOH = 50:45:5) after which the yield and dr were determined by NMR spectroscopy. No separation of diastereomers was observed during the column chromatography. Measurement prior to chromatography was precluded by peak overlap with an unknown impurity. Following protection with trifluoroacetic anhydride, the chiral samples were purified by preparative thin-layer chromatography prior to the measurement of the ee-values. The signals of both diastereomers are listed: ^{1}H NMR (400 MHz, CD_2Cl_2) δ 7.33–7.24 (m, 2H), 7.21 (d, J = 8.7 Hz, 0.9H), 7.16 (d, J = 8.6 Hz, 1.1H), 6.70 (s, 0.45H), 6.64 (s, 0.55H), 5.07 (dt, J = 14.1, 2.3 Hz, 0.45H), 4.74 (ddd, J = 13.2, 4.0, 2.4 Hz, 0.55H), 4.19–4.10 (m, 1H), 3.12–3.09 (m, 0.45H), 3.07–2.97 (m, 1H), 2.85 (dd, J = 14.1, 3.9 Hz, 0.45H), 2.59 (tt, J = 12.0, 3.7 Hz, 0.55H), 2.47–2.38 (m, 1.55H), 2.18–2.10 (m, 1H), 2.06–1.91 (m, 2H), 1.80–

1.69 (m, 1H), 1.52–1.45 (m, 9H), 1.43–1.32 (m, 1.1H), 1.25–1.16 (m, 0.9H), 1.14 (d, J = 6.4 Hz, 1.65H), 1.09 (d, J = 6.4 Hz, 1.35H); ^{13}C NMR (101 MHz, CD_2Cl_2) δ 168.39, 168.05, 153.14, 153.07, 137.88, 137.61, 137.26, 136.94, 128.32, 127.86, 118.94, 118.85, 80.54, 80.46, 71.54, 70.94, 47.16, 46.43, 42.47, 42.05, 42.01, 41.92, 36.48, 34.36, 31.21, 30.19, 28.96, 28.44, 21.96; 3 carbon signals missing. ESI-MS: calculated $[C_{20}H_{29}N_3O_3+H]^+$: 360.2282, found: 360.2291. Chir. HPLC (IA-3, eluent: hexane/*i*PrOH = 80:20, flowrate: 1 mL/min, detection: 210 nm): t_1 = 7.5 min (minor diastereomer, major enantiomer), t_2 = 9.0 min (major diastereomer, major enantiomer), t_3 = 9.8 min (minor diastereomer, minor enantiomer), t_4 = 10.4 (major diastereomer, minor enantiomer).

Racemate:

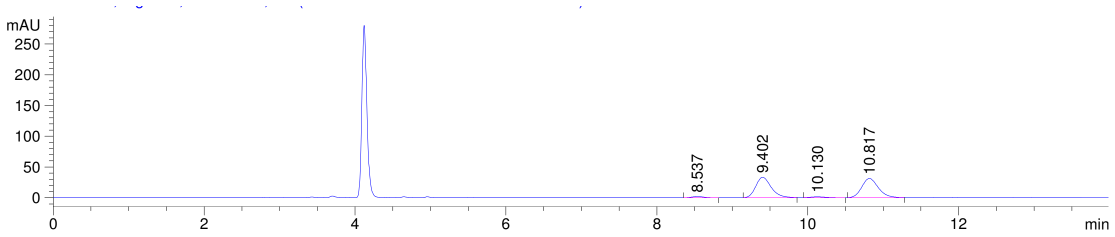

Peak #	RetTime [min]	Type	Width [min]	Area [mAU*s]	Height [mAU]	Area %
1	8.537	BB	0.1696	22.83673	2.02466	2.2708
2	9.402	BB	0.2186	479.67780	33.39067	47.6967
3	10.130	BB	0.2208	22.84248	1.47392	2.2713
4	10.817	BB	0.2353	480.32733	31.25471	47.7612

Enantioenriched sample:

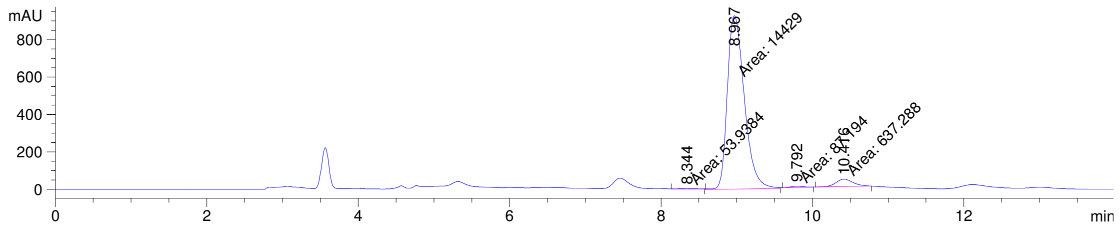

Peak #	RetTime [min]	Type	Width [min]	Area [mAU*s]	Height [mAU]	Area %
1	8.344	MM	0.2859	53.93837	3.14415	0.3547
2	8.967	MM	0.2598	1.44290e4	925.65918	94.8813
3	9.792	MM	0.2091	87.19405	6.94960	0.5734
4	10.416	MM	0.2662	637.28790	39.90319	4.1906

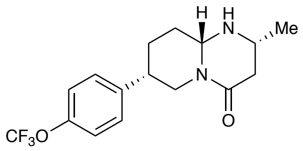

4i, colorless oil. 69% yield, 57:43 dr, 92% ee, 12% ee. The crude product was isolated by the acid-base workup. A clean product was obtained by two sequential column chromatographies (pure CH_2Cl_2 gradient to CH_2Cl_2/MeOH = 95:5). The dr was measured by ^{1}H NMR. The signals of both diastereomers are listed: ^{1}H NMR (400 MHz, CD_2Cl_2) δ 7.35 (d, 0.9H), 7.32–7.26 (m, 1.1H), 7.21–7.11 (m, 2H), 5.10 (dt, *J* = 14.2, 2.2 Hz, 0.4H), 4.78 (ddd, *J* = 13.3, 4.0, 2.5 Hz, 0.6H), 4.21–4.12 (m, 1H), 3.15 (s, 0.4H), 3.09–2.98 (m, 1H), 2.88 (dd, *J* = 14.3, 3.9 Hz, 0.4H), 2.68 (tt, *J* = 12.1, 3.7 Hz, 0.6H), 2.50–2.37 (m, 1.6H), 2.20–1.92 (m, 3H), 1.83–1.68 (m, 1.2H), 1.45–1.33 (m, 0.8H), 1.21–1.16 (m, 1H), 1.14 (d, *J* = 6.4 Hz, 1.8H), 1.10 (d, *J* = 6.4 Hz, 1.2H); ^{13}C NMR (101 MHz, CD_2Cl_2) δ 168.49, 168.12, 148.24 (m), 147.73 (m), 142.35, 141.87, 129.41, 128.90, 122.21 (d, *J* = 1.3 Hz), 121.41, 121.13, 119.67 (d, *J* = 1.7 Hz), 71.56, 70.92, 47.17, 46.21, 42.21, 42.04, 42.01, 36.64, 34.21, 31.17, 30.30, 28.90, 21.94, 21.92; 2 carbon signals missing; ^{19}F NMR (376 MHz, CD_2Cl_2) δ −58.26, −58.30. ESI-MS: calculated $[C_{16}H_{19}F_3N_2O_2+H]^+$: 329.1471, found: 329.1488. Chir. GC (100_5_0.5_180_5_1_220_10): t_1 = 212.0 min (major diastereomer, minor enantiomer), t_2 = 212.9 min (major diastereomer, major enantiomer), t_3 = 214.9 min (minor diastereomer, minor enantiomer), t_4 = 215.5 (minor diastereomer, major enantiomer).

Racemate:

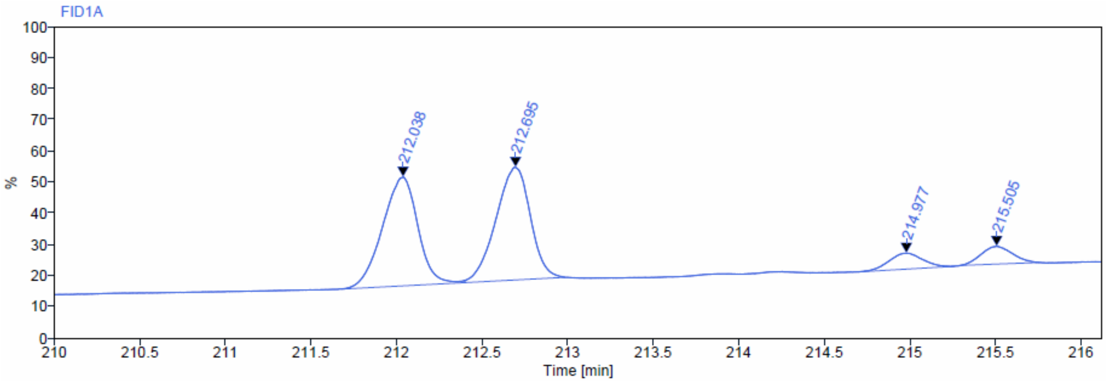

Signal: FID1A

RT [min]	Area	Area%
212.038	205.18	43.68
212.695	206.72	44.00
214.977	28.47	6.06
215.505	29.41	6.26
Sum	469.78	

Enantioenriched sample:

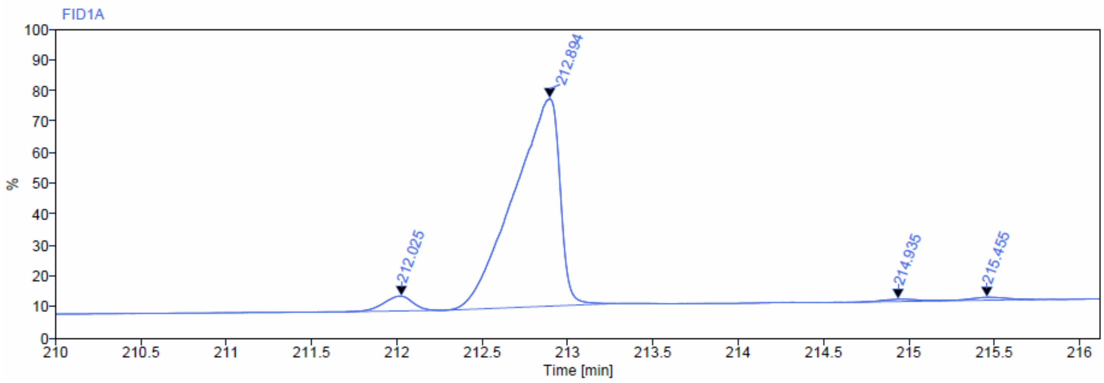

Signal: FID1A

RT [min]	Area	Area%
212.025	52.40	4.23
212.894	1161.86	93.89
214.935	10.15	0.82
215.455	13.03	1.05
Sum	1237.44	

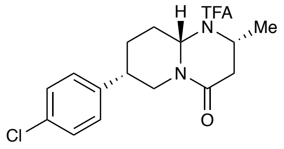

4j, colorless oil. 49% yield, 47:53 dr, 98% ee, 16% ee. The crude product was isolated by column chromatography (pentane/EtOAc = 9:1, gradient to pure EtOAc). Following protection with TFAA, a clean product could be obtained by a second column chromatography (pentane/EtOAc = 7:3 to pentane/EtOAc=3:7) after which the yield and dr were determined. Determination of the dr prior to the second chromatography was precluded by peak overlap with an unknown impurity. The signals of both diastereomers are listed: ^{1}H NMR (300 MHz, CD_2Cl_2) δ 7.35–7.15 (m, 4H), 5.45–5.25 (m, 1.5H), 4.90 (ddd, J = 13.0, 3.9, 2.5 Hz, 0.5H), 4.56–4.32 (m, 1H), 3.21–3.14 (m, 0.5H), 2.98 (ddd, J = 14.2, 3.7, 1.2 Hz, 0.5H), 2.83–2.51 (m, 2H), 2.50–2.36 (m, 1H), 2.33–2.23 (m, 1H), 2.23–2.05 (m, 1H), 2.03–1.65 (m, 1.5H), 1.53 (d, J = 6.9 Hz, 1.5H), 1.49–1.35 (m, 0.5H), 1.16 (d, J = 6.9 Hz, 1.5H); ^{13}C {^{19}F} NMR (126 MHz, CD_2Cl_2) δ 164.99, 164.11, 154.94, 154.73, 141.06, 140.41, 132.95, 132.34, 129.20, 129.10, 128.94, 128.90, 116.60, 116.52, 69.53, 68.94, 48.34, 48.13, 47.99, 44.50, 41.53, 38.46, 38.18, 35.99, 31.51, 31.35, 30.50, 26.67, 21.37, 20.56. ESI-MS: calculated $[C_{15}H_{19}ClN_2O+H]^+$: 279.1259, found: 279.1268. Chir. GC (100_10_190_5_200_0.5_220_30): t_1 = 78.7 min (minor diastereomer, minor enantiomer), t_2 = 80.2 min (minor diastereomer, major enantiomer), t_3 = 98.2 min (major diastereomer, minor enantiomer), t_4 = 99.3 min (major diastereomer, major enantiomer).

Racemate:

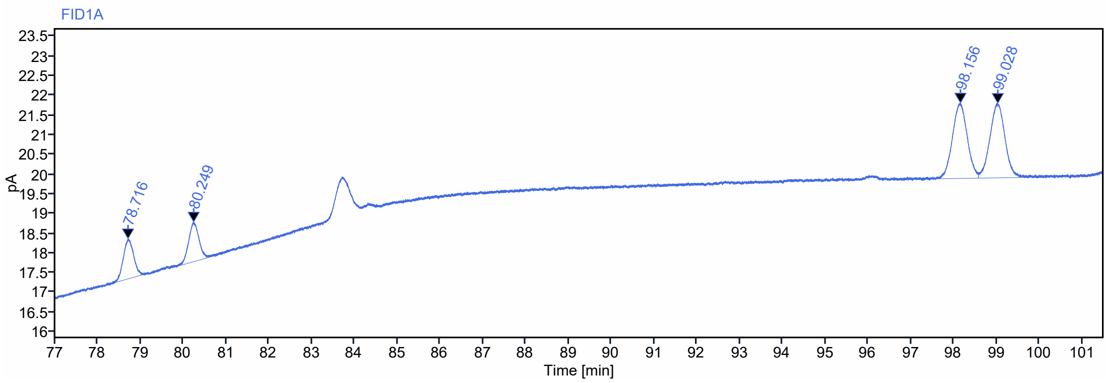

Signal:	FID1A	
RT [min]	Area	Area%
78.716	16.94	13.45
80.249	16.96	13.46
98.156	45.94	36.46
99.028	46.14	36.63
Sum	125.98	

Enantioenriched sample:

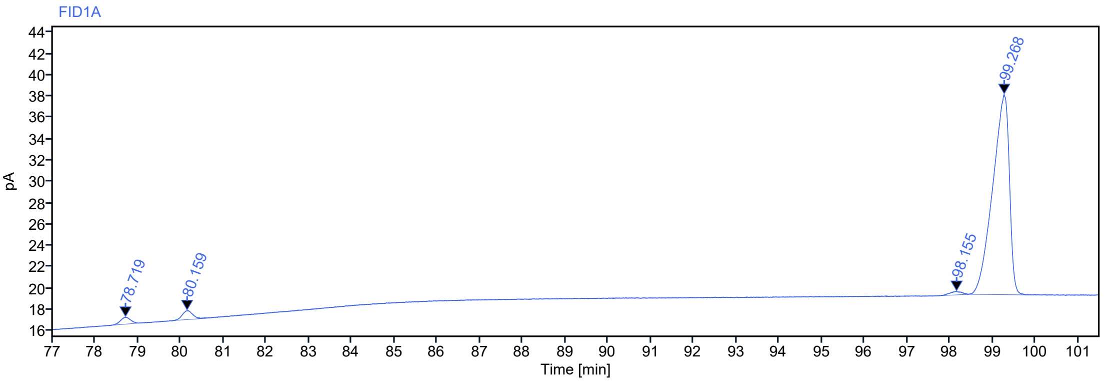

Signal:	FID1A	
RT [min]	Area	Area%
78.719	10.43	1.93
80.159	14.32	2.65
98.155	6.24	1.15
99.268	510.02	94.27
Sum	541.02	

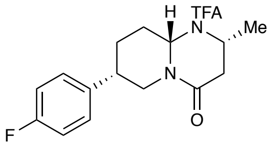

4k, pale-yellow oil. 76% yield, 42:58 dr, 69% ee, 24% ee. The crude product was isolated by the acid-base workup. Following protection with TFAA, a clean product was obtained by column chromatography (pentane/EtOAc = 9:1, gradient to pure EtOAc). The signals of both diastereomers are listed: ^{1}H NMR (300 MHz, $CDCl_3$) δ 7.41–7.18 (m, 2H), 7.11–6.97 (m, 2H), 5.46–5.22 (m, 1.6H), 4.95–4.61 (m, 0.4H), 4.57–4.45 (m, 0.4H), 4.45–4.33 (m, 0.6H), 3.19 (s, 0.6H), 3.00 (ddd, J = 14.2, 3.7, 1.2 Hz, 0.6H), 2.82–2.54 (m, 2H), 2.47 (dd, J = 6.3, 1.2 Hz, 0.6H), 2.41 (dd, J = 6.2, 1.4 Hz, 0.4H), 2.37–2.25 (m, 1H), 2.24–2.04 (m, 1H), 2.02–1.76 (m, 1.4H), 1.59–1.36 (m,

1.6H), 1.16 (d, J = 6.9 Hz, 1.8H); a third set of signals is visible in the ^{1}H NMR spectrum (e.g. signals at 4.66 and 1.40 ppm). We believe that these signals are a result of a restricted amide bond rotation of the TFA protecting group in the minor diastereomer. ^{13}C {^{19}F} NMR (126 MHz, CD_2Cl_2) δ 165.04, 164.15, 162.28, 162.12, 161.78, 161.74, 154.73, 138.31, 137.46, 129.27, 128.98, 115.73, 115.71, 115.55, 70.97, 69.54, 68.96, 50.98, 48.59, 48.41, 48.12, 47.98, 44.71, 41.41, 40.84, 38.46, 38.18, 35.82, 31.57, 31.51, 30.60, 26.60, 21.37, 20.47; 4 additional carbon signals observed. We believe these additional signals to be a result of a restricted amide bond rotation of the TFA protecting group in the minor diastereomer. ^{19}F NMR (470 MHz, CD_2Cl_2) δ −69.68, −69.82, −116.64, −116.73, −117.79; 1 additional ^{19}F signal observed. We believe this additional signal to be a result of a restricted amide bond rotation of the TFA protecting group in the minor diastereomer. ESI-MS: calculated $[C_{15}H_{19}FN_2O+Na]^+$: 285.1374, found: 285.1378. Chir. HPLC (OD-H, eluent: hexane/*i*PrOH = 95:5, flowrate: 1 mL/min, detection: 230 nm): t_1 = 23.1 min (major diastereomer, minor enantiomer), t_2 = 29.8 min (minor diastereomer, major enantiomer), t_3 = 32.3 min (major diastereomer, major enantiomer), t_4 = 53.3 (minor diastereomer, minor enantiomer).

Racemate:

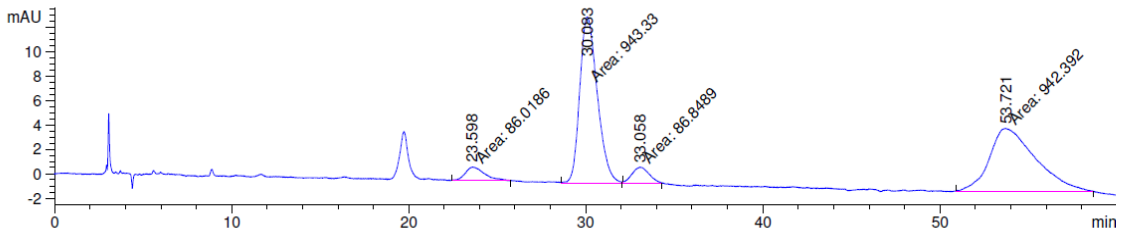

Peak #	RetTime [min]	Type	Width [min]	Area [mAU*s]	Height [mAU]	Area %
1	23.598	MM	1.2715	86.01859	1.12750	4.1785
2	30.083	MM	1.1524	943.32965	13.64239	45.8241
3	33.058	MM	1.0979	86.84890	1.31843	4.2189
4	53.721	MM	3.0349	942.39185	5.17528	45.7785

Enantioenriched sample:

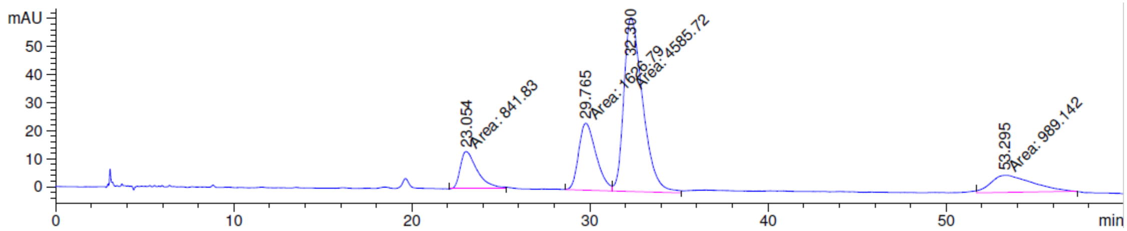

Peak #	RetTime [min]	Type	Width [min]	Area [mAU*s]	Height [mAU]	Area %
1	23.054	MM	1.0765	841.83044	13.03365	10.4660
2	29.765	MF	1.1337	1626.78918	23.91615	20.2249
3	32.300	FM	1.2323	4585.71680	62.02026	57.0116
4	53.295	MM	2.6847	989.14203	6.14052	12.2974

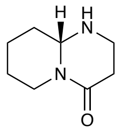

4l, brownish oil. 45% yield, 65% ee. The product was isolated by the acid-base workup. The product is somewhat volatile. ^{1}H NMR (300 MHz, $CDCl_3$) δ 4.74 (ddt, J = 13.6, 4.5, 2.3 Hz, 1H), 4.05 (dd, J = 10.8, 2.8 Hz, 1H), 3.16 (dt, J = 13.9, 4.8 Hz, 1H), 2.92 (ddd, J = 14.1, 8.9, 6.8 Hz, 1H), 2.47–2.26 (m, 3H), 2.03–1.61 (m, 4H), 1.52–1.15 (m, 3H); ^{13}C NMR (101 MHz, $CDCl_3$) δ 167.89, 71.45, 40.65, 40.44, 34.28, 33.93, 25.17, 23.98. ESI-MS: calculated $[C_8H_{14}N_2O+Na]^+$: 177.0998, found: 177.1006. Chir. GC (100_5_0.5_180_5_1_220_10): t_1 = 127.5 min (minor), t_2 = 133.7 min (major).

Racemate:

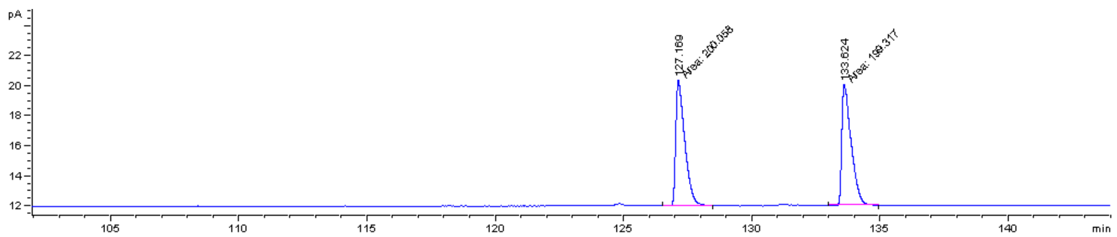

#	Time	Area	Height	Width	Symmetry
1	127.169	200.1	8.3	0.4017	0.408
2	133.624	199.3	8	0.4154	0.294

Enantioenriched sample:

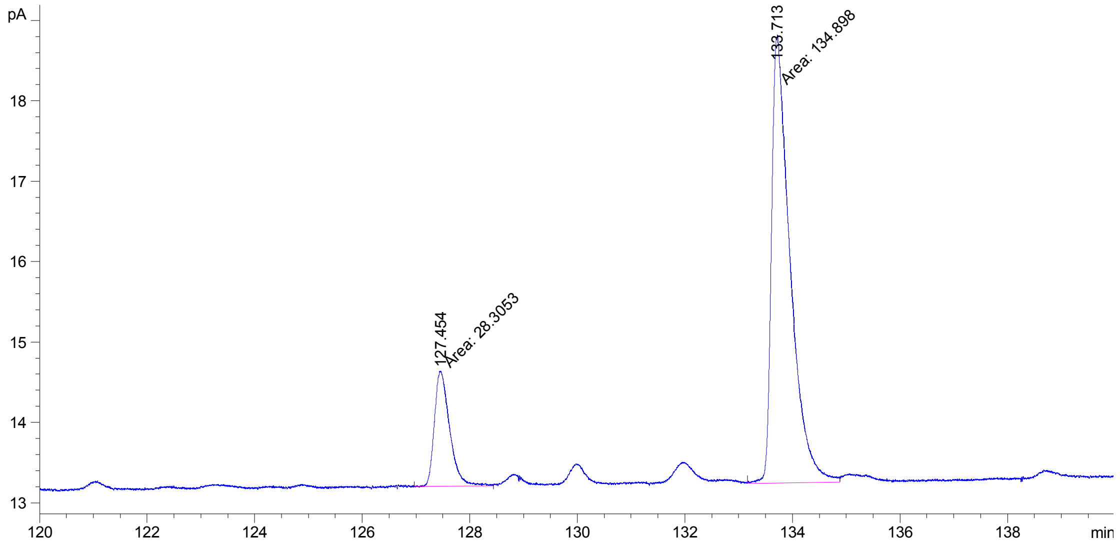

Peak #	RetTime [min]	Type	Width [min]	Area [pA*s]	Height [pA]	Area %
1	127.454	MM	0.3286	28.30526	1.43561	17.34360
2	133.713	MM	0.4034	134.89763	5.57355	82.65640

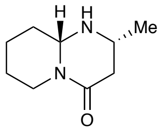

4m, 80 bar H_2 used. Off-white "oily solid". 76% yield, 91% ee. The product was isolated by the acid-base workup. ^{1}H NMR (400 MHz, CD_2Cl_2) δ 4.62 (ddt, *J* = 13.5, 4.4, 2.2 Hz, 1H), 4.02 (dd, *J* = 10.7, 2.9 Hz, 1H), 2.97 (dqd, *J* = 11.7, 6.4, 3.6 Hz, 1H), 2.42–2.27 (m, 2H), 2.01–1.89 (m, 2H), 1.86–1.79 (m, 1H), 1.71–1.64 (m, 1H), 1.50–1.23 (m, 3H), 1.21–1.13 (m, 1H), 1.09 (d, *J* = 6.4 Hz, 3H); ^{13}C NMR (101 MHz, CD_2Cl_2) δ 167.94, 71.28, 47.07, 42.04, 40.29, 34.24, 25.50, 24.10, 21.91. ESI-MS: calculated $[C_9H_{16}N_2O+Na]^+$: 191.1155, found: 191.1163. Chir. GC (100_5_0.5_180_5_1_220_10): t_1 = 118.0 min (minor), t_2 = 123.8 min (major).

Racemate:

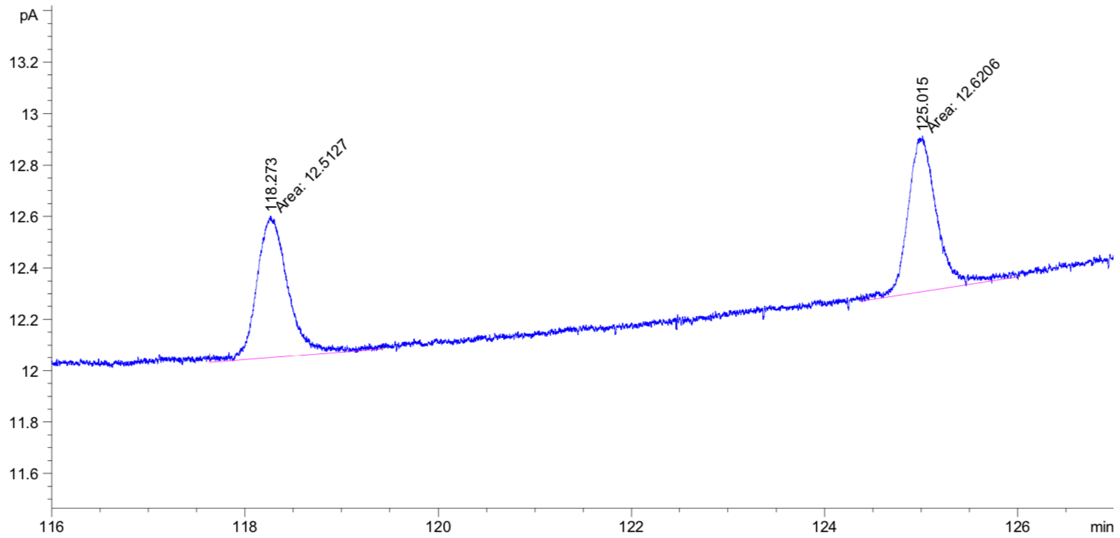

Peak #	RetTime [min]	Type	Width [min]	Area [pA*s]	Height [pA]	Area %
1	118.273	MM	0.3773	12.51267	5.52664e-1	49.78529
2	125.015	MM	0.3464	12.62060	6.07156e-1	50.21471

Enantioenriched sample:

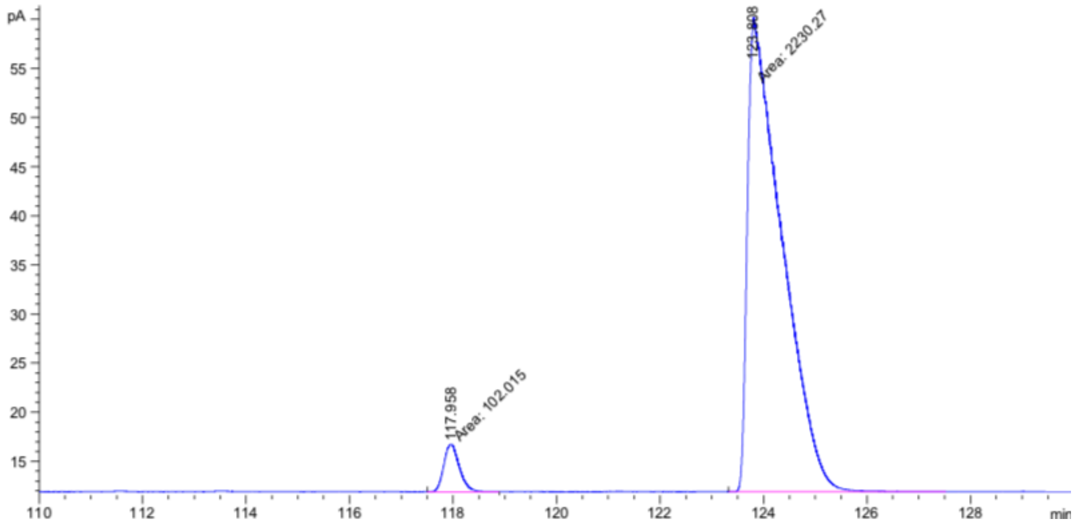

Peak #	RetTime [min]	Type	Width [min]	Area [pA*s]	Height [pA]	Area %
1	117.958	FM	0.3503	102.01468	4.85393	4.37402
2	123.808	FM	0.7690	2230.27246	48.33686	95.62598

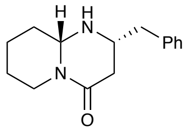

4n, white solid. 51% isolated yield (73% NMR yield), 86% ee. The product was isolated by the acid-base workup followed by column chromatography (EtOAc). ^{1}H NMR (400 MHz, CD_2Cl_2) δ 7.35–7.26 (m, 2H), 7.25–7.09 (m, 3H), 4.61 (ddt, J = 13.5, 4.4, 2.2 Hz, 1H), 4.00 (dd, J = 10.7, 2.8 Hz, 1H), 3.15 (dtd, J = 11.9, 6.5, 3.5 Hz, 1H), 2.78–2.64 (m, 2H), 2.41–2.27 (m, 2H), 2.04 (dd, J = 16.8, 11.9 Hz, 1H), 1.96 (dddd, J = 11.4, 4.9, 3.3, 1.8 Hz, 1H), 1.87–1.77 (m, 1H), 1.70–1.63 (m, 1H), 1.44 (qt, J = 13.0, 3.5 Hz, 1H), 1.36–1.12 (m, 3H); ^{13}C NMR (101 MHz, $CDCl_3$) δ 167.75, 138.34, 129.71, 128.71, 126.76, 71.23, 52.44, 42.55, 40.32, 39.89, 34.19, 25.44, 24.01. ESI-MS: calculated $[C_{15}H_{20}N_2O+H]^+$: 245.1648, found: 245.1648. Chir. HPLC (OD-H, eluent: hexane/*i*PrOH = 90:10, flowrate: 1 mL/min, detection: 210 nm): t_1 = 16.5 min (minor), t_2 = 19.3 min (major).

Racemate:

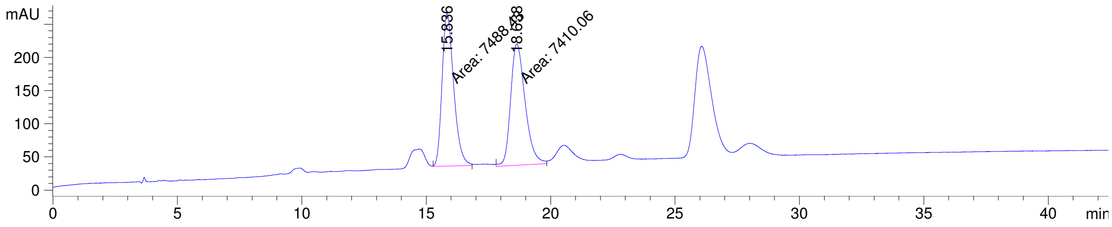

Peak #	RetTime [min]	Type	Width [min]	Area [mAU*s]	Height [mAU]	Area %
1	15.836	MM	0.5439	7488.42578	229.46747	50.2630
2	18.638	MM	0.6749	7410.06396	182.98271	49.7370

Enantioenriched sample:

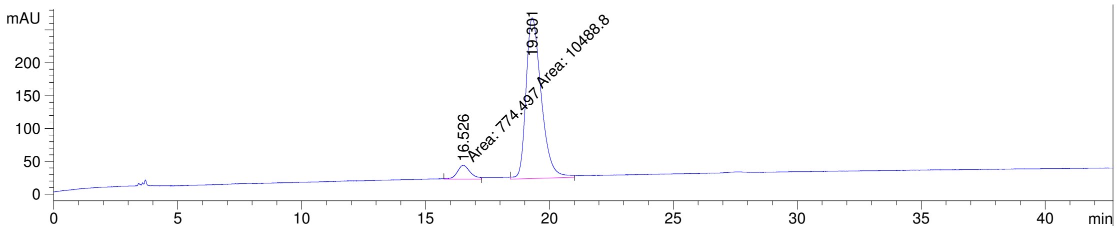

Peak #	RetTime [min]	Type	Width [min]	Area [mAU*s]	Height [mAU]	Area %
1	16.526	MM	0.6138	774.49670	21.03082	6.8763
2	19.301	MM	0.7149	1.04888e4	244.51611	93.1237

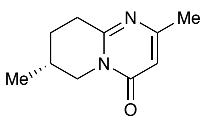

8a, 1.5 h reaction time, off-white solid. 84% yield, 63% ee. The product was isolated by column chromatography (pentane/EtOAc = 7:3 to pure EtOAc). ^{1}H NMR (300 MHz, $CDCl_3$) δ 9.15 (bs, 1H, NH presumably resulting from protonation of the basic nitrogen with trace HCl in $CDCl_3$), 6.22 (s, 1H), 4.32 (ddd, J = 14.6, 5.0, 2.0 Hz, 1H), 3.38–2.94 (m, 3H), 2.34 (d, J = 0.9 Hz, 3H), 2.15–1.93 (m, 2H), 1.52 (dddd, J = 14.1, 11.7, 10.7, 5.9 Hz, 1H), 1.16 (d, J = 6.4 Hz, 3H); ^{13}C NMR (75 MHz, $CDCl_3$) δ 160.99, 160.48, 158.25, 110.21, 49.34, 29.13, 27.81, 26.40, 21.07, 18.73. ESI-MS: calculated $[C_{10}H_{14}N_2O+H]^+$: 179.1179, found: 179.1179. Chir. HPLC (AS-H, eluent: hexane/*i*PrOH = 60:40, flowrate: 1 mL/min, detection: 360 nm): t_1 = 17.2 min (major), t_2 = 23.8 min (minor).

Racemate:

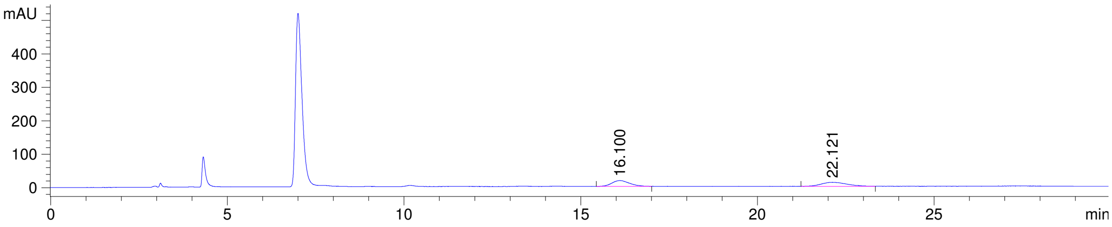

Peak #	RetTime [min]	Type	Width [min]	Area [mAU*s]	Height [mAU]	Area %
1	16.100	VV	0.4252	631.33722	17.57150	49.8354
2	22.121	VV	0.6084	635.50787	12.28760	50.1646

Enantioenriched sample:

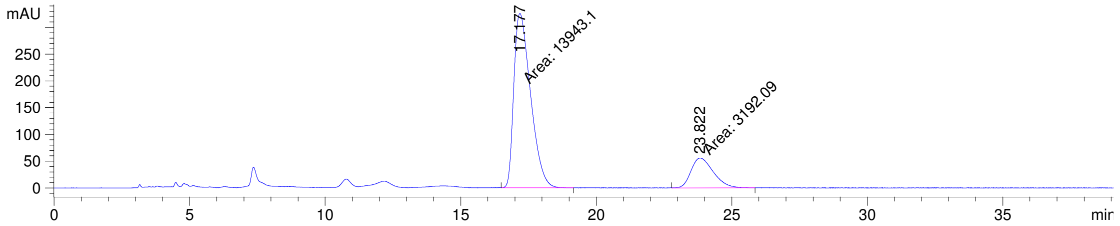

Peak #	RetTime [min]	Type	Width [min]	Area [mAU*s]	Height [mAU]	Area %
1	17.177	MM	0.7138	1.39431e4	325.58139	81.3712
2	23.822	MM	0.9546	3192.08765	55.73269	18.6288

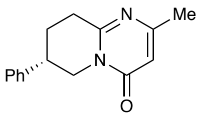

8f, 1.5 h reaction time, white solid. 50% yield, 62% ee. The product was isolated by column chromatography (pentane/EtOAc = 7:3 to pure EtOAc). ^{1}H NMR (400 MHz, $CDCl_3$) δ 7.39–7.34 (m, 2H), 7.32–7.24 (m, 3H), 6.20 (s, 1H), 4.54 (ddd, J = 14.6, 5.3, 1.8 Hz, 1H), 3.57 (dd, J = 14.6, 11.1 Hz, 1H), 3.18–2.96 (m, 3H), 2.26 (s, 3H), 2.25–2.21 (m, 1H), 2.08 (dtd, J = 13.4, 11.0, 6.0 Hz, 1H); ^{13}C NMR (126 MHz, CD_2Cl_2) δ 162.50, 158.48, 141.14, 129.08, 127.63, 127.11, 109.86, 48.44, 39.35, 31.81, 26.41, 23.66; 1 C missing. ESI-MS: calculated $[C_{15}H_{16}N_2O+H]^+$: 241.1335, found: 241.1347. Melting point: 125 °C. Chir. HPLC (AD-H, eluent: hexane/*i*PrOH = 90:10, flowrate: 1 mL/min, detection: 254 nm): t_1 = 19.2 min (major enantiomer), t_2 = 21.7 min (minor enantiomer).

Racemate:

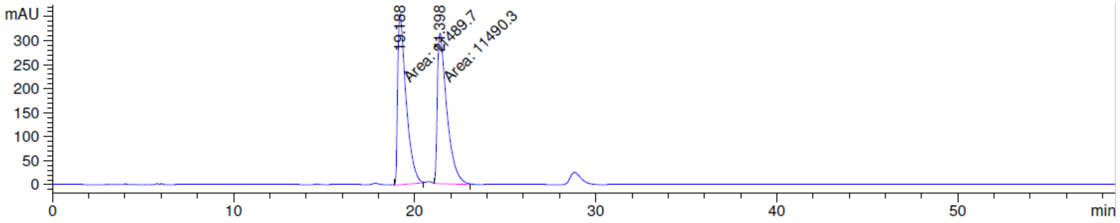

Peak #	RetTime [min]	Type	Width [min]	Area [mAU*s]	Height [mAU]	Area %
1	19.188	MM	0.5378	1.14897e4	356.05997	49.9988
2	21.398	MM	0.6112	1.14903e4	313.33002	50.0012

Enantioenriched sample:

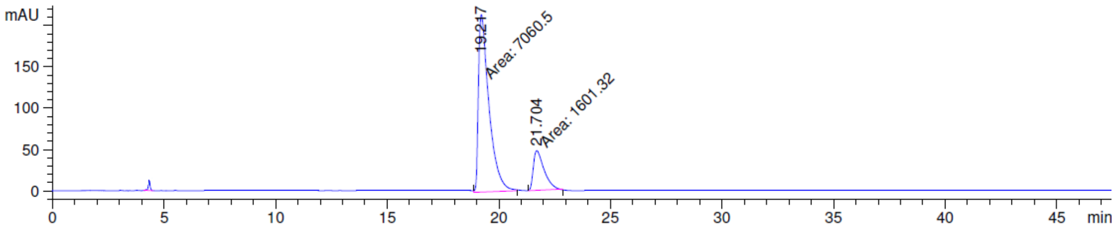

Peak #	RetTime [min]	Type	Width [min]	Area [mAU*s]	Height [mAU]	Area %
1	19.217	MM	0.5485	7060.50342	214.52309	80.7807
2	21.704	MM	0.5570	1601.31531	47.91694	18.3210

(G) Monitoring of the reaction progress

The reaction progress was monitored in order to verify that the tetrahydropyrido-pyrimidinones **8**, which were sometimes observed as side products of the reaction, are intermediates in the complete hydrogenation to the desired octahydropyrido-pyrimidinone products **4**.

Procedure

The progress of the reaction was monitored by reacting substrate **3a** (52.3 mg, 0.3 mmol) according to general procedure 1 for the indicated reaction time. Individual autoclaves were set up for each time point as the required high hydrogen pressure rendered in situ analysis impossible. Following the acid-base workup described in general procedure 1, the ratios of starting material **3a**, tetrahydropyrido-pyrimidinone **8a**, and octahydropyrido-pyridopyrimidinone **4a** were determined by ^{1}H NMR spectroscopy using the characteristic signals of the alkenyl protons of the substrate **3a** (6.3 ppm) and the tetrahydropyrido-pyrimidinone **8a** (6.2 ppm), and one of the characteristic diastereotopic H7-protons of the two diasteromers of octahydropyrido-pyrimidinone **4a** (4.4 ppm). After direct measurement of the ee of **8a**, CH_2Cl_2 (3 mL) was added. A sample (1 mL) was taken, protected with TFAA, and analyzed according to general procedure 1 to measure the ee values by chiral GC analysis.

Results

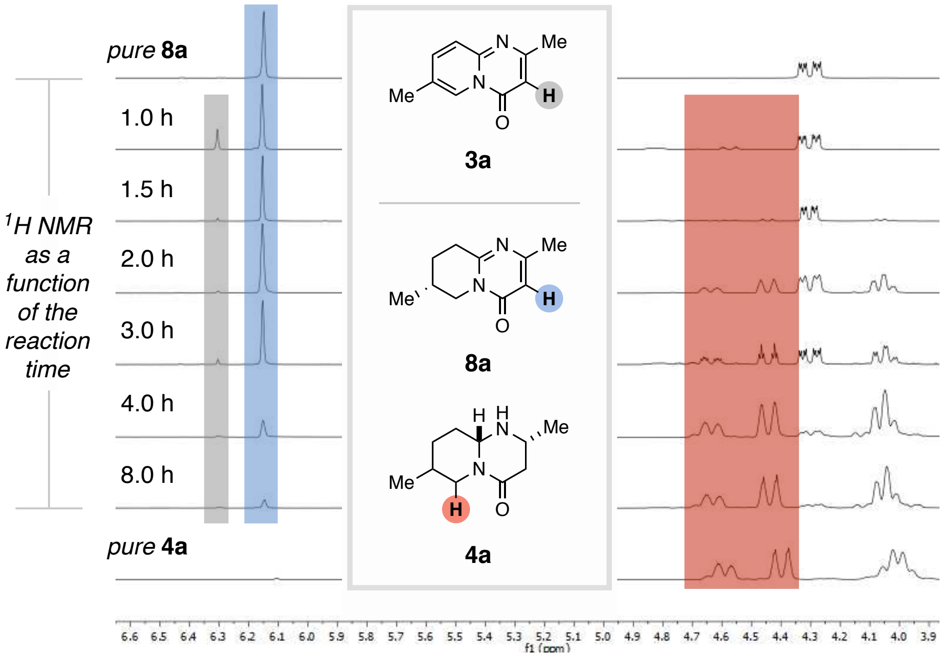

Figure S1. Excerpt of the ^{1}H NMR spectra of the isolated mixtures of substrate **3a**, tetrahydropyrido-pyrimidinone **8a** and octahydropyrido-pyrimidinone **4a** obtained by hydrogenation of **3a** at various time points. Clean **8a** is depicted in the top line and clean **4a** is depicted in the bottom line of the stacked spectra. All spectra containing significant amounts of tetrahydropyrido-pyrimidinone were aligned using their characteristic signal at 6.22 ppm as reference point to enable a convenient graphical overview of the reaction outcome.

Table S3. Ratio of substrate **3a**, tetrahydropyrido-pyrimidinone **8a**, and octahydropyrido-pyrimidinone **4a** and their dr and ee values as a function of the reaction time.

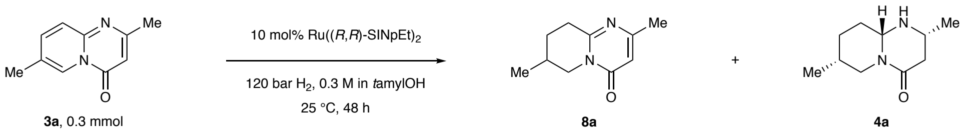

Time [h]	Ratio **3a** in [%]	Ratio **8a** (ee) both in [%]	Ratio **4a** in [%] (dr, ee **major** /ee minor in [%])
1	19	81 (58)	0 (-)
1.5	4	96 (63)	0 (-)
2	1	52 (78)	47 (88:12, **98**/64)
3	2	43 (80)	55 (85:15, **96**/70)
4	1	6 (86)	93 (86:14, **96**/50)
8	0	5 (92)	95 (79:21, **98**/40)
14	0	0 (-)	100 (80:20, **96**/36)

The reactions were conducted at a 0.3 mmol scale using 10 mol% Ru((*R,R*)-SINpEt)$_2$ under 120 bar H_2 pressure 0.3 M in *t*amylOH at 25 °C for 48 h.

It was observed that the ee value of the sample taken after 2 h was outside of the expected range. Hence, the corresponding experiment was repeated three times using an identical procedure. The three samples were combined and the ee of the tetrahydro-pyrimidinone **8a** was remeasured and determined to be 72%. This ee was used for figure S2 (vide infra) and figure 3 in the manuscript).

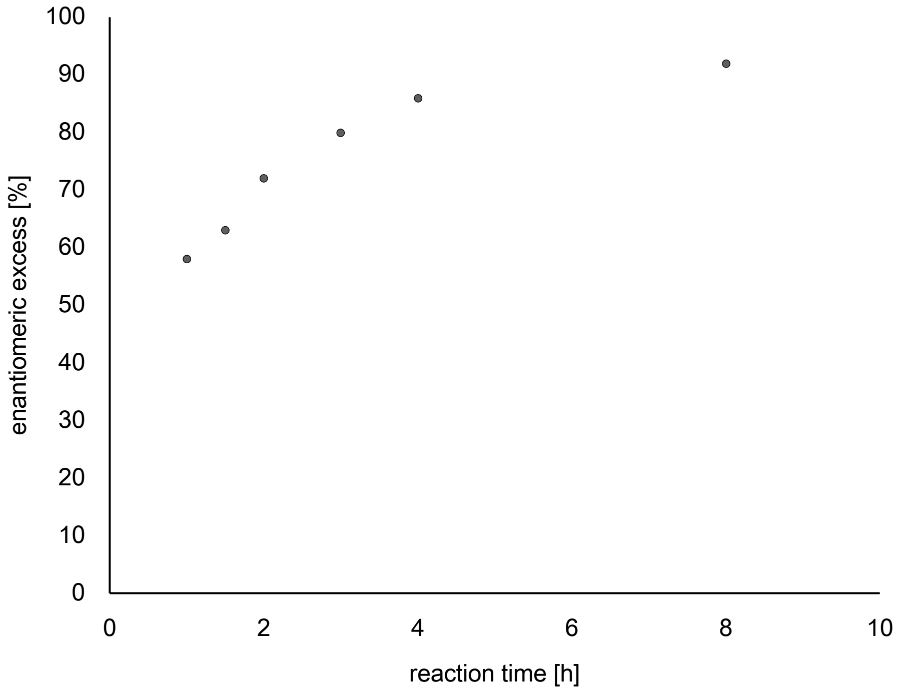

Figure S2. Enantiomeric excess of intermediate **8a** as a function of the reaction time.

Discussion

The relative abundance of the tetrahydropyrido-pyrimidinone **8a** increases until it becomes the almost exclusive species after ca. 1.5 h reaction time (Figure S1, Table S3). Then, its abundance starts to decrease with the abundance of octahydropyrido-pyrimidinone **4a** increasing at the same time until the reaction is completed after about 8 h. The ee of the tetrahydropyrido-pyrimidinone **8a** increases with the reaction time (Table S3, Figure S2). When stopping the reaction after 1.5 h, intermediate **8a** can be isolated in high yield and enantiomeric excess (84% yield, 63% ee) by standard column chromatography (see page S40).

The obtained results suggest that the tetrahydropyrido-pyrimidinones **8** are indeed intermediates in the complete hydrogenation towards the octahydropyrido-pyrimidinones **4**. Furthermore, the increase of the ee of the tetrahydropyrido-pyrimidinone **8a** with the reaction time – i. e. with an increased conversion to the octahydropyrido-pyrimidinone **4a** – indicates that the minor enantiomer reacts faster in the second hydrogenation step. One can conclude that a mismatched interaction between catalyst and substrate control must be present in this step. In accordance with this explanation, it is understandable that the measured diastereomeric ratio is

lower for substrates bearing large aryl-substituents in the 7-position (see products **4f–k**). Furthermore, it is noteworthy that the minor diastereomer in the racemic hydrogenation using $Ru(ICy)_2$ generally corresponds to the major diastereomer of the product obtained with chiral Ru((*R*,*R*)-SINpEt)$_2$ thus indicating that the chiral catalyst is able to somewhat overwrite the substrate control in the second hydrogenation step.

(H) Sequential hydrogenation

To test the mechanistic analysis outlined in section G, a sequential hydrogenation of pyrido-pyrimidinone **3a** was performed using opposite enantiomers of the chiral catalyst for the two distinct hydrogenation steps.

Procedure

6 vials were filled with the pyrido-pyrimidinone substrate **3a** (0.3 mmol, each), and reacted according to general procedure 1 using Ru((*R*,*R*))-SINpEt)$_2$ as catalyst for 2 h. The combined crude product was purified by column chromatography (pentane/EtOAc = 7:3 to pure EtOAc) and the ee was measured by chiral HPLC.

The generated tetrahydropyrido-pyrimidinone **8a** (0.3 mmol) was reacted according to general procedure 1 using either Ru((*R*,*R*))-SINpEt)$_2$ or Ru((*S*,*S*))-SINpEt)$_2$ as catalyst in two individual experiments. In both cases, the crude products were isolated by an acid-base workup before dr and ee were measured by chiral GC analysis.

Results

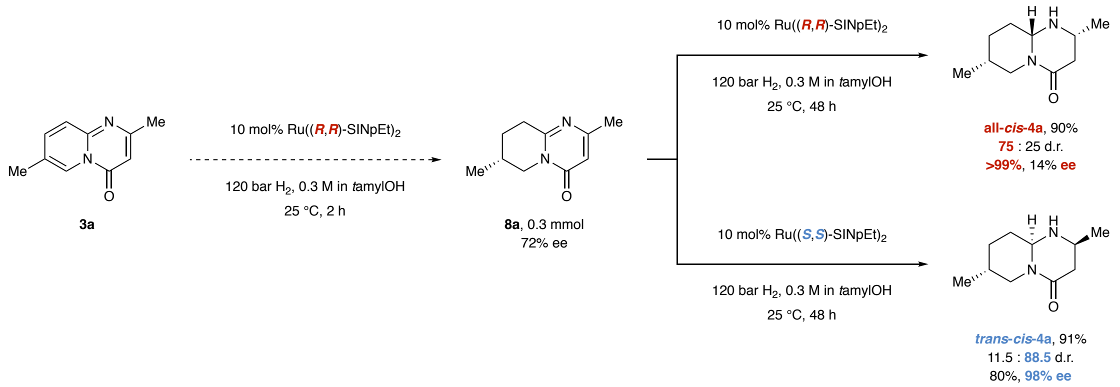

Figure S3. Sequential hydrogenation of substrate **3a** via isolated intermediate **8a** using identical (top equation) or opposite enantiomers (bottom equation) of the chiral catalyst **12**.

Discussion

When conducting the sequential hydrogenation using identical enantiomers of the chiral catalyst (top equation), the same diastereomer was obtained as major diastereomer compared to the standard one-pot reaction albeit with a slightly improved dr value. The higher dr value is a result of the higher ee value of the isolated tetrahydropyrido-pyrimidinone **8a** compared to its initial ee in the one-pot reaction since its ee increases as a function of the reaction time. In this regard, it is worth noting that intermediate **8a** was synthesized with a reaction time of 2 hours. Hence, it has a higher enantiomeric excess than the previously isolated sample (1.5 h reaction time). When conducting the two distinct hydrogenation steps with opposite enantiomers of the chiral catalyst **12**, the other diastereomer was obtained as major diastereomer with an excellent dr and ee.

This strongly supports the assumed mismatched interaction between chiral catalyst **12** and the chiral tetrahydropyrido-pyrimidinone **8a**, which is the substrate in the second distinct hydrogenation step.

(I) NMR spectra of new substrates

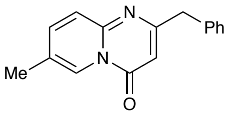

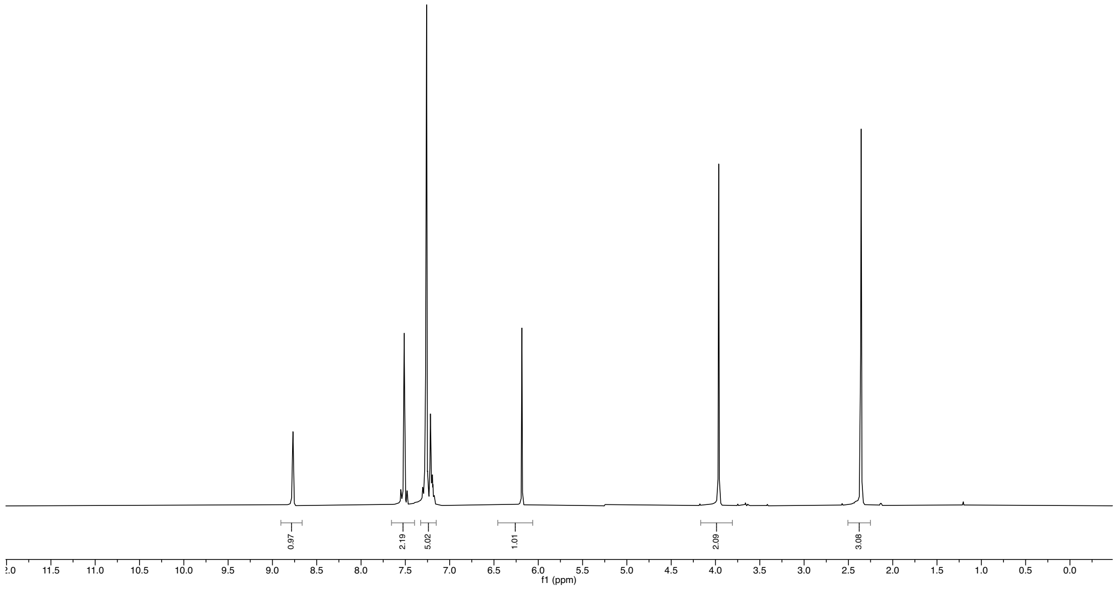

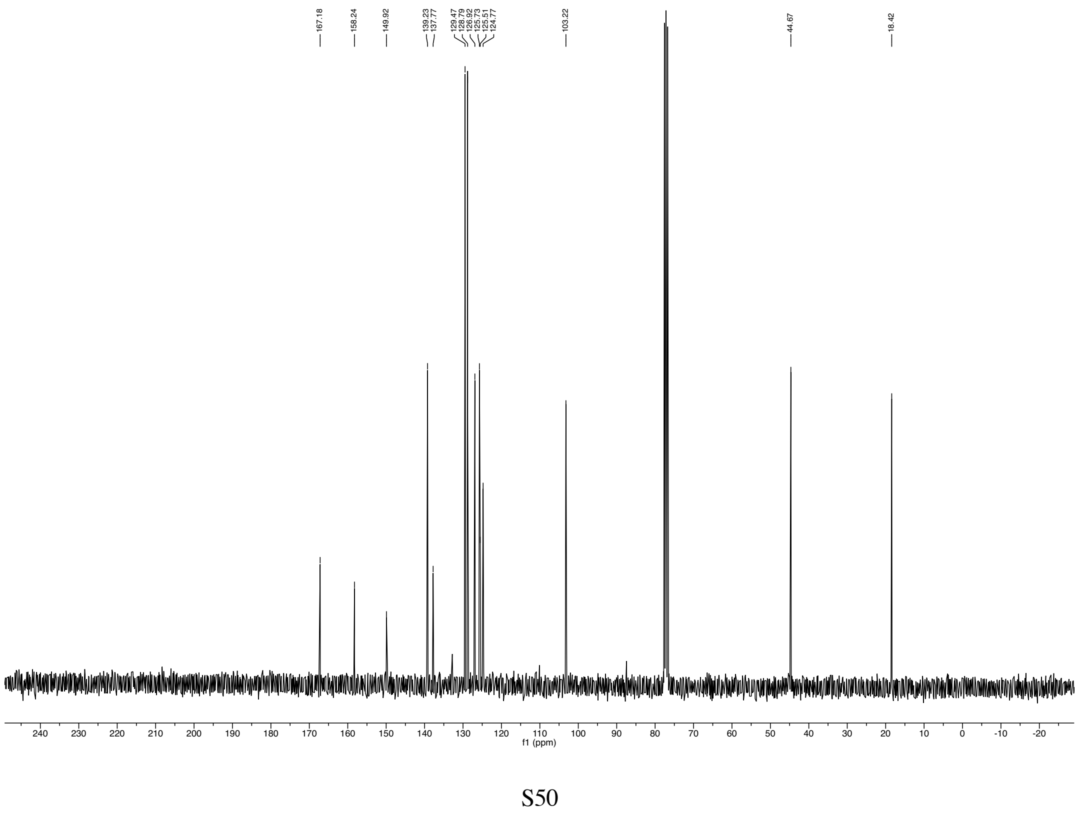

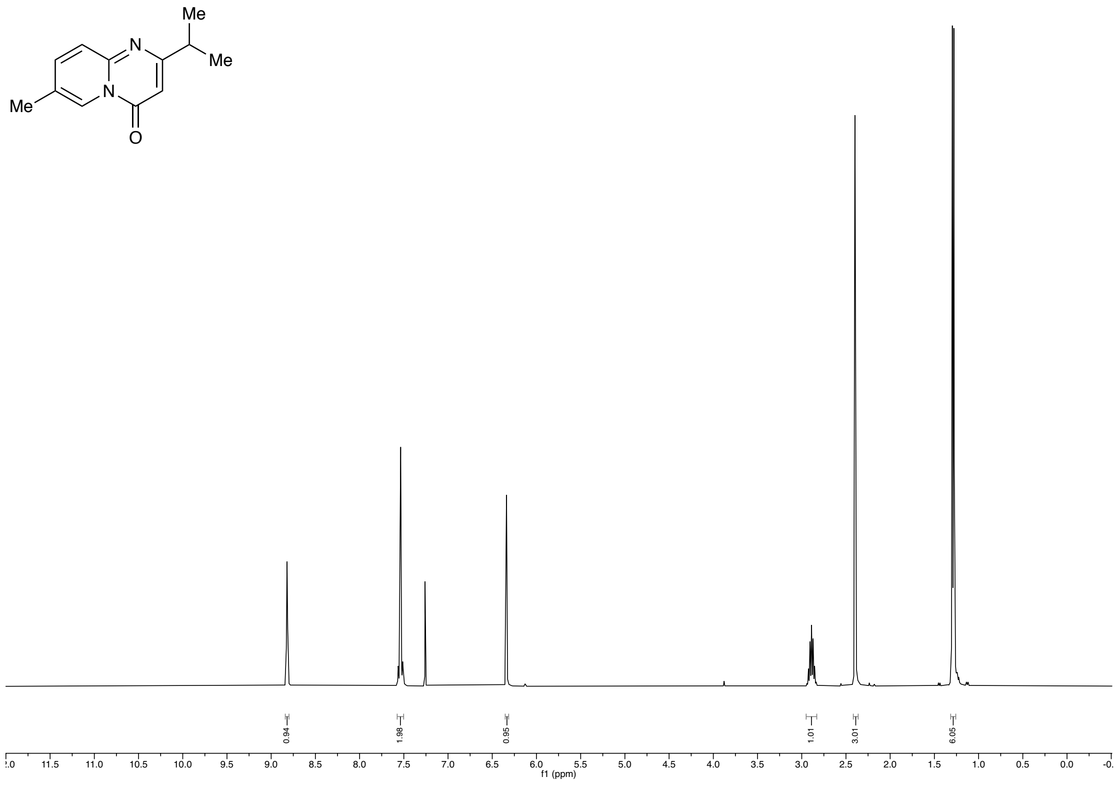

Me
N
Me
Me
N
O
0.94
1.98
0.95
1.01
3.01
6.05
f1 (ppm)

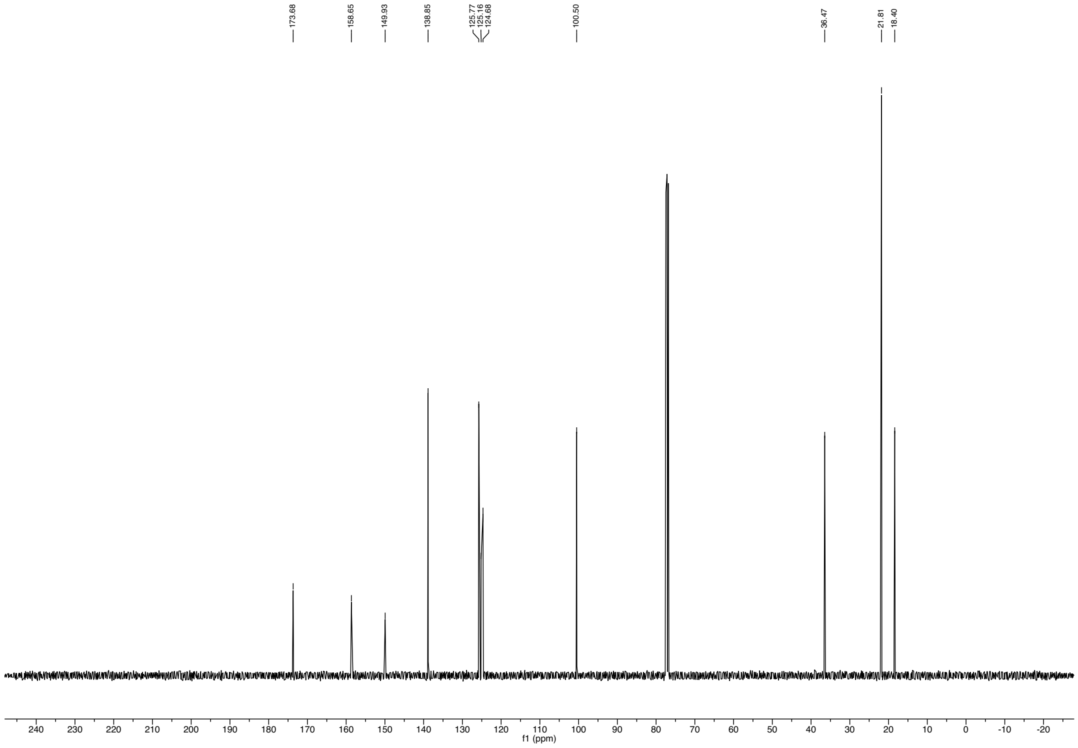

173.68
158.65
149.93
138.85
125.77
125.16
124.68
100.50
36.47
21.81
18.40
f1 (ppm)

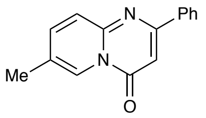

N
Ph
Me
N
O

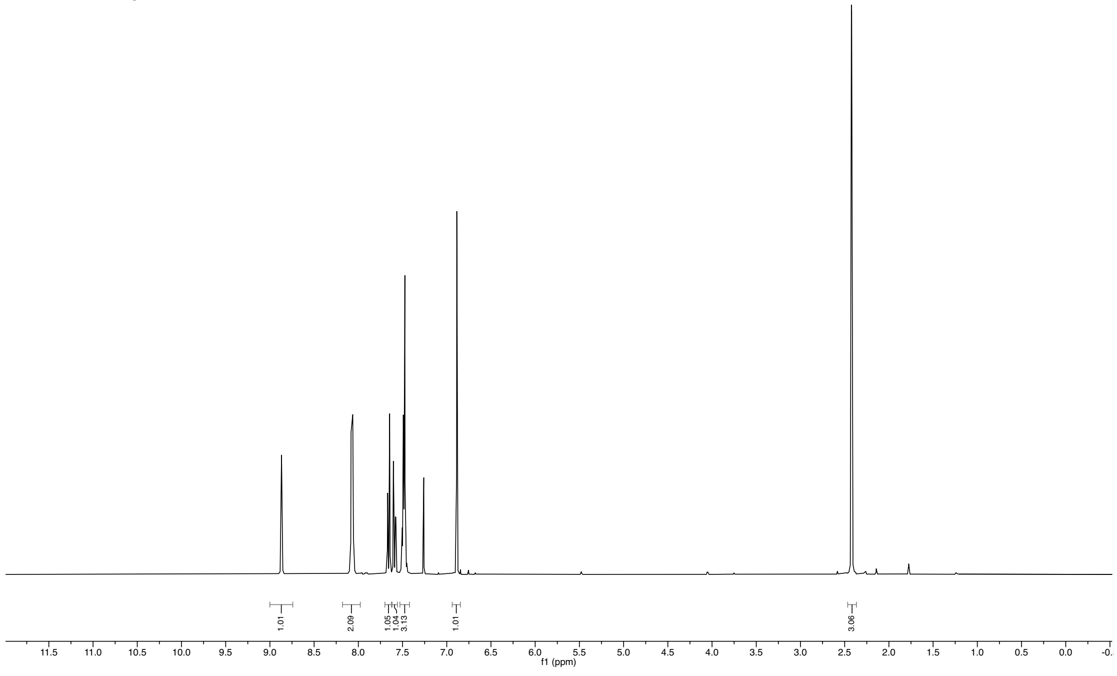

1.01
2.09
1.05
1.04
3.13
1.01
3.06
f1 (ppm)

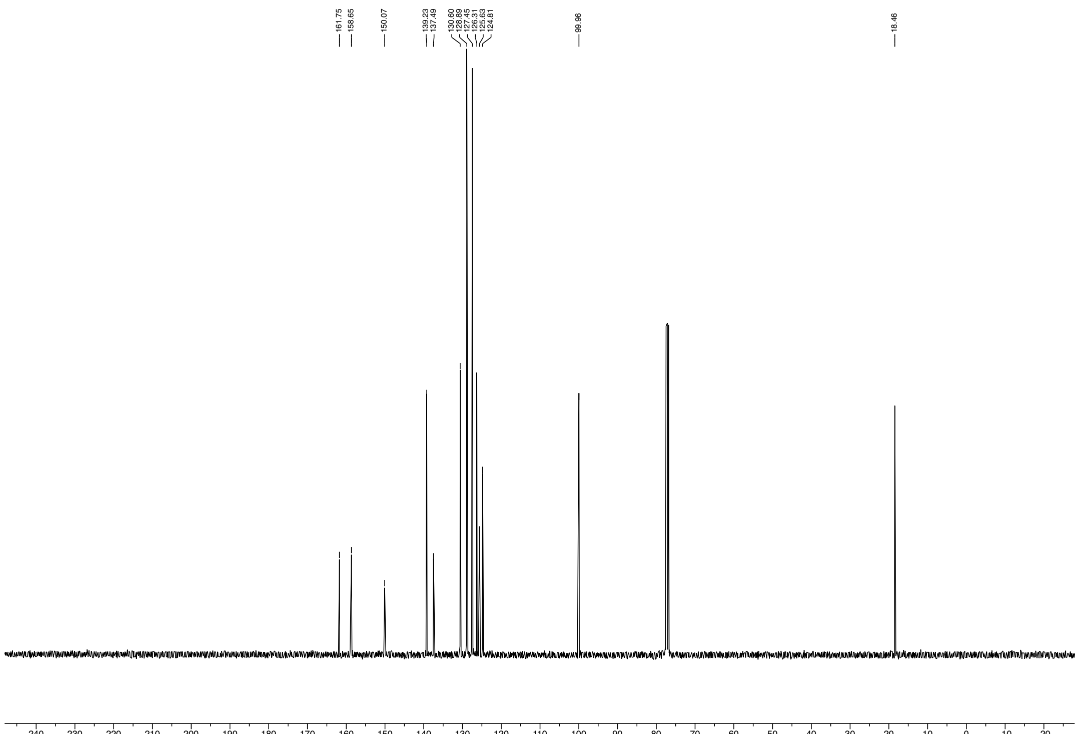

161.75
158.65
150.07
139.23
137.49
130.60
128.89
127.45
126.31
125.63
124.81
99.96
18.46
f1 (ppm)

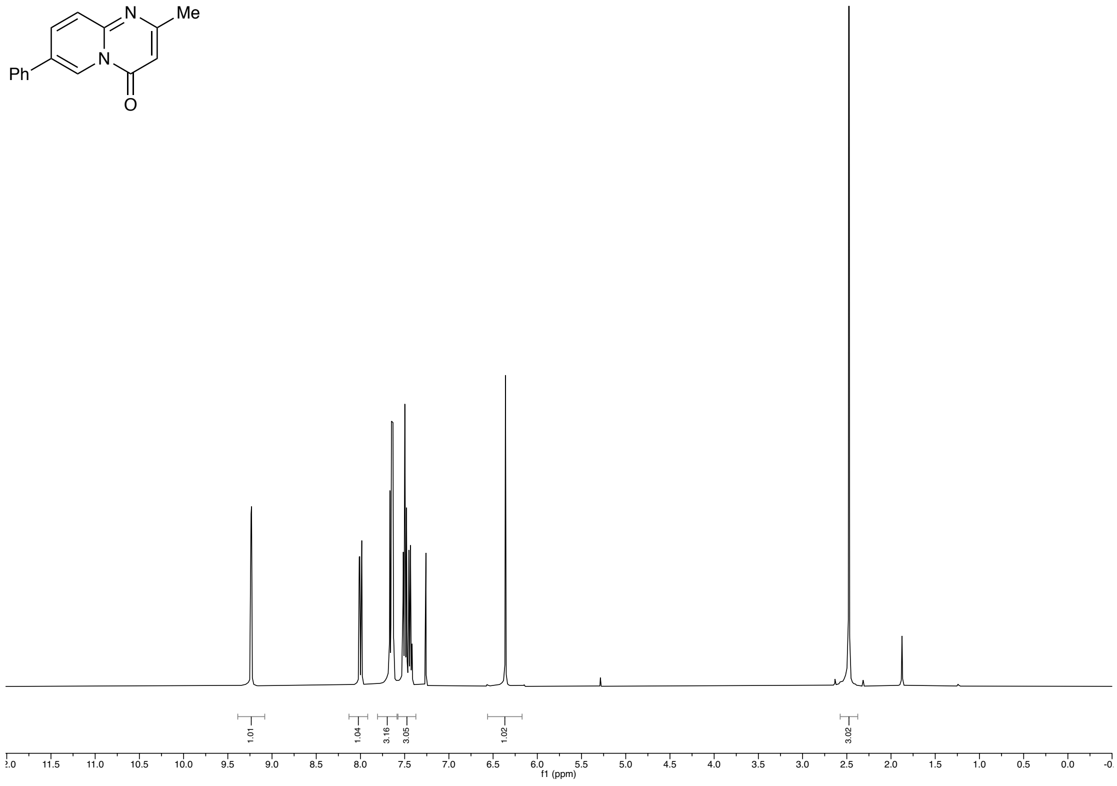
Me
N
N
Ph
O
1.01
1.04
3.16
3.05
1.02
3.02
f1 (ppm)

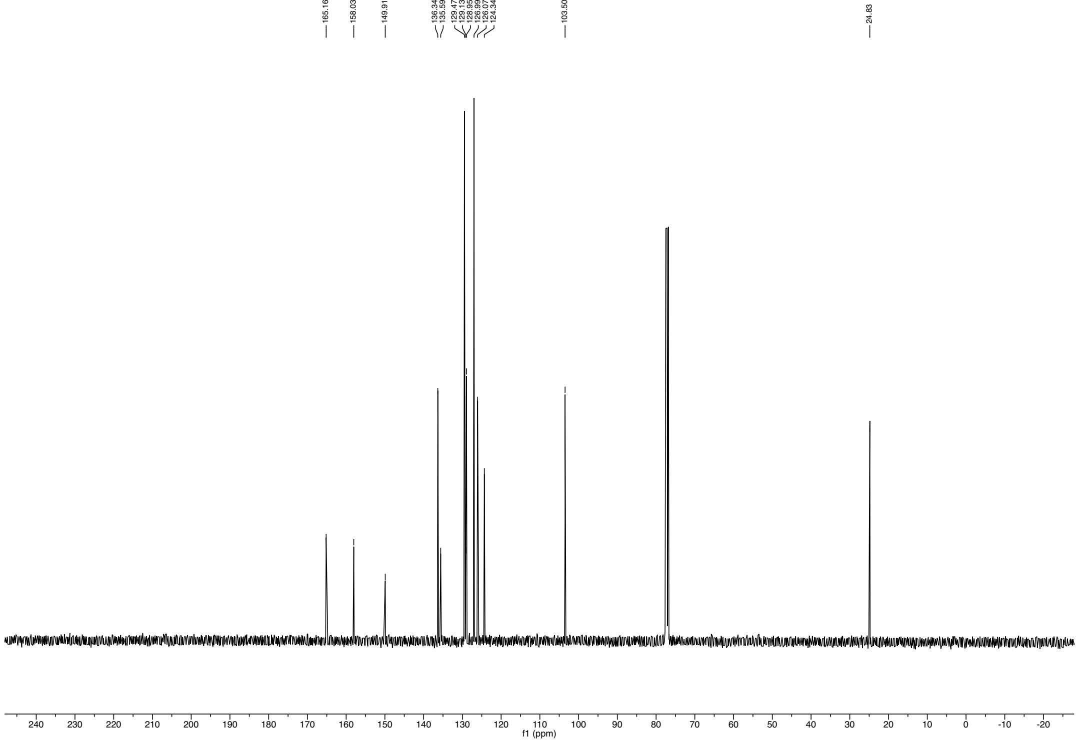
165.16
158.03
149.91
136.34
135.59
129.47
129.13
128.95
126.99
126.07
124.34
103.50
24.83
f1 (ppm)

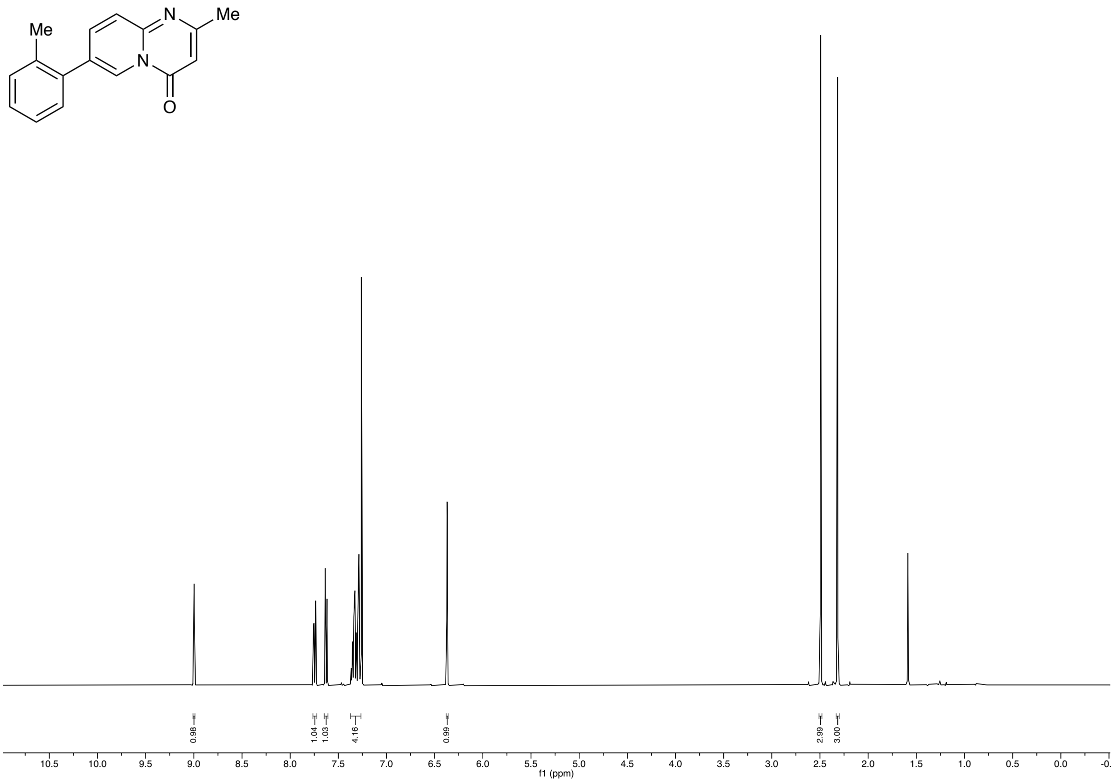
Me
N
Me
N
O
0.98
1.04
1.03
4.16
0.99
2.99
3.00
f1 (ppm)

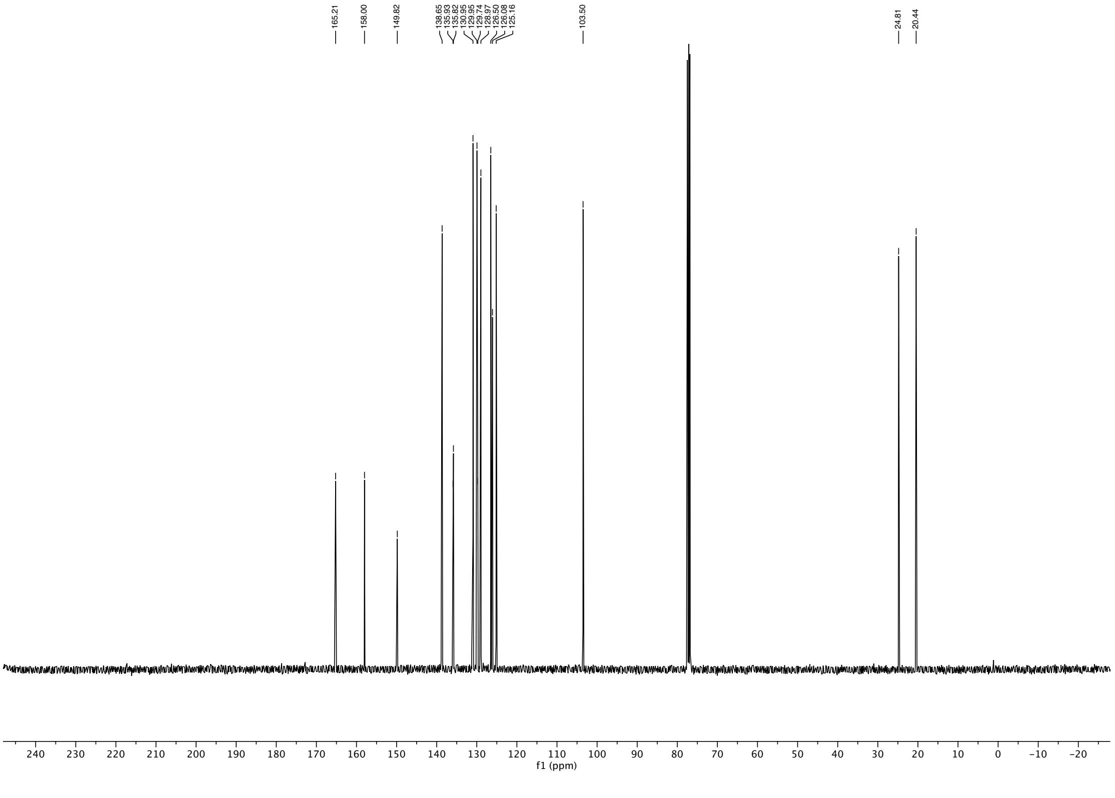
165.21
158.00
149.82
138.65
135.93
135.82
130.95
129.95
129.74
128.97
126.50
126.08
125.16
103.50
24.81
20.44
f1 (ppm)

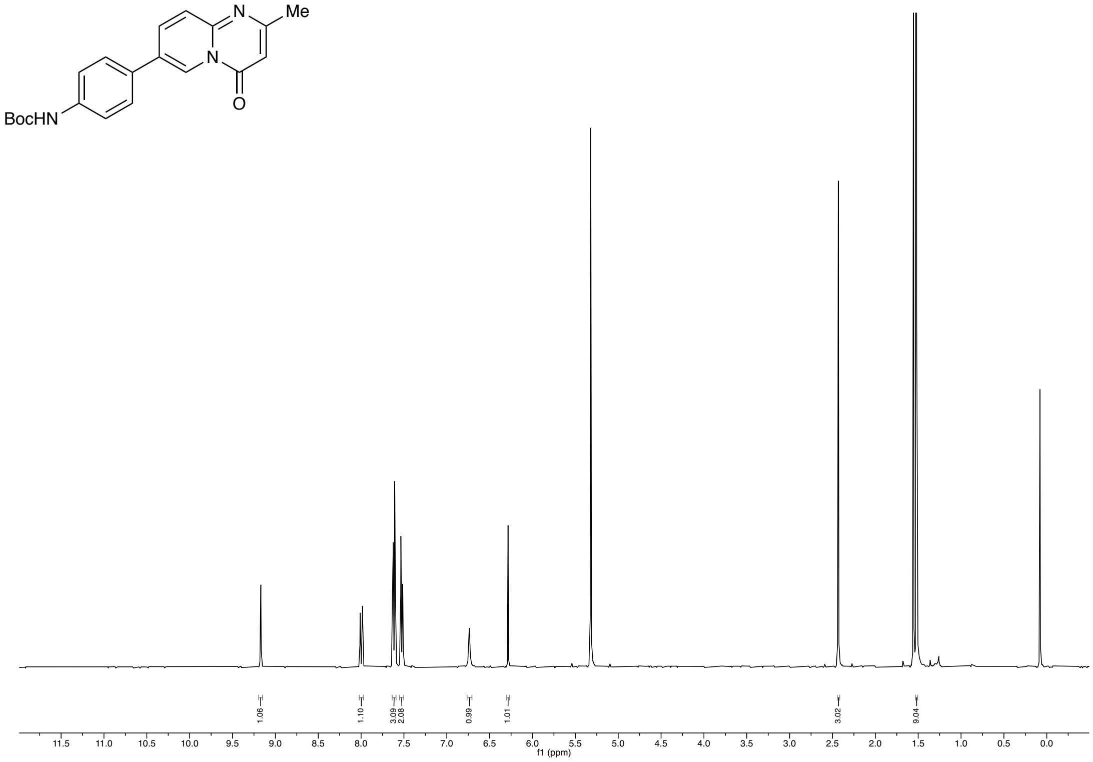

BocHN
N
N
Me
O
1.06
1.10
3.09
2.08
0.99
1.01
3.02
9.04
f1 (ppm)

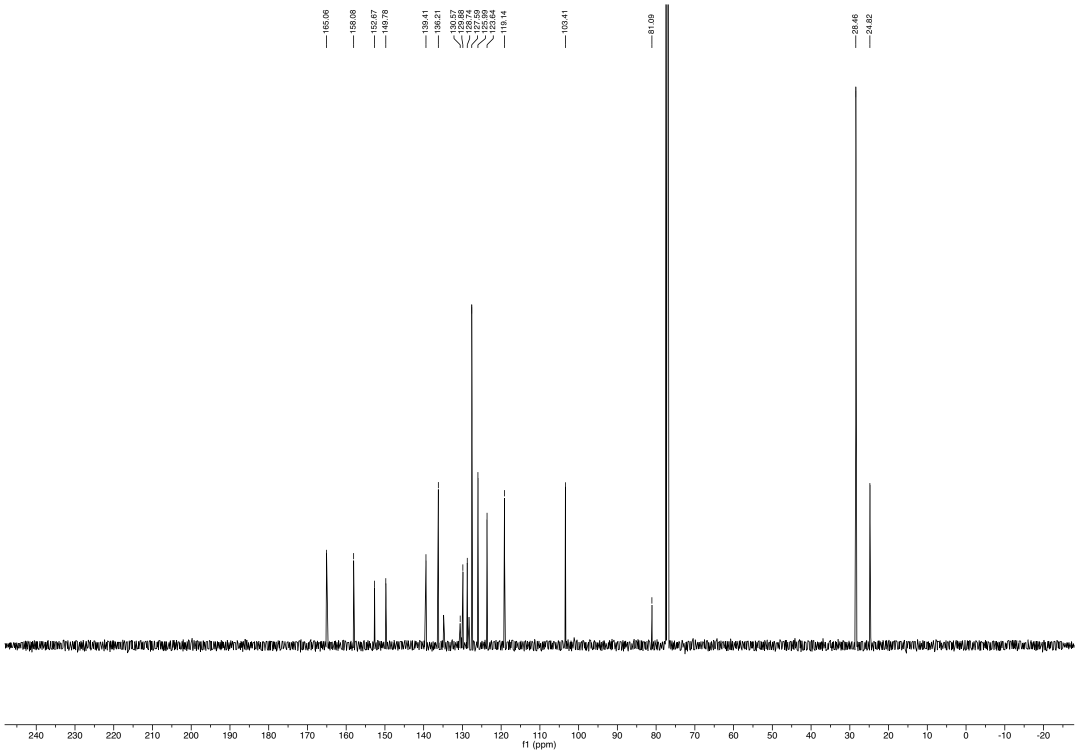

165.06
158.08
152.67
149.78
139.41
136.21
130.57
129.88
128.74
127.59
125.99
123.64
119.14
103.41
81.09
28.46
24.82
f1 (ppm)

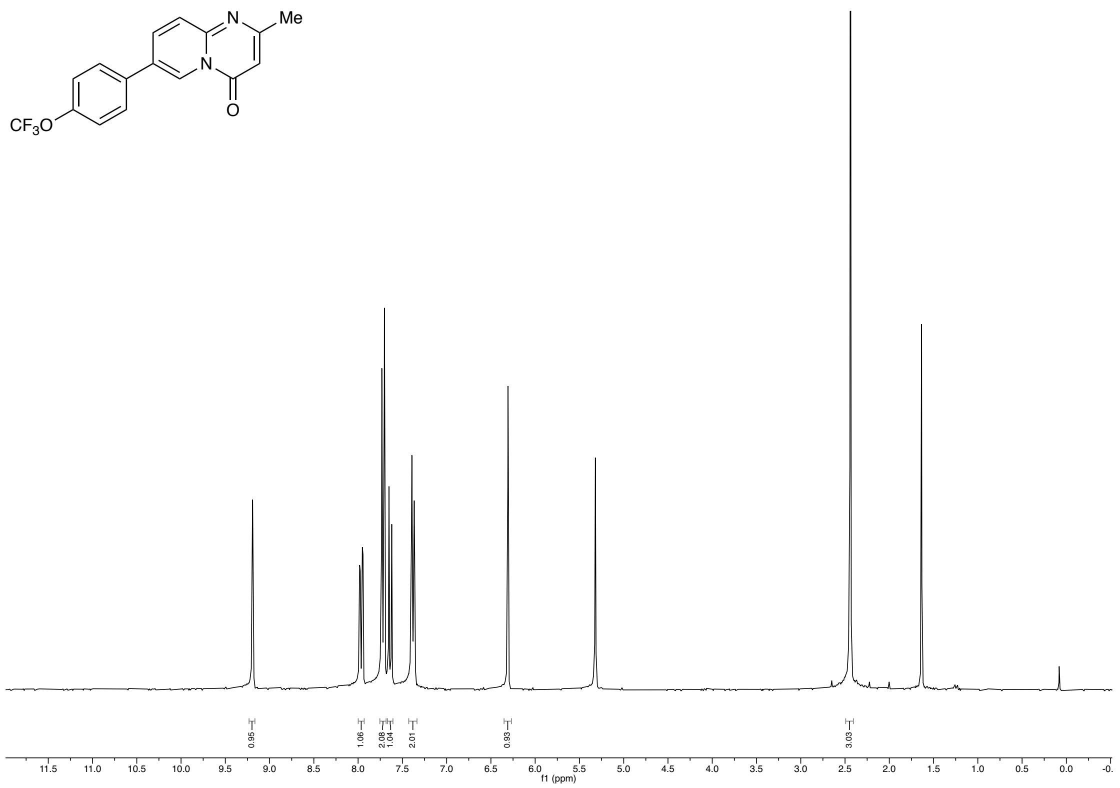

N
Me
N
O
CF3O
0.95
1.06
2.08
1.04
2.01
0.93
3.03
11.5 11.0 10.5 10.0 9.5 9.0 8.5 8.0 7.5 7.0 6.5 6.0 5.5 5.0 4.5 4.0 3.5 3.0 2.5 2.0 1.5 1.0 0.5 0.0
f1 (ppm)

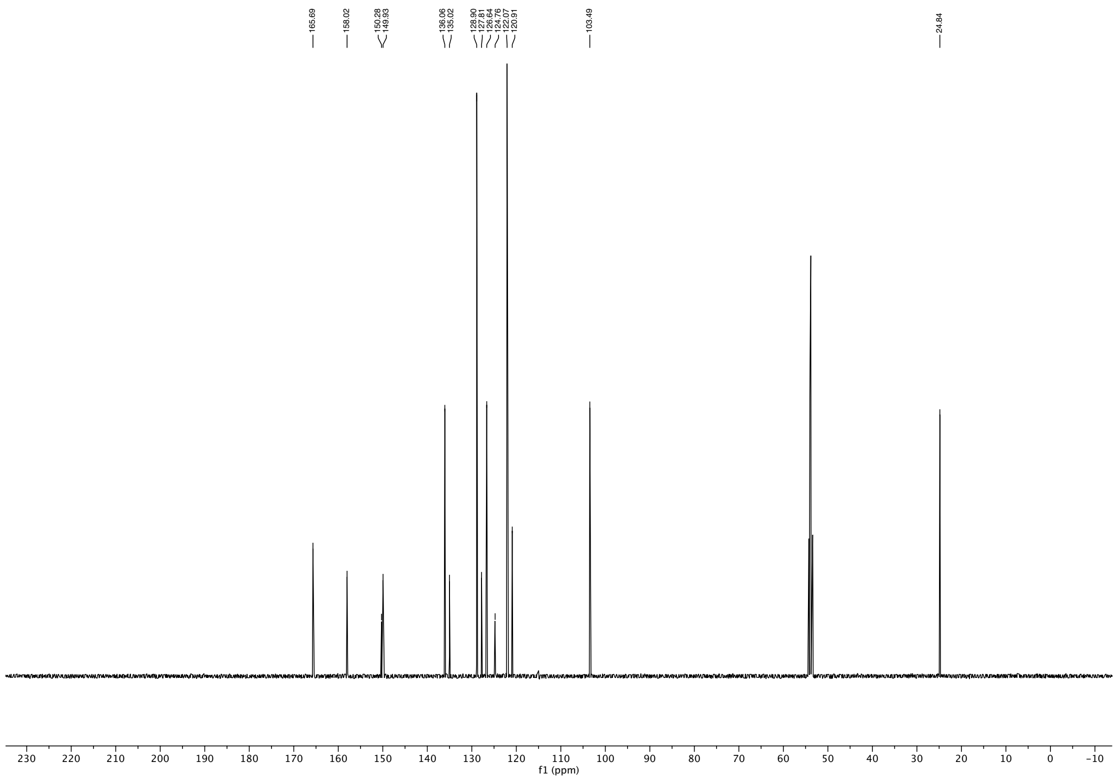

165.69
158.02
150.28
149.93
136.06
135.02
128.90
127.81
126.64
124.76
122.07
120.91
103.49
24.84
230 220 210 200 190 180 170 160 150 140 130 120 110 100 90 80 70 60 50 40 30 20 10 0 −10
f1 (ppm)

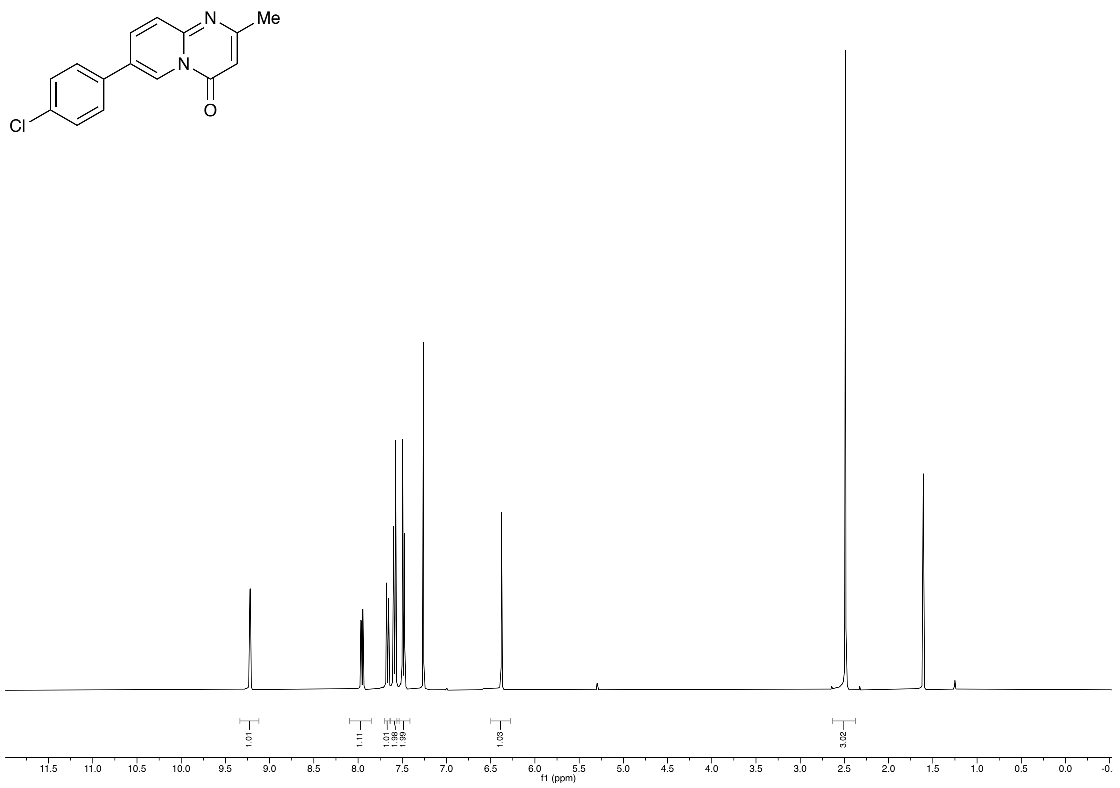

Me
N
N
O
Cl
1.01
1.11
1.01
1.98
1.99
1.03
3.02
f1 (ppm)

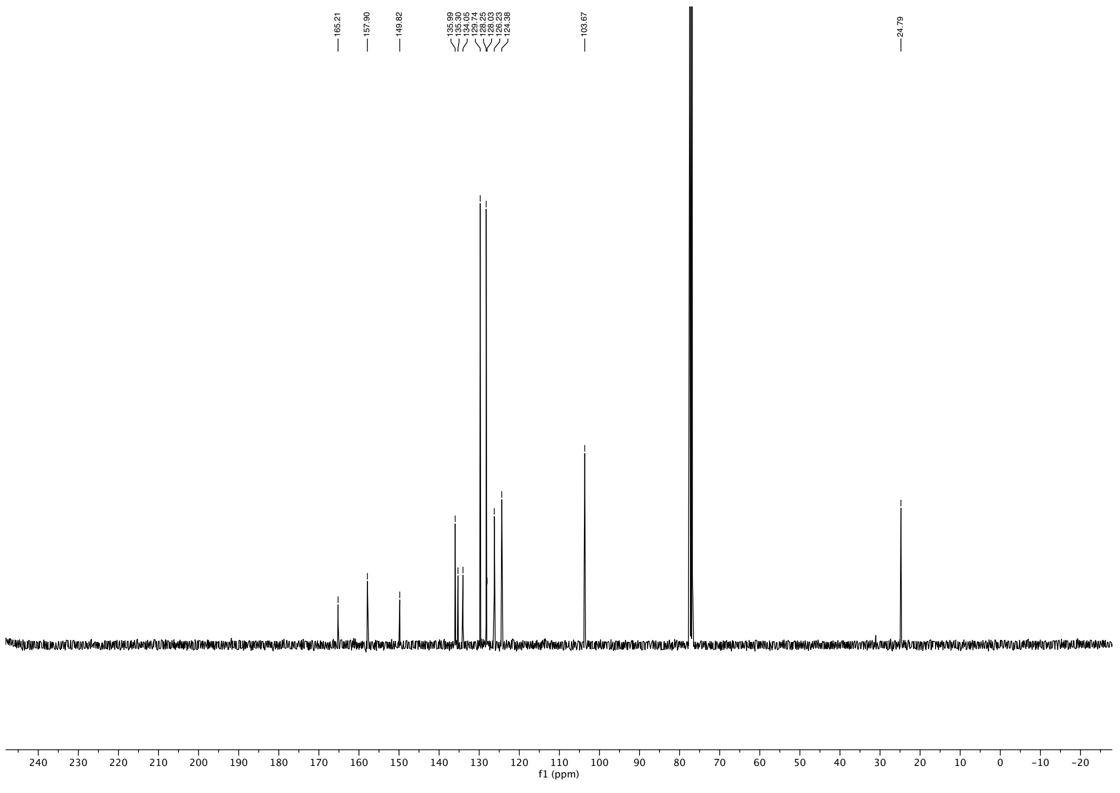

165.21
157.90
149.82
135.99
135.30
134.05
129.74
128.25
128.03
126.23
124.38
103.67
24.79
f1 (ppm)

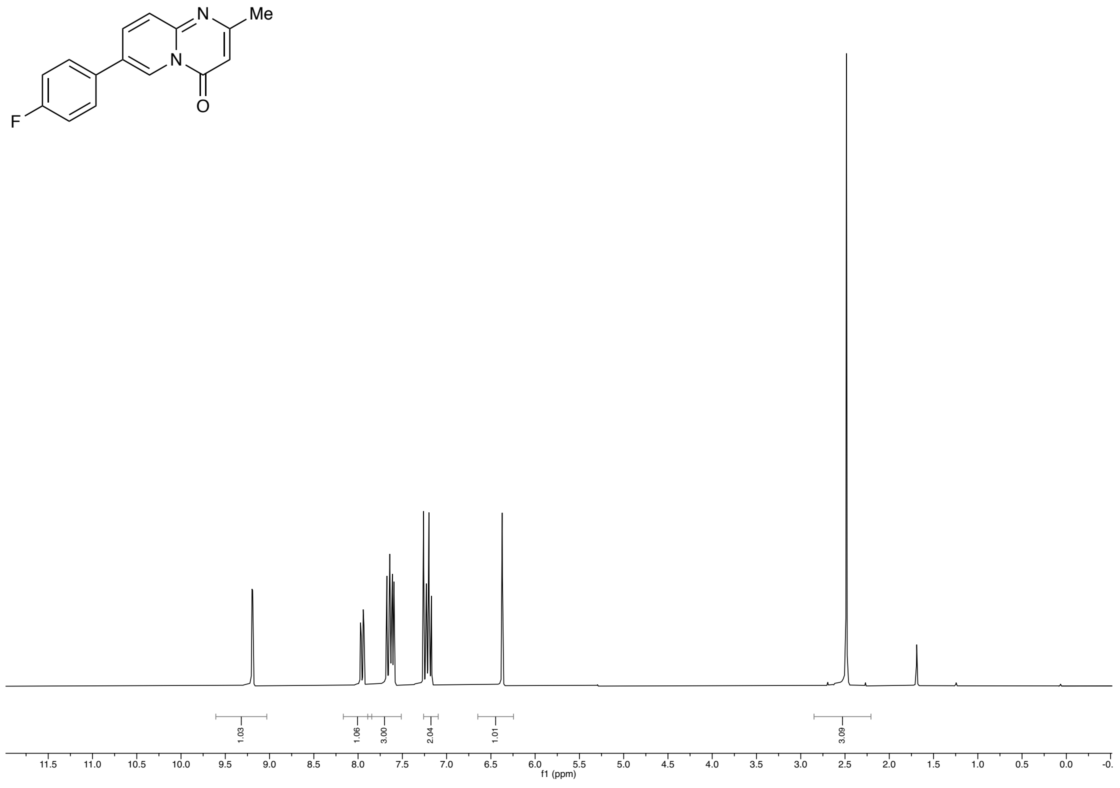
N
Me
N
O
F
1.03
1.06
3.00
2.04
1.01
3.09
f1 (ppm)

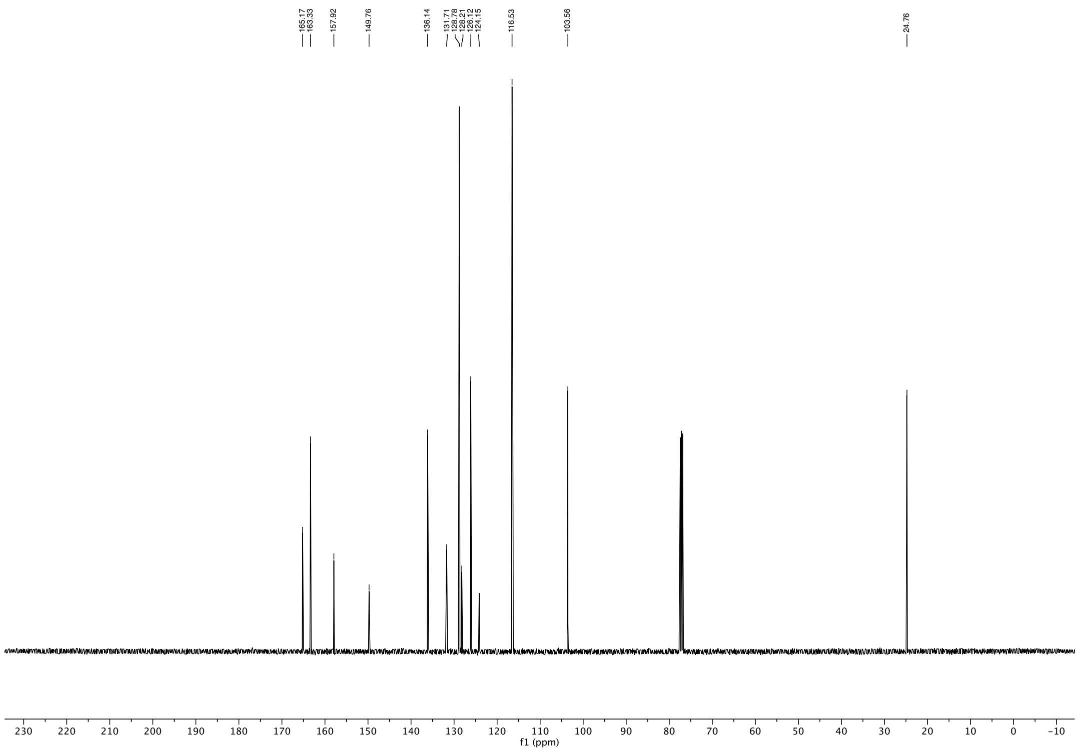
165.17
163.33
157.92
149.76
136.14
131.71
128.78
128.21
126.12
124.15
116.53
103.56
24.76
f1 (ppm)

(J) NMR spectra of products

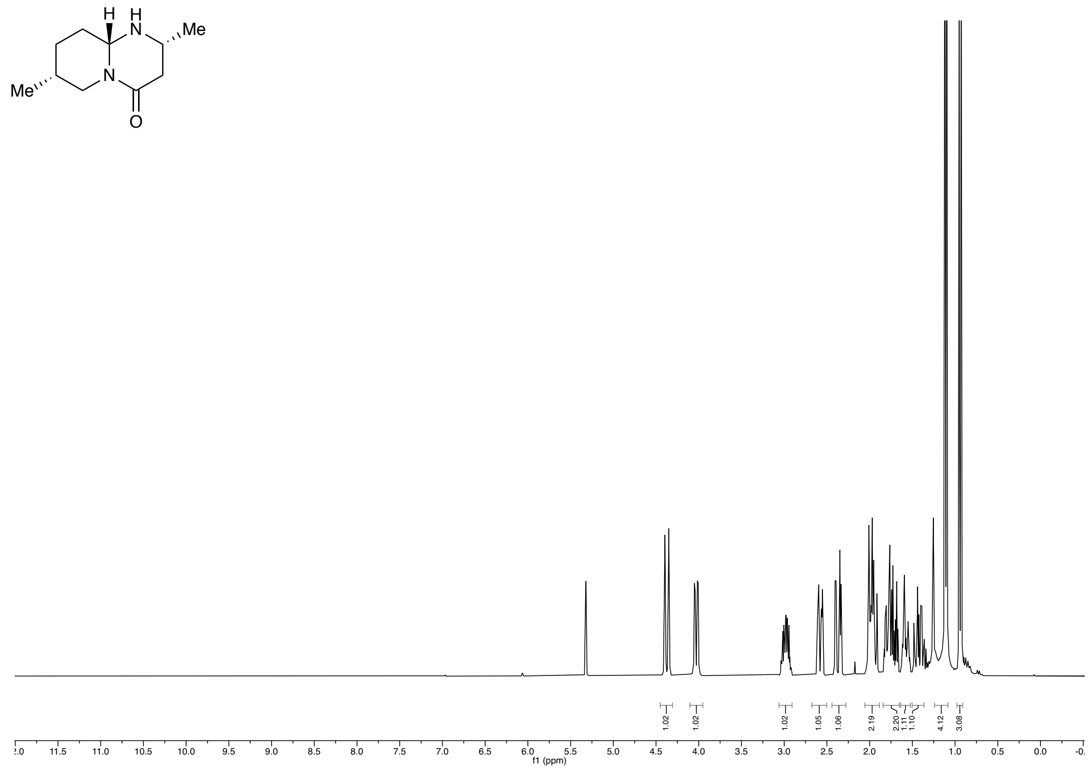

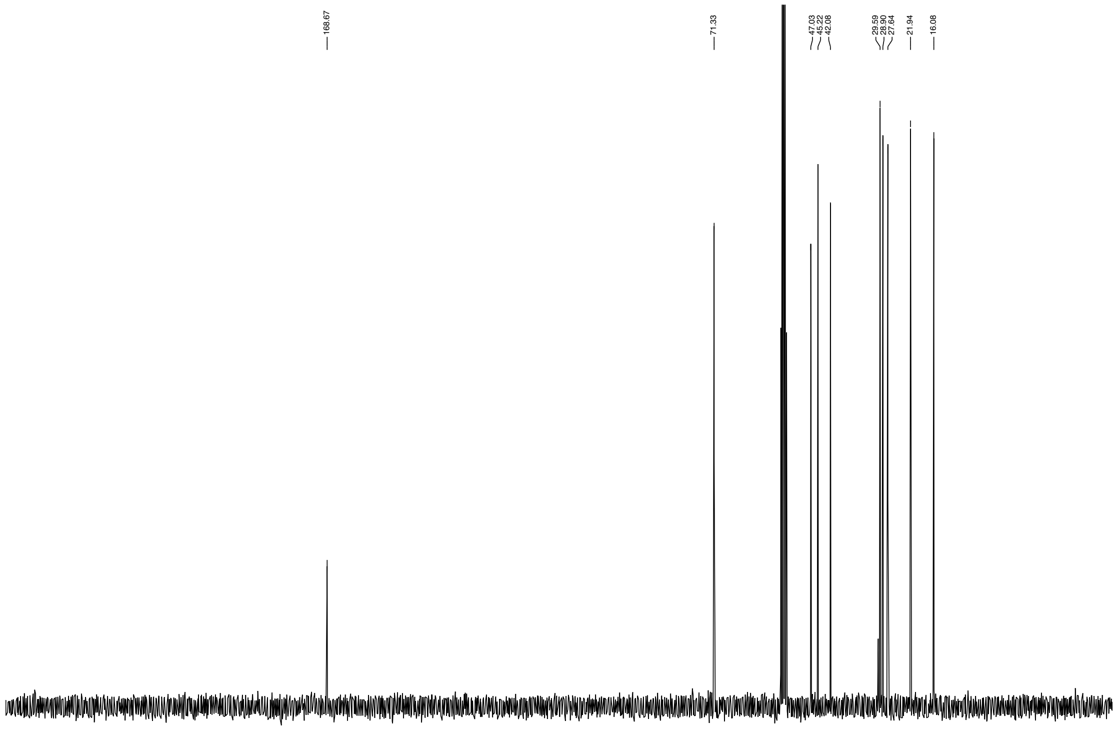

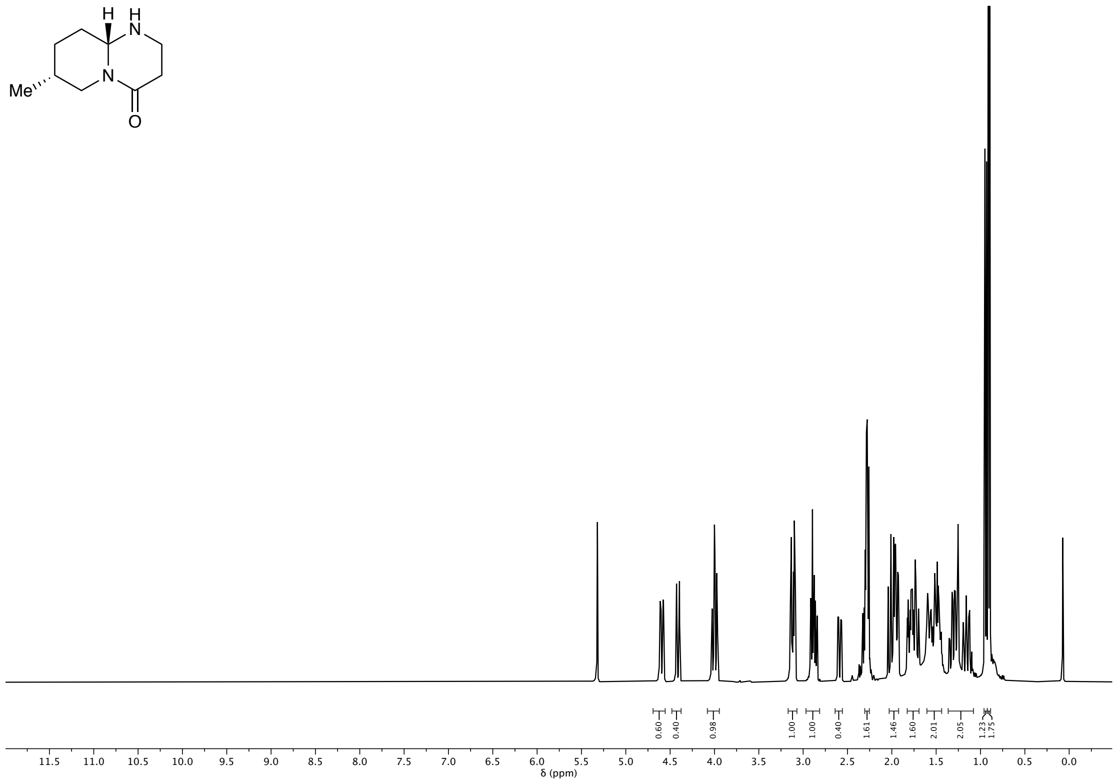
Me
H
N
H
N
O
0.60
0.40
0.98
1.00
1.00
0.40
1.61
1.46
1.60
2.01
2.05
1.23
1.75
11.5 11.0 10.5 10.0 9.5 9.0 8.5 8.0 7.5 7.0 6.5 6.0 5.5 5.0 4.5 4.0 3.5 3.0 2.5 2.0 1.5 1.0 0.5 0.0
δ (ppm)

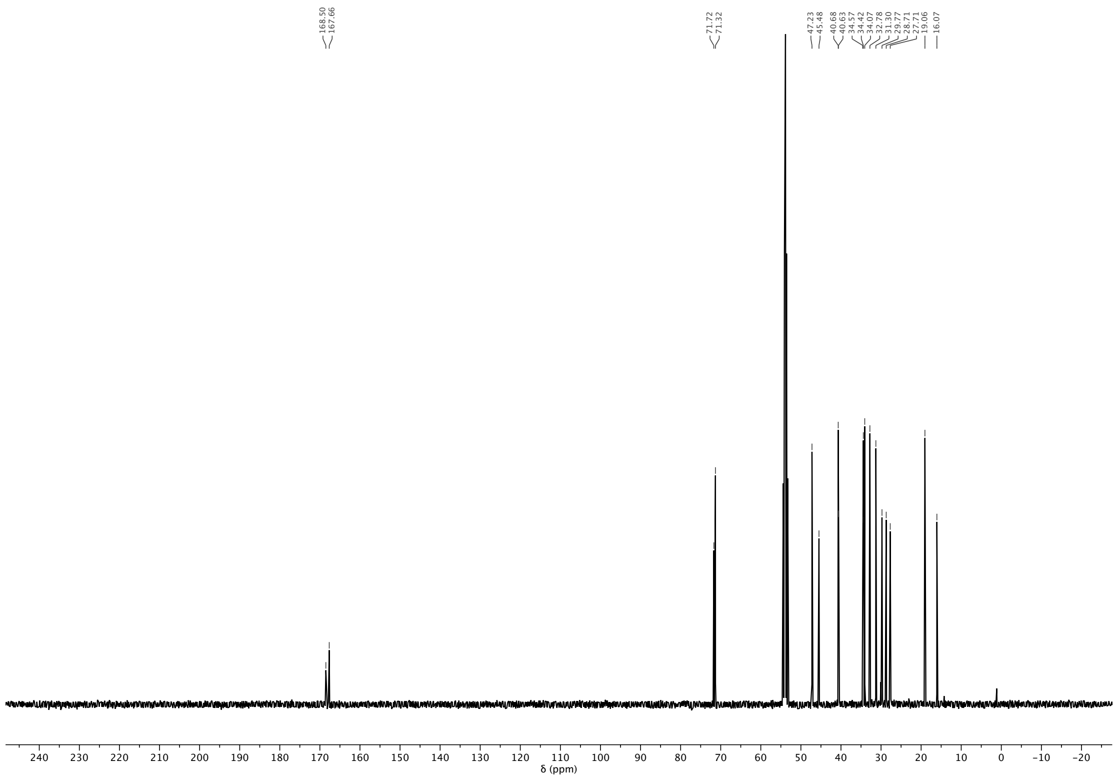
168.50
167.66
71.72
71.32
47.23
45.48
40.68
40.63
34.57
34.42
34.07
32.78
31.30
29.77
28.71
27.71
19.06
16.07
240 230 220 210 200 190 180 170 160 150 140 130 120 110 100 90 80 70 60 50 40 30 20 10 0 −10 −20
δ (ppm)

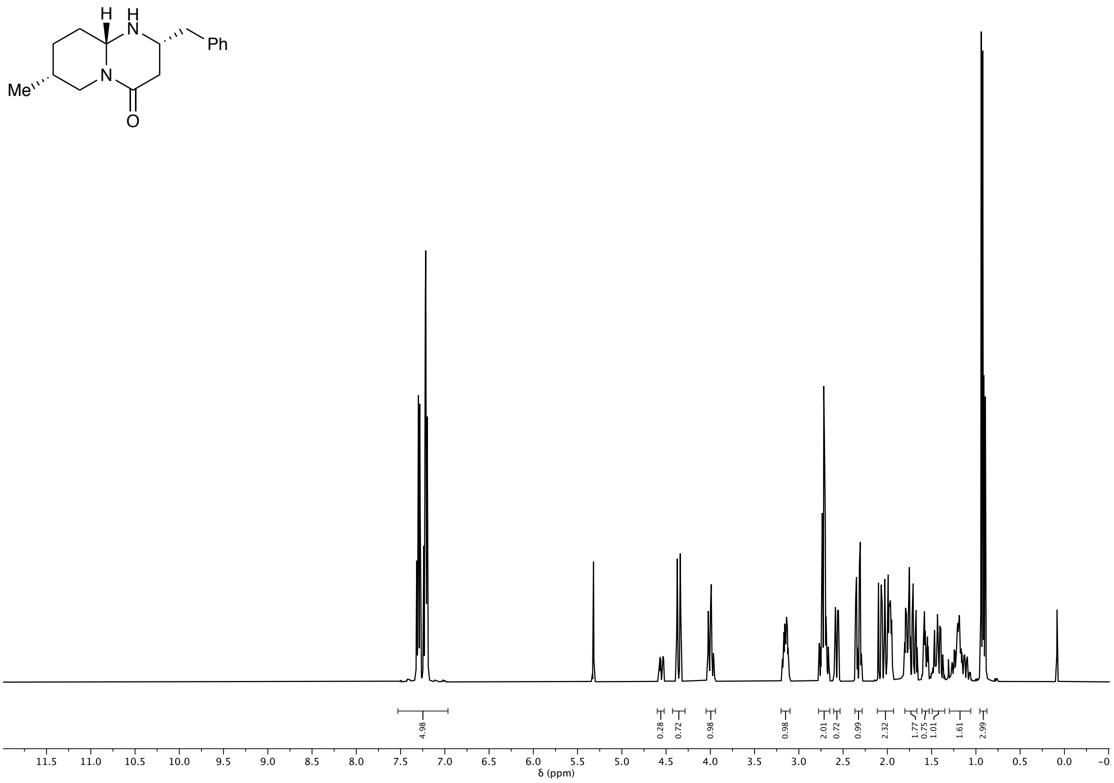
H
N
N
Ph
Me
O
4.98
0.28
0.72
0.98
0.98
2.01
0.72
0.99
2.32
1.77
0.75
1.01
1.61
2.99
11.5 11.0 10.5 10.0 9.5 9.0 8.5 8.0 7.5 7.0 6.5 6.0 5.5 5.0 4.5 4.0 3.5 3.0 2.5 2.0 1.5 1.0 0.5 0.0 −0
δ (ppm)

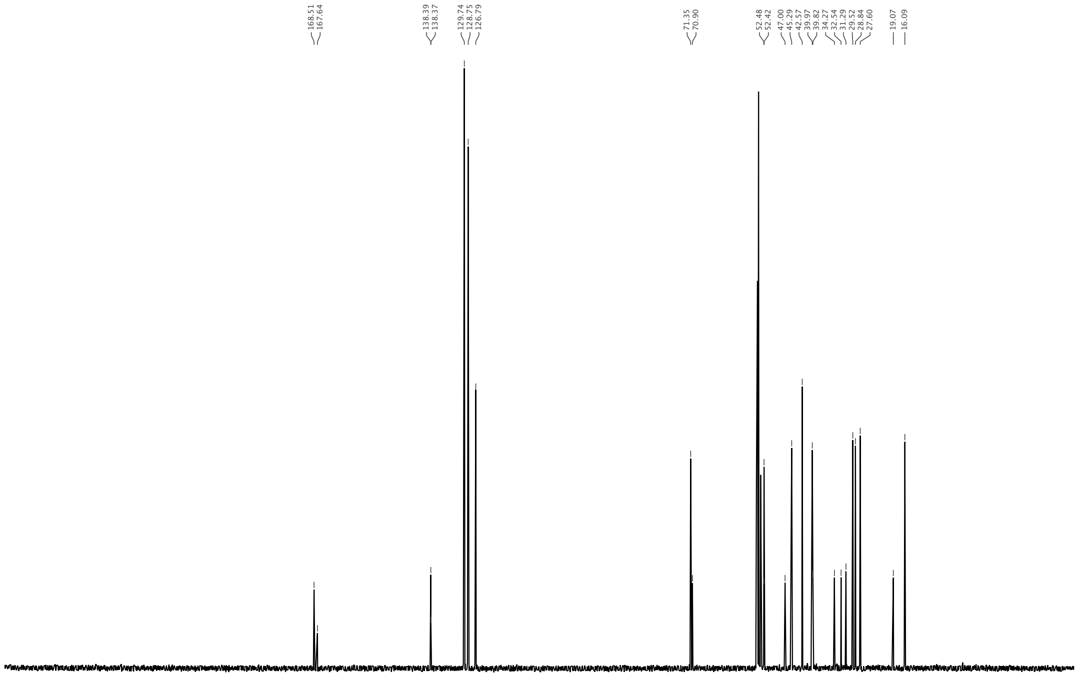
168.51
167.64
138.39
138.37
129.74
128.75
126.79
71.35
70.90
52.48
52.42
47.00
45.29
42.57
39.97
39.82
34.27
32.54
31.29
29.52
28.84
27.60
19.07
16.09
240 230 220 210 200 190 180 170 160 150 140 130 120 110 100 90 80 70 60 50 40 30 20 10 0 −10 −20
δ (ppm)

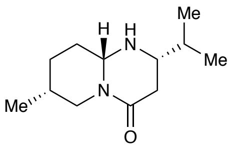
Me
H
N
H
Me
Me
N
O

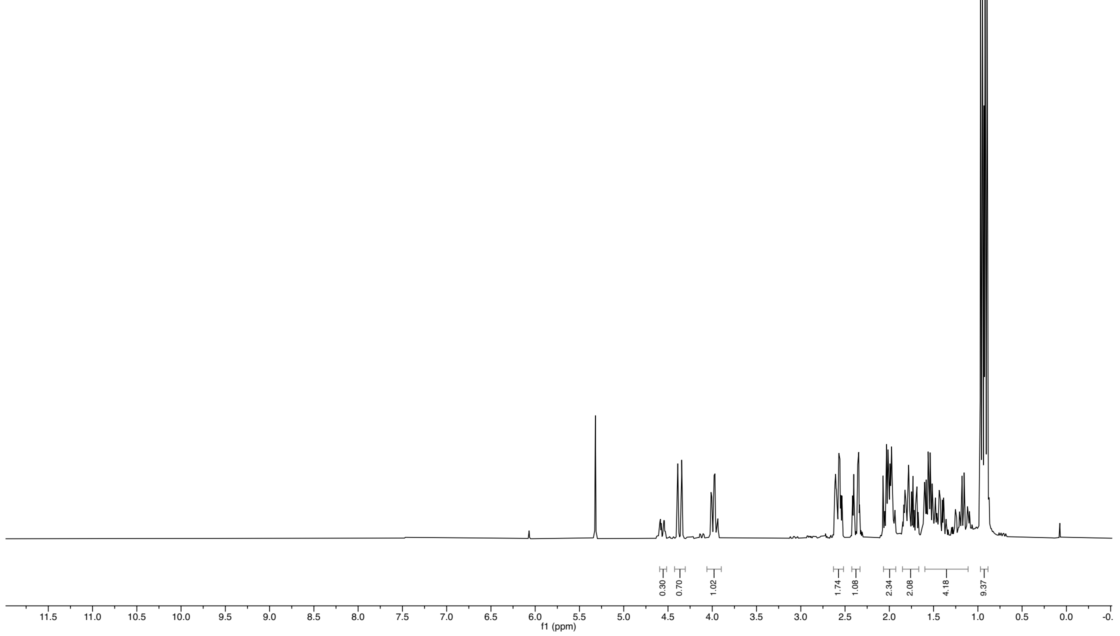
0.30
0.70
1.02
1.74
1.08
2.34
2.08
4.18
9.37
f1 (ppm)

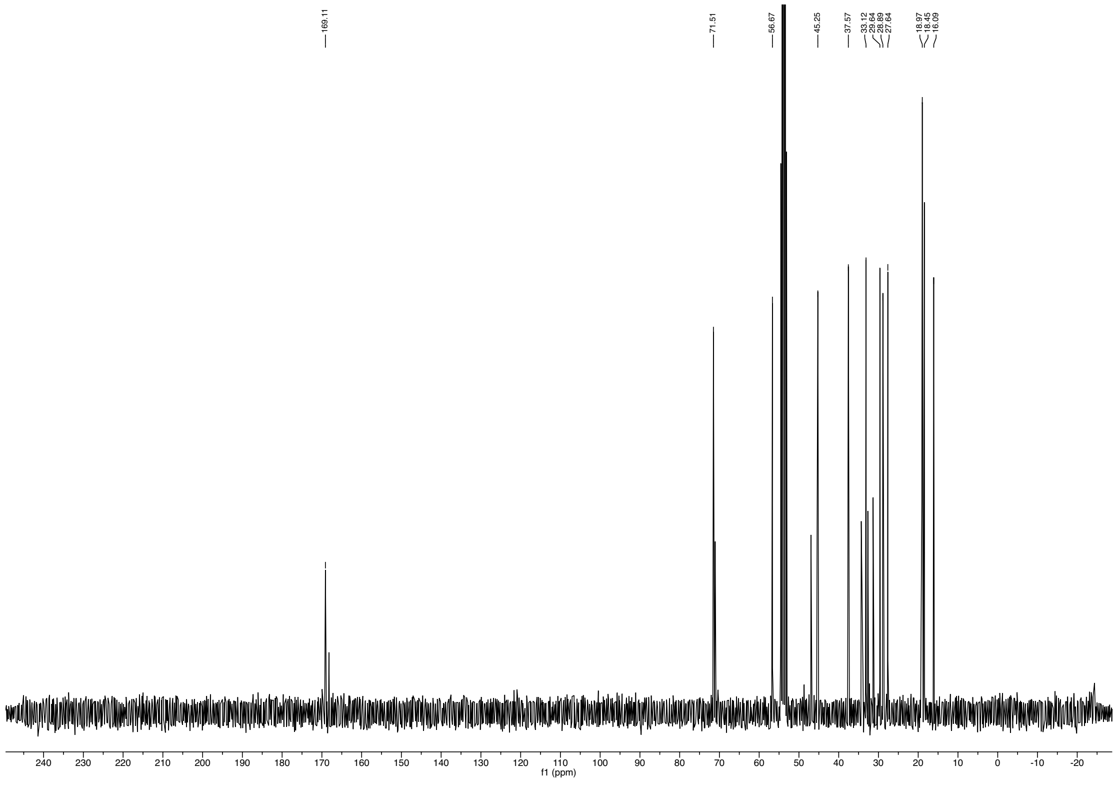
169.11
71.51
56.67
45.25
37.57
33.12
29.64
28.89
27.64
18.97
18.45
16.09
f1 (ppm)

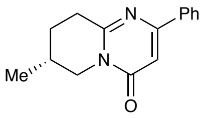
Ph
Me
O

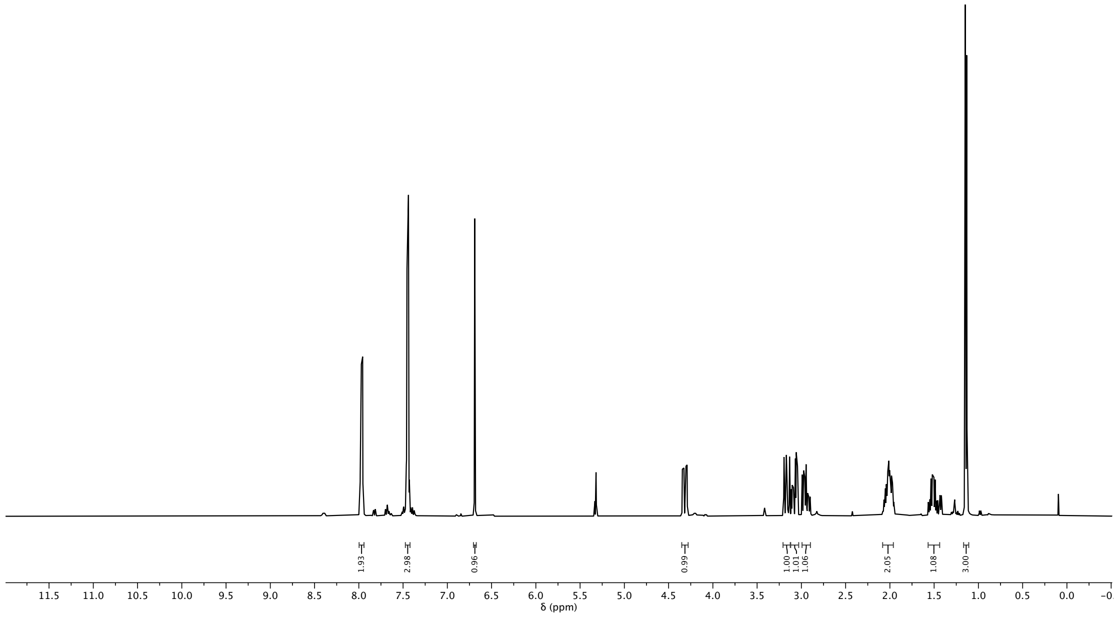
1.93
2.98
0.96
0.99
1.00
1.01
1.06
2.05
1.08
3.00
11.5 11.0 10.5 10.0 9.5 9.0 8.5 8.0 7.5 7.0 6.5 6.0 5.5 5.0 4.5 4.0 3.5 3.0 2.5 2.0 1.5 1.0 0.5 0.0
δ (ppm)

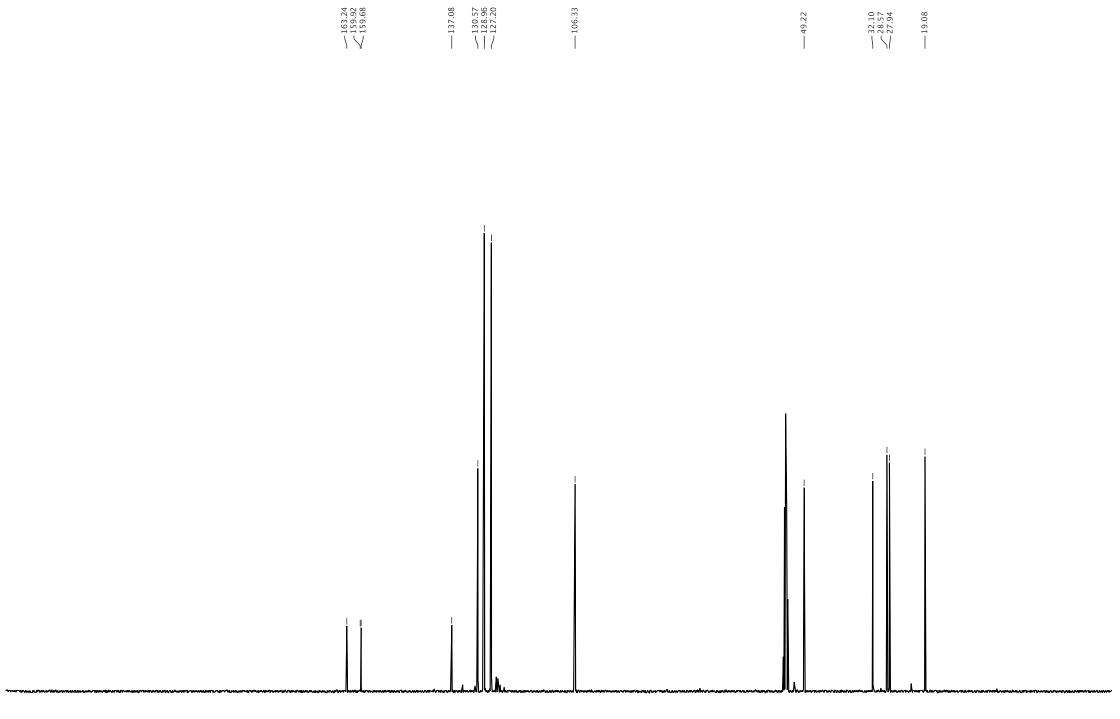
163.24
159.92
159.68
137.08
130.57
128.96
127.20
106.33
49.22
32.10
28.57
27.94
19.08
240 230 220 210 200 190 180 170 160 150 140 130 120 110 100 90 80 70 60 50 40 30 20 10 0 −10 −20
δ (ppm)

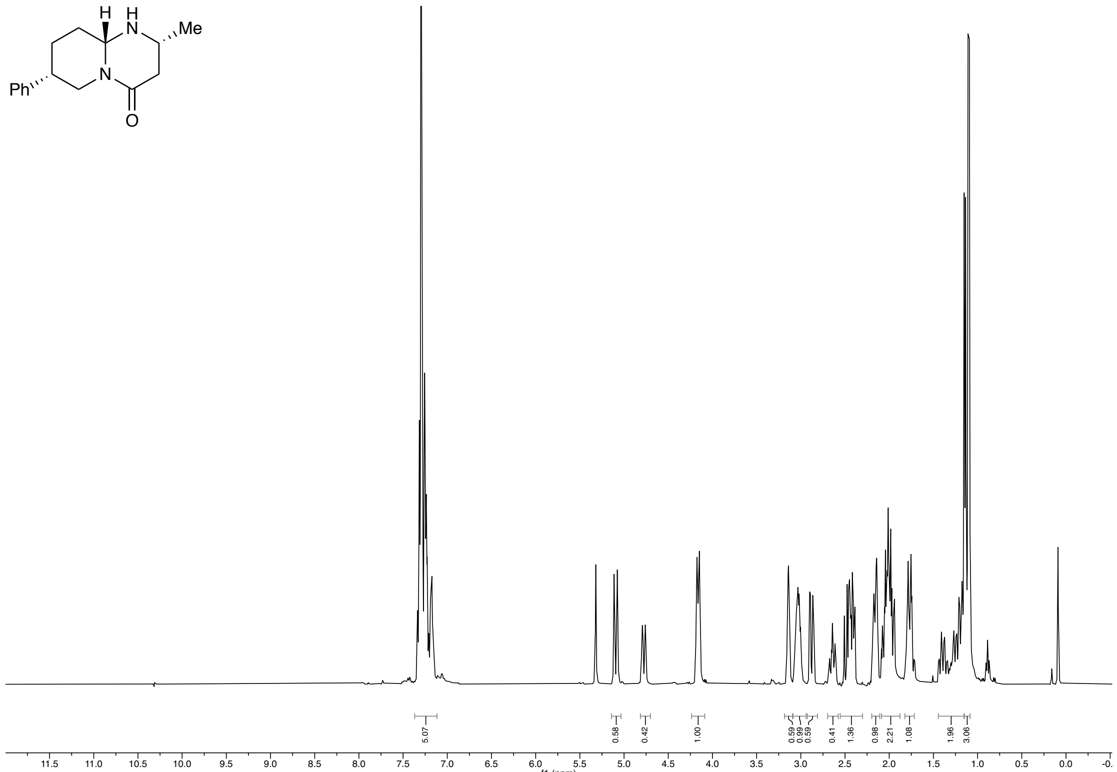
H
N
H
Me
Ph
N
O
5.07
0.58
0.42
1.00
0.59
0.99
0.59
0.41
1.36
0.98
2.21
1.08
1.96
3.06
f1 (ppm)

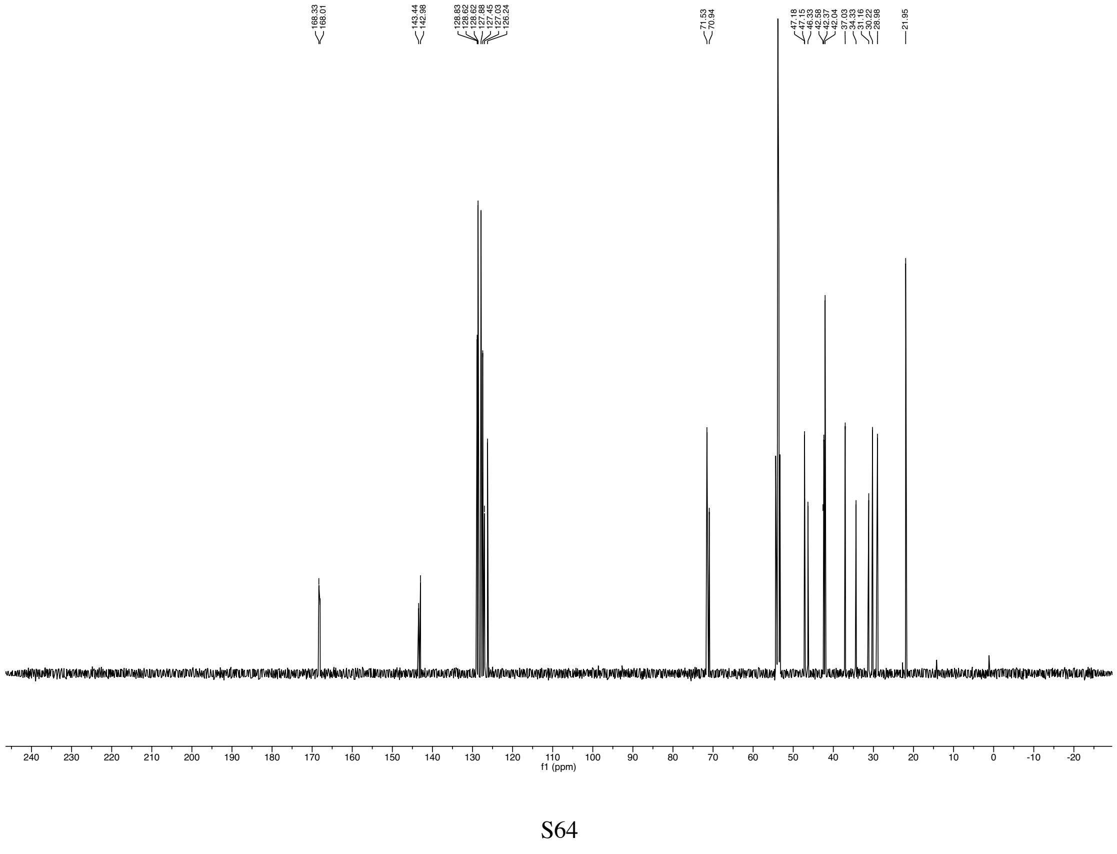
168.33
168.01
143.44
142.98
128.83
128.62
128.62
127.88
127.45
127.03
126.24
71.53
70.94
47.18
47.15
46.33
42.58
42.37
42.04
37.03
34.33
31.16
30.22
28.98
21.95
f1 (ppm)

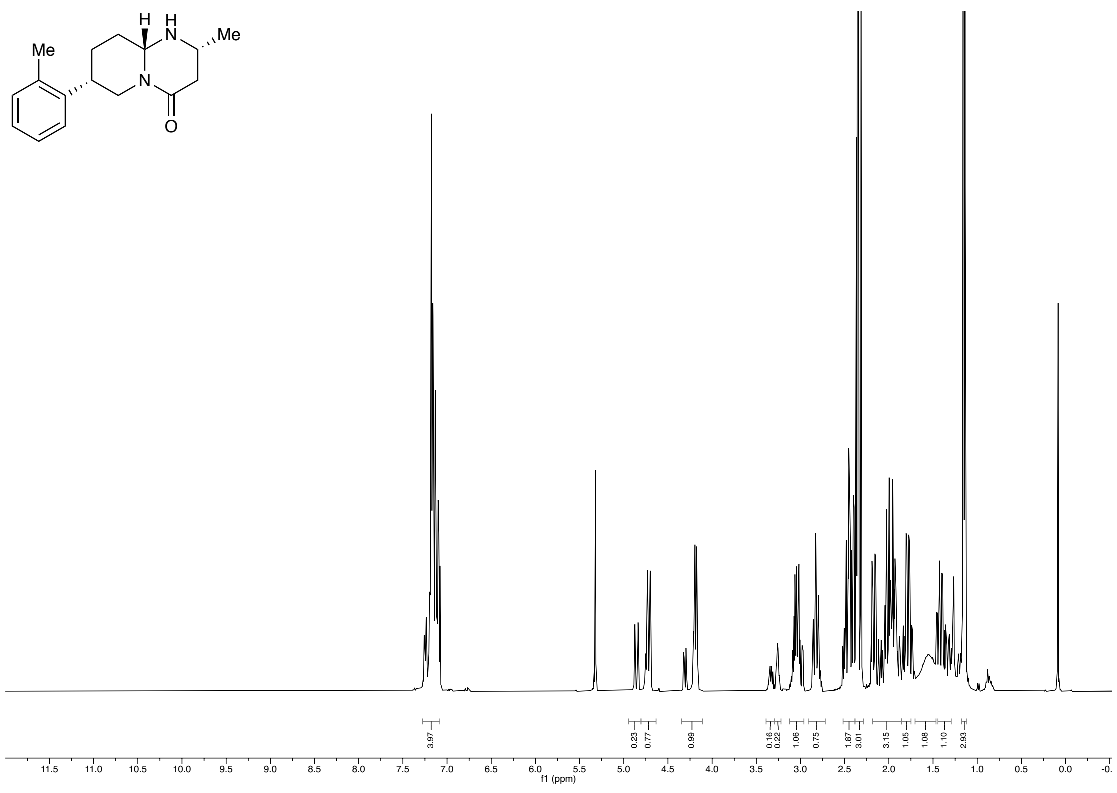
Me
H
N
Me
N
O
f1 (ppm)

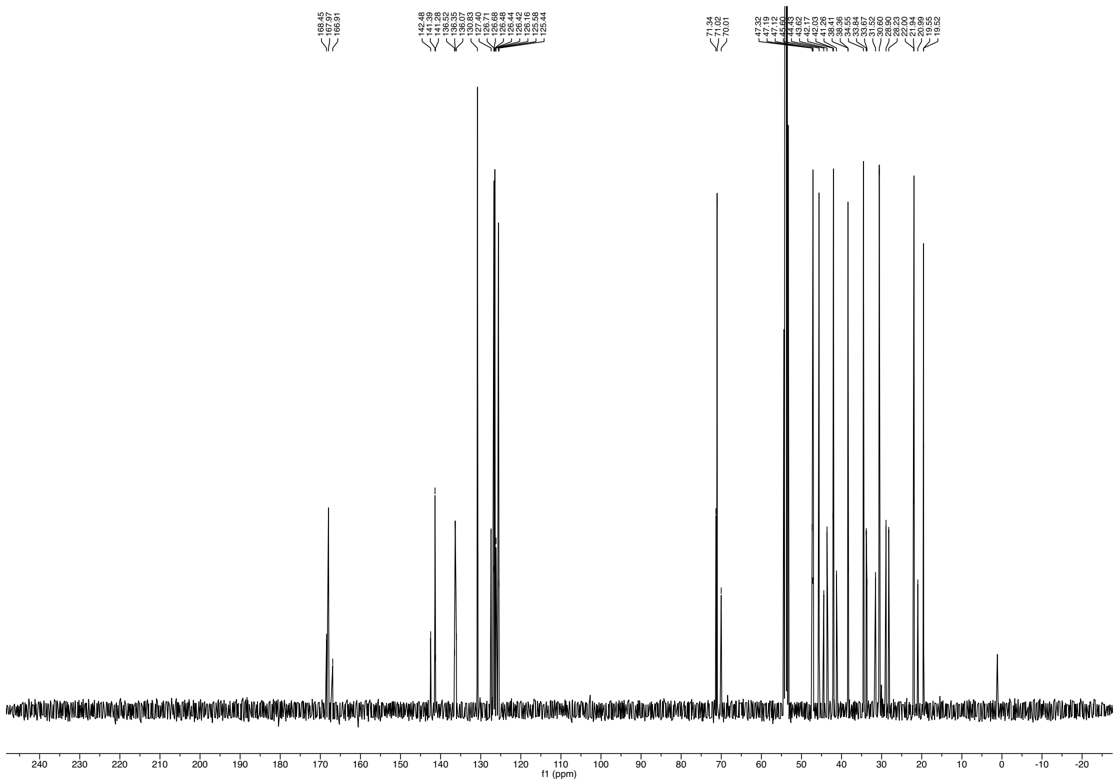
f1 (ppm)

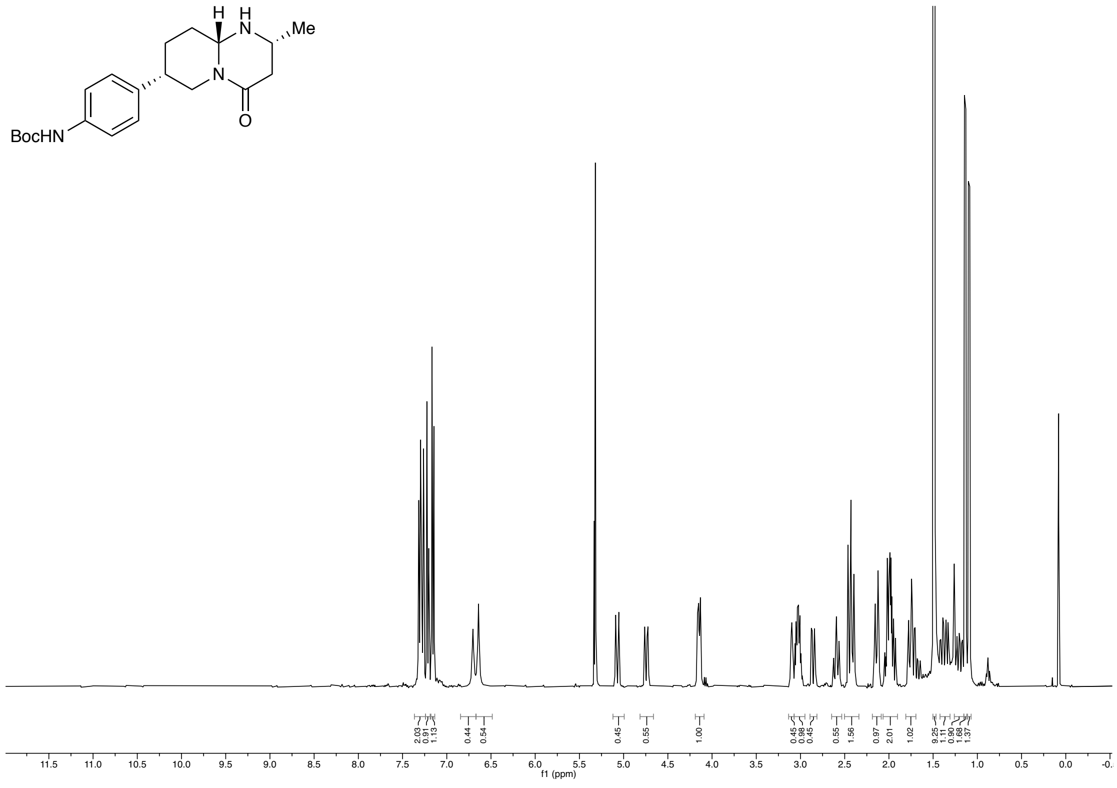
BocHN
N
H
N
Me
O
f1 (ppm)

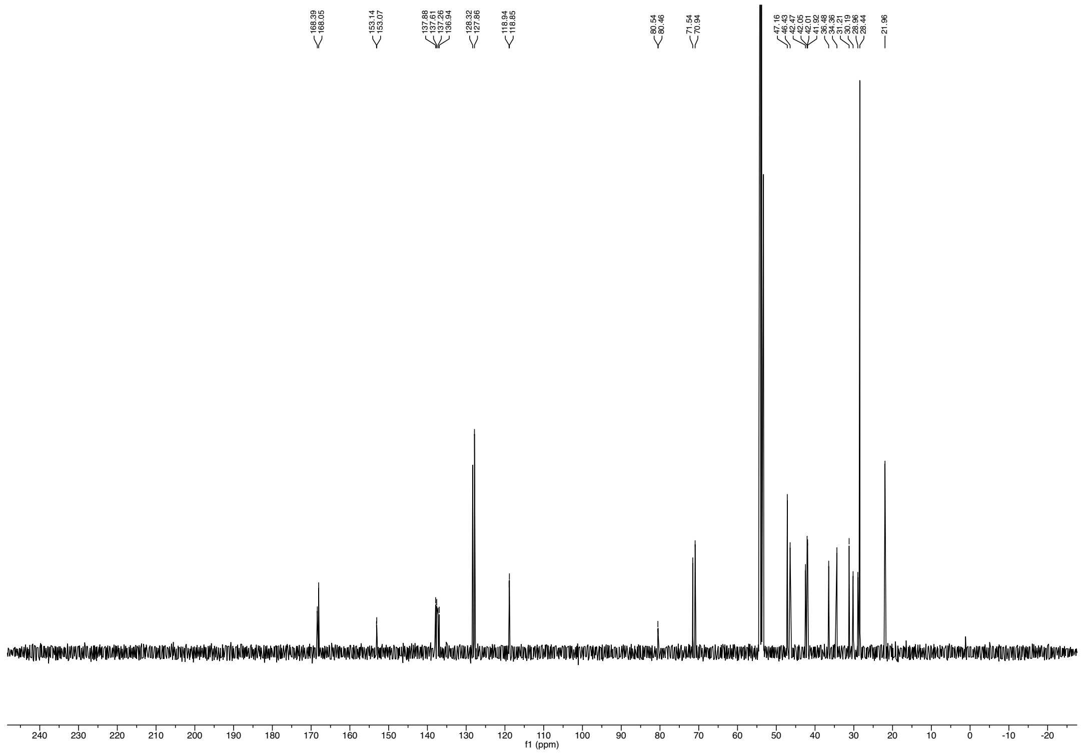
f1 (ppm)

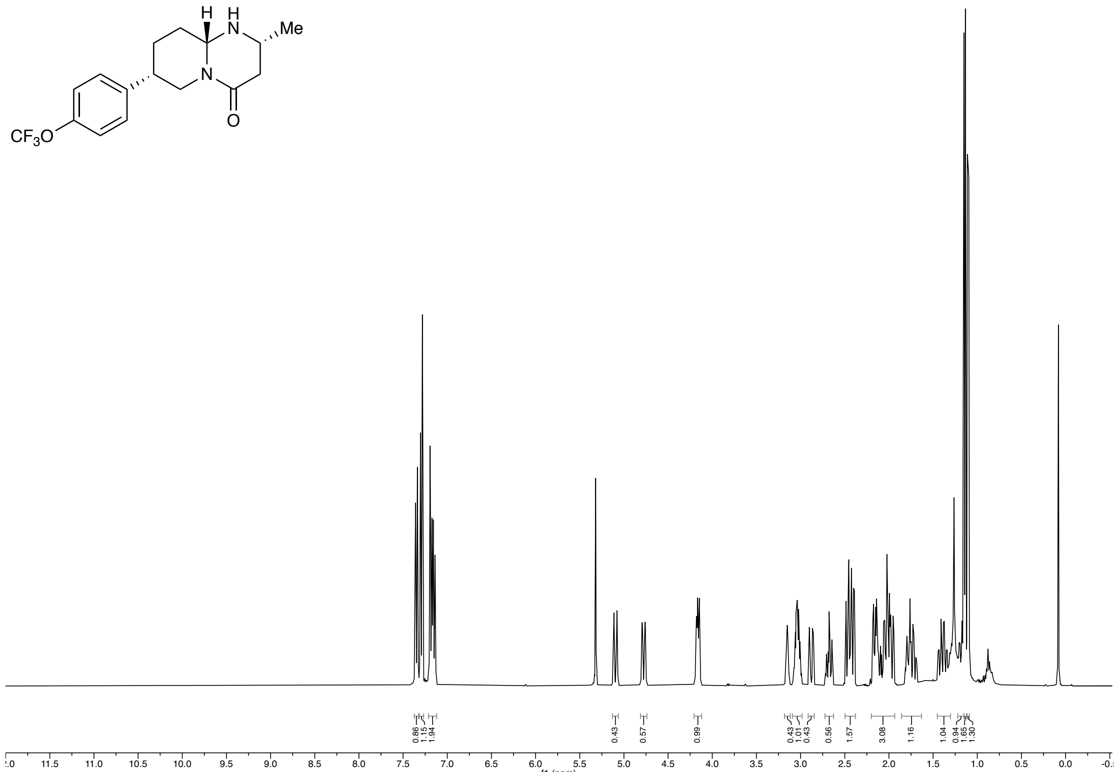
Me
CF3O
O
0.86
1.15
1.94
0.43
0.57
0.99
0.43
1.01
0.43
0.56
1.57
3.08
1.16
1.04
0.94
1.65
1.30
f1 (ppm)

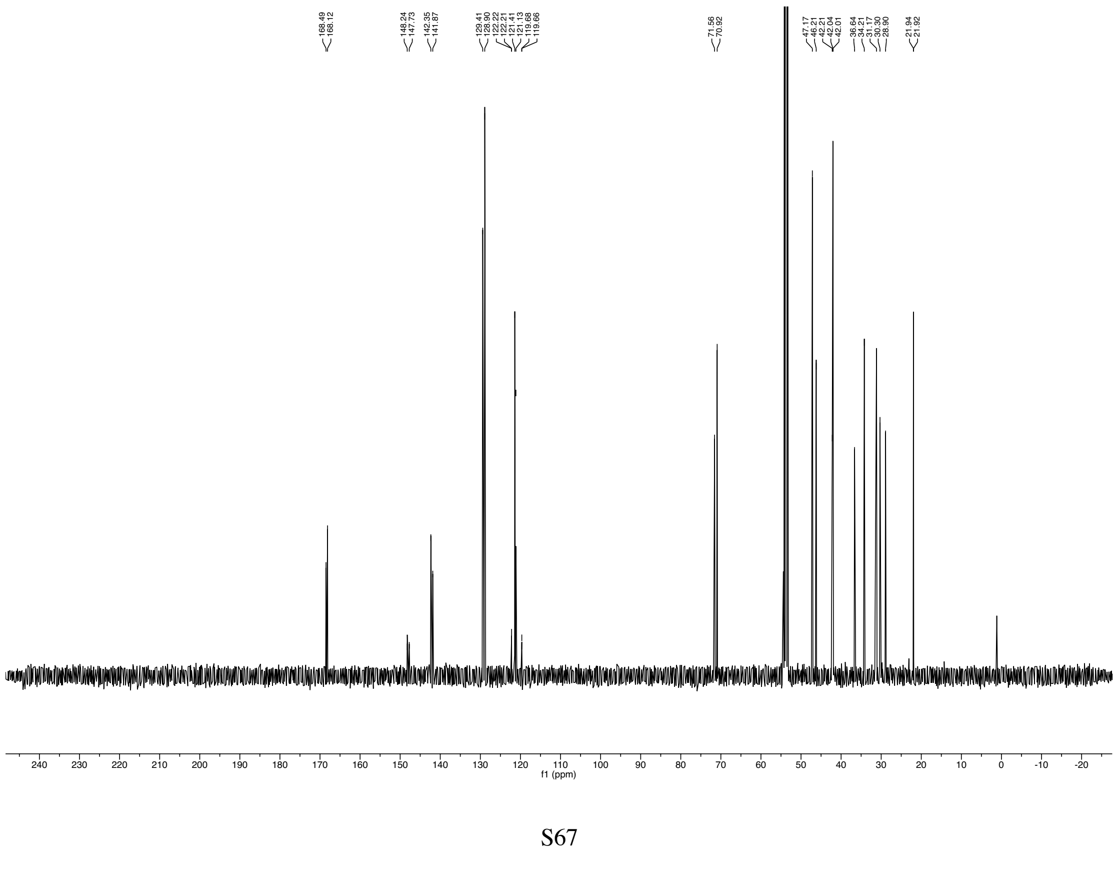
168.49
168.12
148.24
147.73
142.35
141.87
129.41
128.90
122.22
122.21
121.41
121.13
119.68
119.66
71.56
70.92
47.17
46.21
42.21
42.04
42.01
36.64
34.21
31.17
30.30
28.90
21.94
21.92
f1 (ppm)

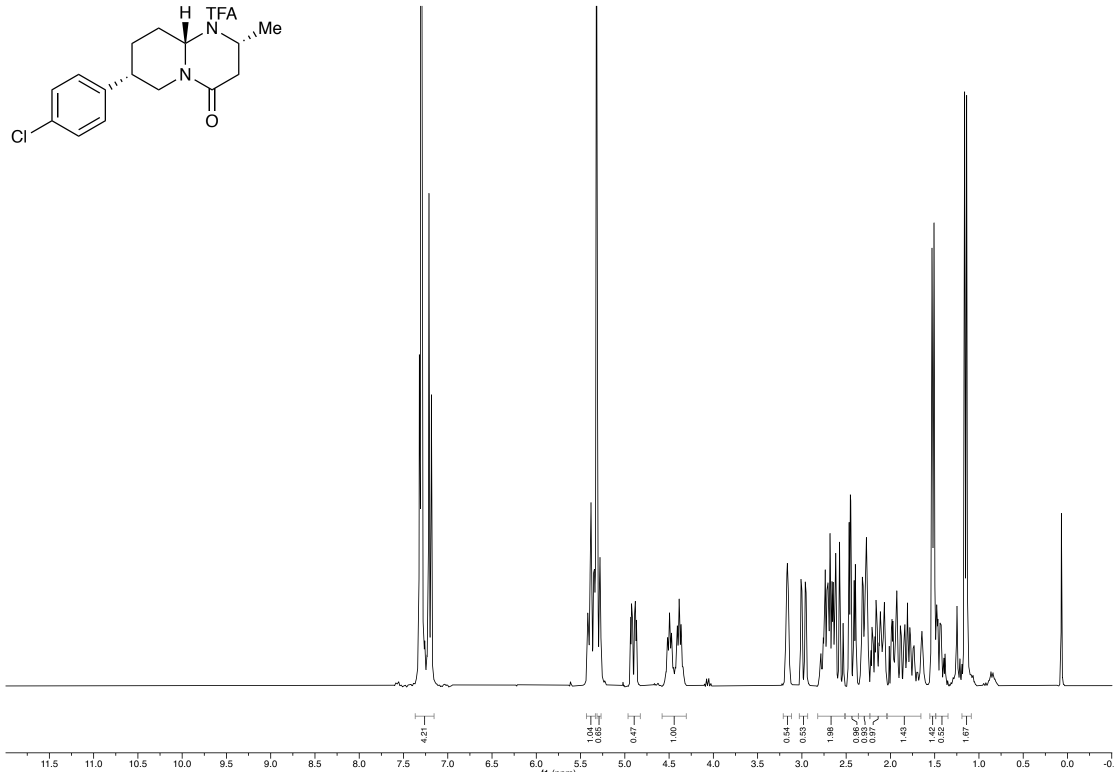
H
TFA
N
Me
N
O
Cl
4.21
1.04
0.65
0.47
1.00
0.54
0.53
1.98
0.96
0.93
0.97
1.43
1.42
0.52
1.67
f1 (ppm)

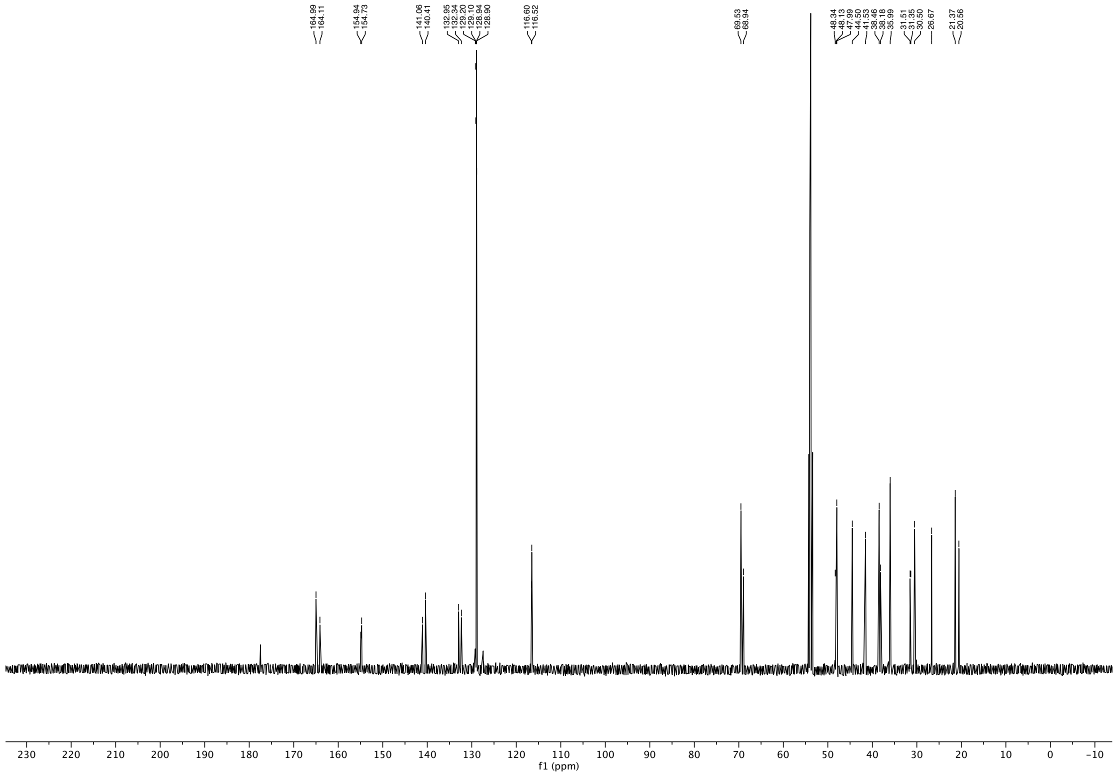
164.99
164.11
154.94
154.73
141.06
140.41
132.95
132.34
129.20
129.10
128.94
128.90
116.60
116.52
69.53
68.94
48.34
48.13
47.99
44.50
41.53
38.46
38.18
35.99
31.51
31.35
30.50
26.67
21.37
20.56
f1 (ppm)

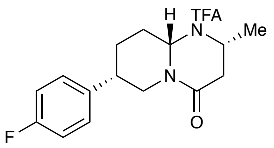
H
TFA
N
Me
N
O
F

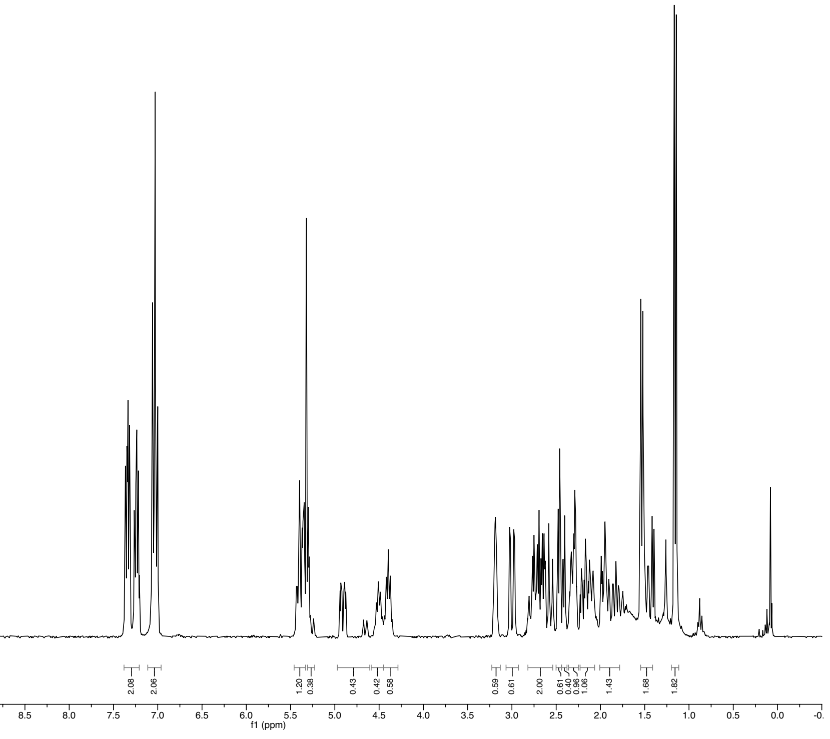
2.08
2.06
1.20
0.38
0.43
0.42
0.58
0.59
0.61
2.00
0.61
0.40
0.96
1.06
1.43
1.68
1.82
f1 (ppm)

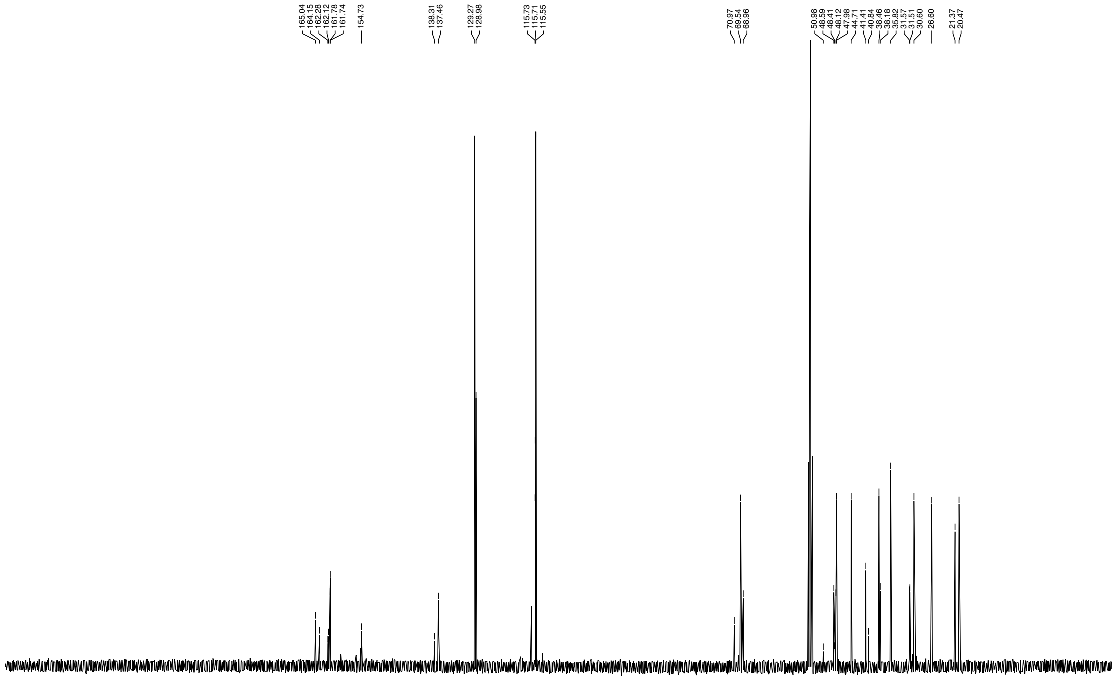
165.04
164.15
162.28
162.12
161.78
161.74
154.73
138.31
137.46
129.27
128.98
115.73
115.71
115.55
70.97
69.54
68.96
50.98
48.59
48.41
48.12
47.98
44.71
41.41
40.84
38.46
38.18
35.82
31.57
31.51
30.60
26.60
21.37
20.47
f1 (ppm)

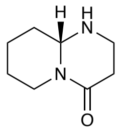
H
N
H
N
O

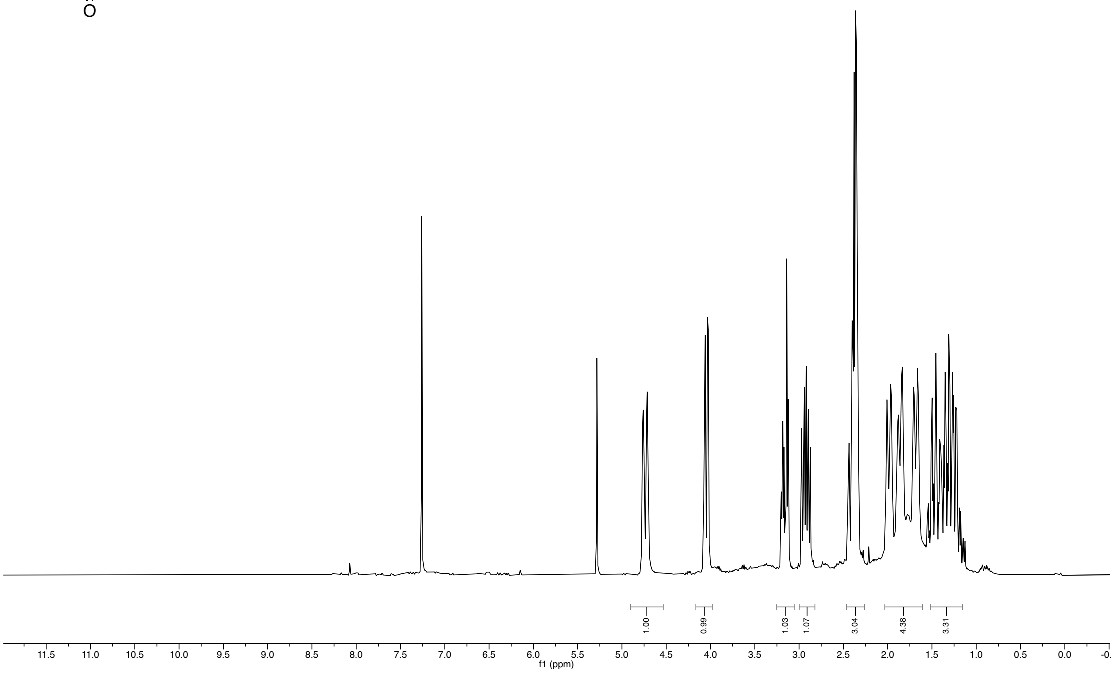
1.00
0.99
1.03
1.07
3.04
4.38
3.31
f1 (ppm)

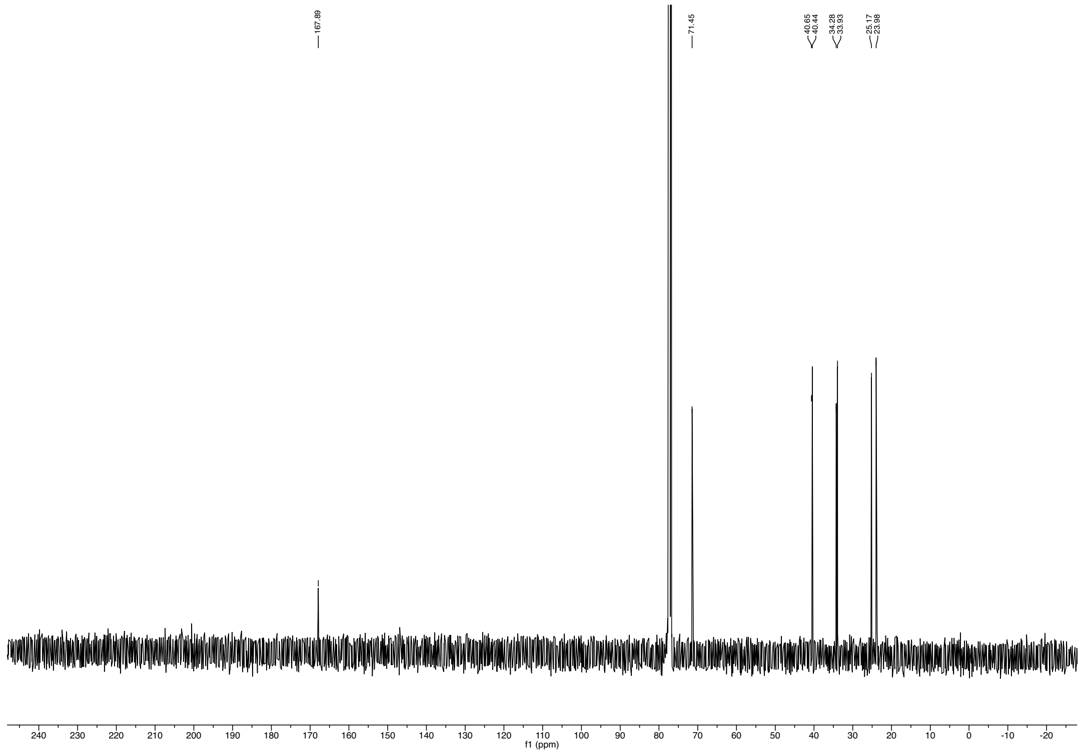
167.89
71.45
40.65
40.44
34.28
33.93
25.17
23.98
f1 (ppm)

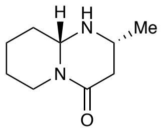
H
N
Me
N
O

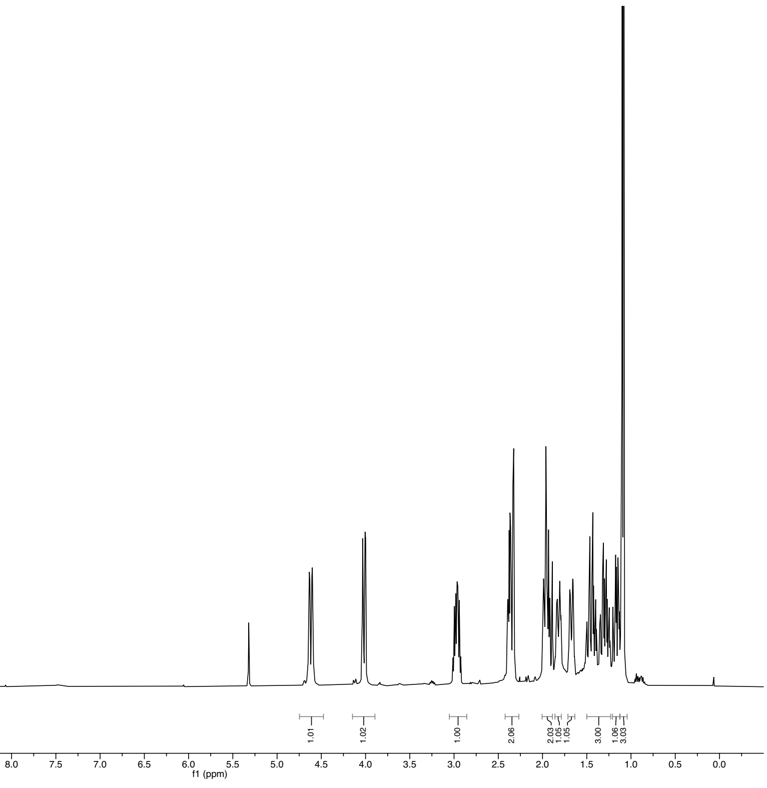
1.01
1.02
1.00
2.06
2.03
1.05
1.05
3.00
1.06
3.03
f1 (ppm)

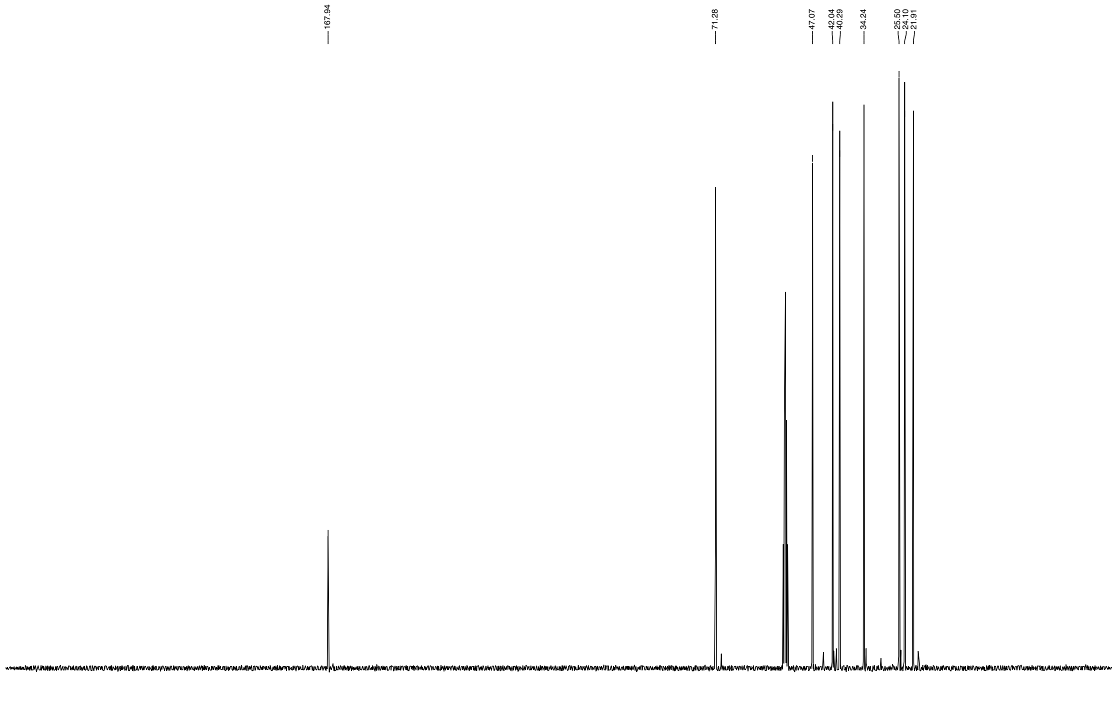
167.94
71.28
47.07
42.04
40.29
34.24
25.50
24.10
21.91
f1 (ppm)

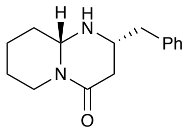
H
N
N
Ph
O

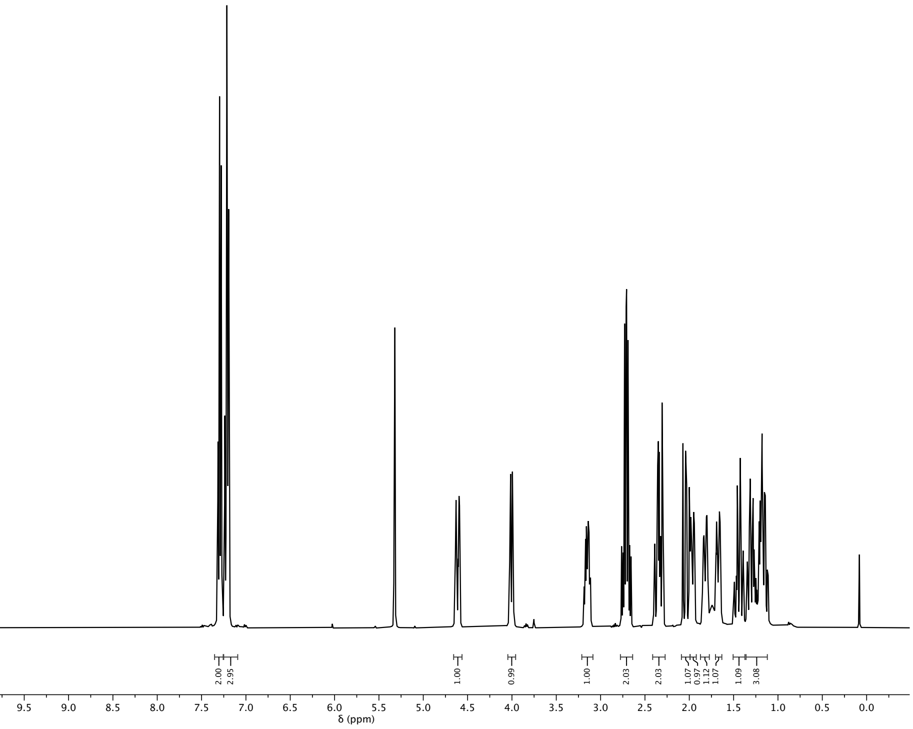
δ (ppm)

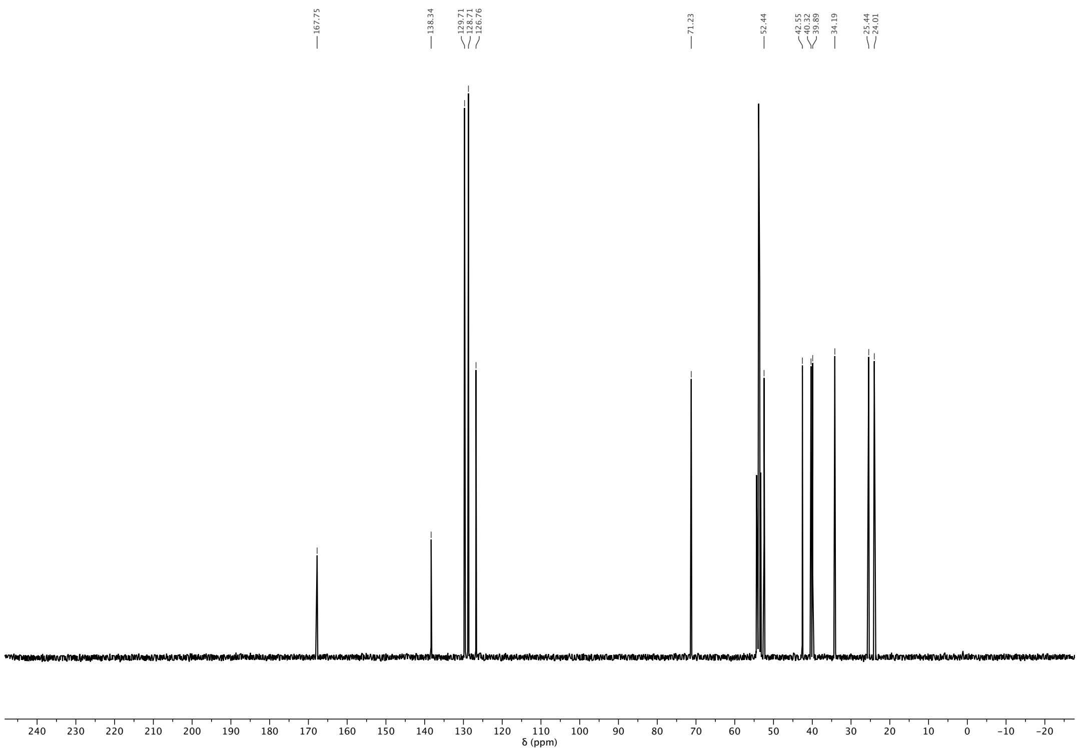
167.75
138.34
129.71
128.71
126.76
71.23
52.44
42.55
40.32
39.89
34.19
25.44
24.01
δ (ppm)

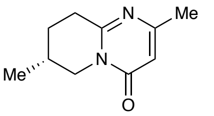
N
Me
Me
N
O

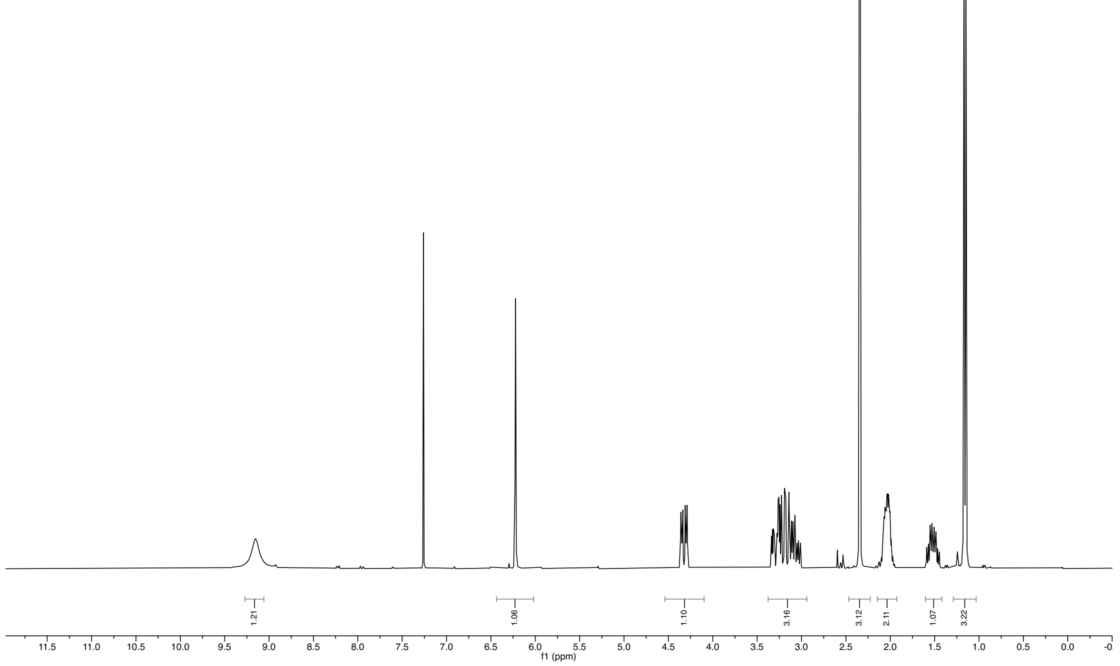
1.21
1.06
1.10
3.16
3.12
2.11
1.07
3.22
11.5 11.0 10.5 10.0 9.5 9.0 8.5 8.0 7.5 7.0 6.5 6.0 5.5 5.0 4.5 4.0 3.5 3.0 2.5 2.0 1.5 1.0 0.5 0.0 -0
f1 (ppm)

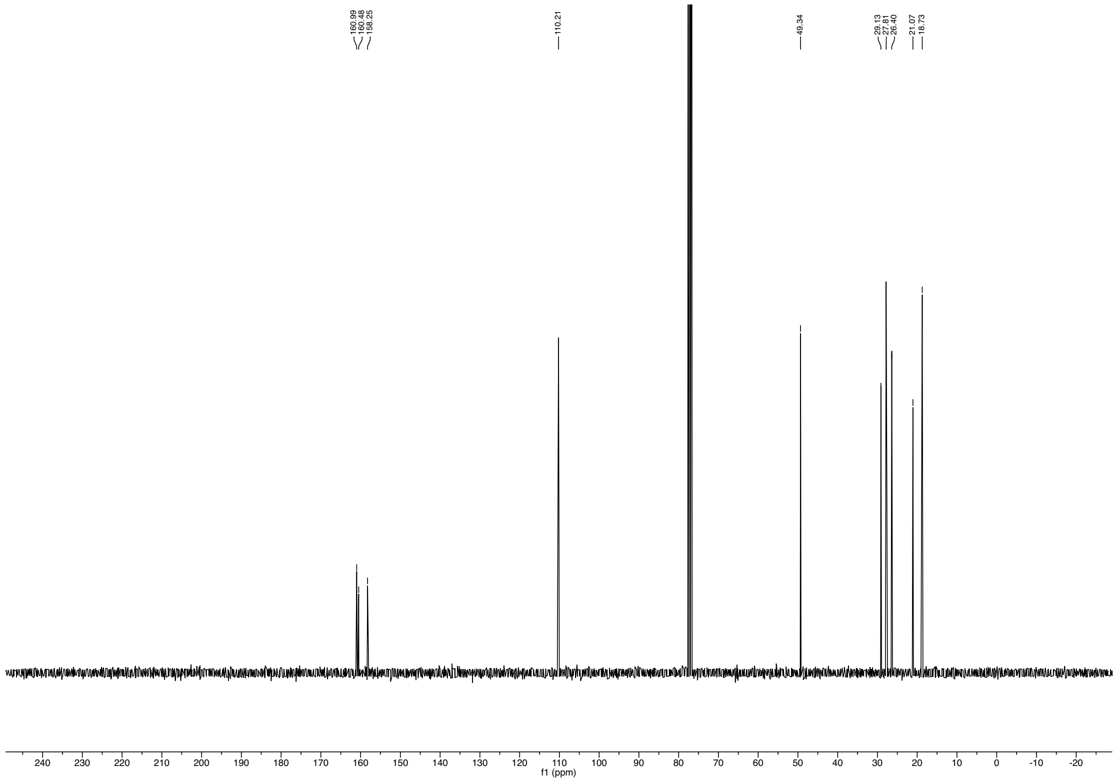
160.99
160.48
158.25
110.21
49.34
29.13
27.81
26.40
21.07
18.73
240 230 220 210 200 190 180 170 160 150 140 130 120 110 100 90 80 70 60 50 40 30 20 10 0 -10 -20
f1 (ppm)

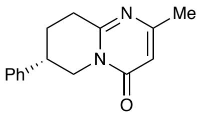
N
Me
N
Ph
O

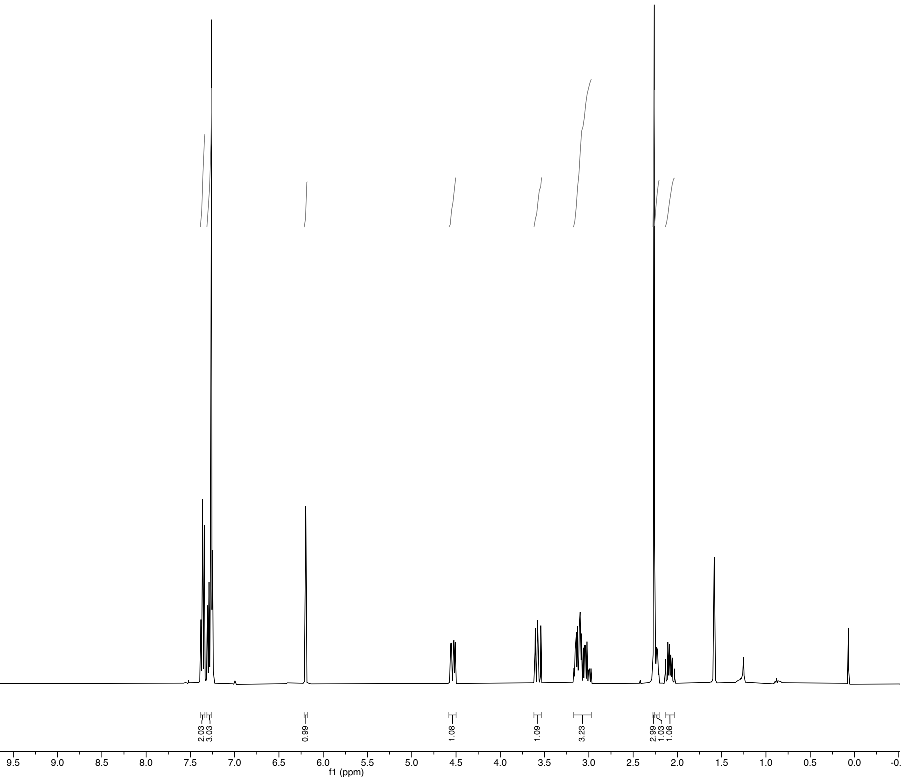
2.03
3.03
0.99
1.08
1.09
3.23
2.99
1.03
1.08
f1 (ppm)

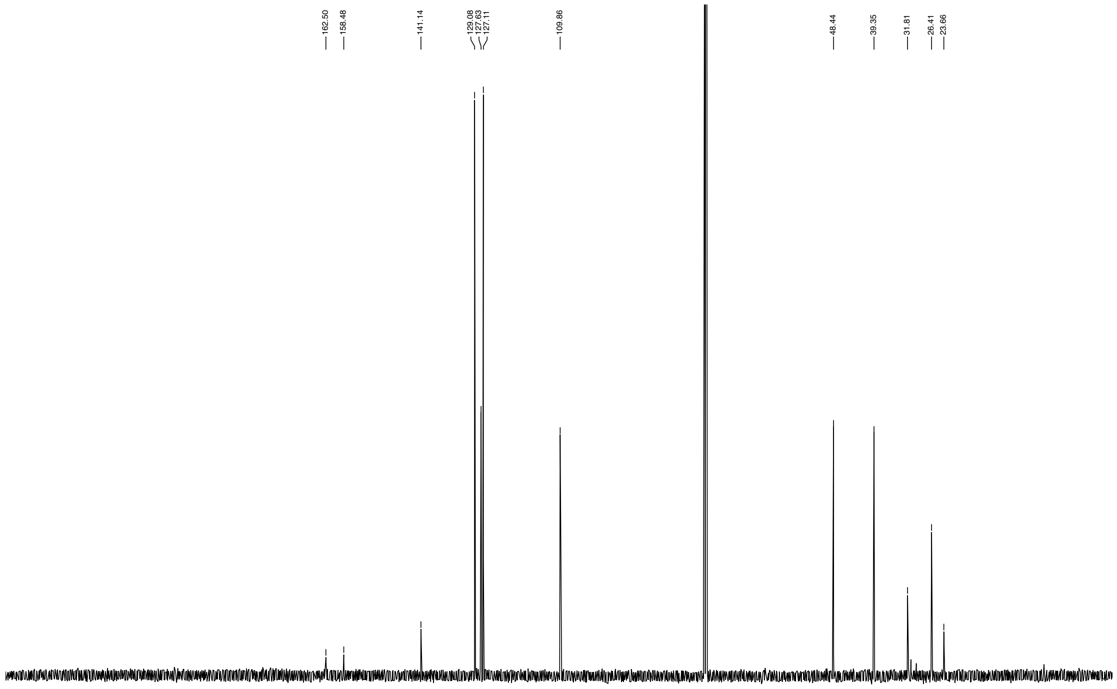
162.50
158.48
141.14
129.08
127.63
127.11
109.86
48.44
39.35
31.81
26.41
23.66
f1 (ppm)

(K) X-ray diffraction data

Molecular structure of **all-*cis*-4a** • 2 HCl

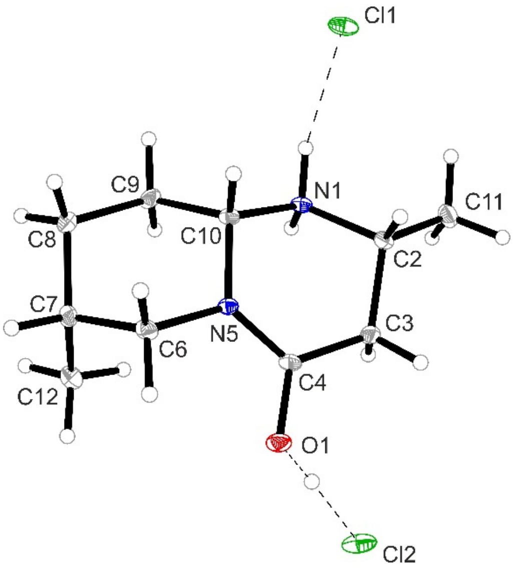

Figure S4. Crystal structure of compound **all-*cis*-4a**. Thermal ellipsoids are shown at 15% probability.

A colorless needle-like specimen of $C_{10}H_{20}Cl_2N_2O$, approximate dimensions 0.020 mm x 0.020 mm x 0.240 mm, was used for the X-ray crystallographic analysis. The X-ray intensity data were measured. The integration of the data using a monoclinic unit cell yielded a total of 1959 reflections to a maximum θ angle of 24.99° (0.84 Å resolution), of which 1959 were independent (average redundancy 1.000, completeness = 96.8%, R_{sig} = 3.96%) and 1802 (91.99%) were greater than 2σ(F^2). The final cell constants of a = 8.3482(5) Å, b = 6.0629(3) Å, c = 13.2849(9) Å, β = 106.642(2)°, volume = 644.24(7) $Å^3$, are based upon the refinement of the XYZ-centroids of reflections above 20 σ(I). The calculated minimum and maximum transmission coefficients (based on crystal size) are 0.8930 and 0.9900. The structure was solved and refined using the Bruker SHELXTL Software Package, using the space group $P2_1$, with Z = 2 for the formula unit, $C_{10}H_{20}Cl_2N_2O$.The final anisotropic full-matrix least-squares refinement on F^2 with 150 variables converged at R1 = 5.77%, for the observed data and wR2 = 15.17% for all data. The goodness-of-fit was 1.077. The largest peak in the final difference electron density synthesis was 0.324 $e^-/Å^3$ and the largest hole was −0.271 $e^-/Å^3$ with an RMS deviation of 0.072 $e^-/Å^3$. On the basis of the final model, the calculated density was 1.315 g/cm^3 and F(000), 272 e^-. Flack parameter was refined to: 0.12(13). CCDC Nr.: 2006232.

Molecular structure of ***trans-cis*-4a**

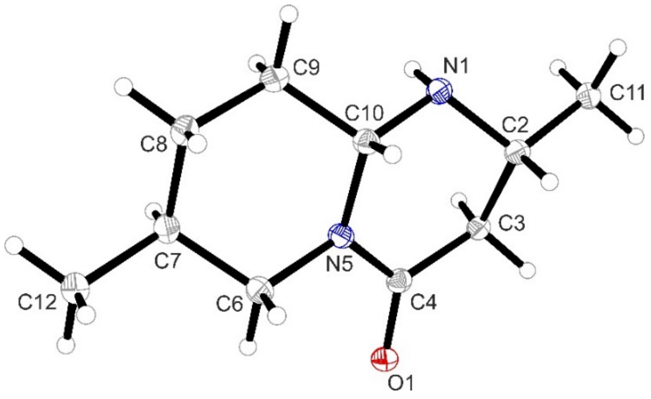

Figure S5. Crystal structure of compound ***trans-cis*-4a**. Thermal ellipsoids are shown at 30% probability.

A colorless prism-like specimen of $C_{10}H_{18}N_2O$, approximate dimensions 0.080 mm x 0.120 mm x 0.180 mm, was used for the X-ray crystallographic analysis. The X-ray intensity data were measured. A total of 1975 frames were collected. The total exposure time was 43.18 hours. The frames were integrated with the Bruker SAINT software package using a wide-frame algorithm. The integration of the data using a monoclinic unit cell yielded a total of 14466 reflections to a maximum θ angle of 66.72° (0.84 Å resolution), of which 1752 were independent (average redundancy 8.257, completeness = 99.3%, R_{int} = 6.43%, R_{sig} = 3.35%) and 1334 (76.14%) were greater than 2σ(F^2). The final cell constants of a = 9.8352(5) Å, b = 9.3209(4) Å, c = 11.4836(6) Å, β = 108.743(3)°, volume = 996.91(9) $Å^3$, are based upon the refinement of the XYZ-centroids of 3813 reflections above 20 σ(I) with 10.33° < 2θ < 132.7°. Data were corrected for absorption effects using the multi-scan method (SADABS). The ratio of minimum to maximum apparent transmission was 0.839. The calculated minimum and maximum transmission coefficients (based on crystal size) are 0.8960 and 0.9520. The structure was solved and refined using the Bruker SHELXTL Software Package, using the space group $P2_1/n$, with Z = 4 for the formula unit, $C_{10}H_{18}N_2O$. The final anisotropic full-matrix least-squares refinement on F^2 with 124 variables converged at R1 = 4.10%, for the observed data and wR2 = 10.17% for all data. The goodness-of-fit was 1.054. The largest peak in the final difference electron density synthesis was 0.171 $e^-/Å^3$ and the largest hole was −0.195 $e^-/Å^3$ with an RMS deviation of

0.041 $e^{-}/Å^{3}$. On the basis of the final model, the calculated density was 1.214 g/cm^{3} and F(000), 400 e^{-}. The hydrogen at N1 atom was refined freely. CCDC Nr.: 2006231.

(L) References

[1] Bruker AXS, Inc., APEX3 (Madison, Wisconsin, **2016**).

[2] Bruker AXS, Inc., SAINT (Madison, Wisconsin, **2015**).

[3] Bruker AXS, Inc., SADABS (Madison, Wisconsin, **2015**).

[4] G. M. Sheldrick, *Acta Crystallogr. A*, 2015, **71**, 3–8.

[5] G. M. Sheldrick, *Acta Crystallogr. C*, 2015, 3–8.

[6] R. W. W. Hooft, B. V.Nonius, COLLECT, Program for Collecting Data on CCD Area Detectors, 1998, Delft, The Netherlands.

[7] Z. Otwinowski, W. Minor, *Methods Enzymol.*, 1997, **276**, 307.

[8] Z. Otwinowski, D.Borek, W. Majewski, W. Minor, *Acta Crystallogr. A*, 2003, **59**, 228.

[9] Bruker AXS Inc., XP-Interactive molecular graphics, version 5.1 (Madison, Wisconsin, USA, **1998**).

[10] S. Urban, N. Ortega, *Angew. Chem., Int. Ed.*, 2011, **50**, 3803.

[11] L. Lengyel, T. Z. Nagy, G. Sipos, R. Jones, G. Dormán, L. Ürge, F. Darvas, *Tetrahedron Lett.*, 2012, **53**, 738.

[12] M. A. Hussein, M. A. Abdel-Rahman, A. A. Geies, *J. Appl. Polym. Sci.*, 2012, **126**, 2.

[13] A. Molnár, A. Kapros, L. Párkányi, Z. Mucsi, G. Vlád, I. Hermecz, *Org. Biomol. Chem.*, 2011, **9**, 6559.
